# A Multiple-Baseline Evaluation of Acceptance and Commitment Therapy Focused on Repetitive Negative Thinking for Comorbid Generalized Anxiety Disorder and Depression

**DOI:** 10.3389/fpsyg.2020.00356

**Published:** 2020-03-13

**Authors:** Francisco J. Ruiz, Carmen Luciano, Cindy L. Flórez, Juan C. Suárez-Falcón, Verónica Cardona-Betancourt

**Affiliations:** ^1^Faculty of Psychology, Fundación Universitaria Konrad Lorenz, Bogotá, Colombia; ^2^Departament of Psychology, Universidad de Almería, Almería, Spain; ^3^Madrid Institute of Contextual Psychology, Madrid, Spain; ^4^Department of Behavioral Sciences Methodology, Universidad Nacional de Educación a Distancia, Madrid, Spain; ^5^Universitat Oberta de Catalunya, Barcelona, Spain

**Keywords:** acceptance and commitment therapy, relational frame theory, depression, generalized anxiety disorder, repetitive negative thinking

## Abstract

Repetitive negative thinking (RNT) is a core feature of generalized anxiety disorder (GAD) and depression. Recently, some studies have shown promising results with brief protocols of acceptance and commitment therapy (ACT) focused on RNT in the treatment of emotional disorders in adults. The current study analyzes the effect of an individual, 3-session, RNT-focused ACT protocol in the treatment of severe and comorbid GAD and depression. Six adults meeting criteria for both disorders and showing severe symptoms of at least one of them participated in the study. A delayed multiple-baseline design was implemented. All participants completed a 5-week baseline without showing improvement trends in emotional symptoms (Depression Anxiety and Stress Scale – 21; DASS-21) and pathological worry (Penn State Worry Questionnaire; PSWQ). The ACT protocol was then implemented, and a 3-month follow-up was conducted. Five of the six participants showed clinically significant changes in the DASS-21 and the PSWQ. The standardized mean difference effect sizes for single-case experimental design were very large for emotional symptoms (*d* = 3.34), pathological worry (*d* = 4.52), experiential avoidance (*d* = 3.46), cognitive fusion (*d* = 3.90), repetitive thinking (*d* = 4.52), and valued living (*d* = 0.92 and *d* = 1.98). No adverse events were observed. Brief, RNT-focused ACT protocols for treating comorbid GAD and depression deserve further empirical tests.

## Introduction

### Comorbidity Between GAD and Depression

Generalized anxiety disorder (GAD) and unipolar depression are the two psychological disorders most frequently seen in primary care and outpatient mental health services (Wittchen, 2002). The lifetime prevalence of GAD is considered to be between 4 and 7% (Kessler, 2000; Wittchen and Hoyer, 2001), whereas for depression, the estimate reaches 16% (Kessler et al., 2003), although there is high variability across cultures (Kessler and Bromet, 2013). Both disorders lead to considerable disability, with depression alone being considered the first cause of disability worldwide (World Health Organization [WHO], 2017).

Both GAD and depression are considered to be chronic disorders. On the one hand, GAD is known to be a very chronic condition, with episode duration commonly averaging a decade or longer (Kessler, 2000) and with less than 20% of sufferers experiencing complete remission of their symptoms when not seeking treatment actively (Wittchen, 2002). On the other hand, although the episode duration of depression is considerably shorter than in GAD, the recurrence of episodes is very high, with at least 50% of individuals who recover from the first one having more in their lifetime (Kupfer et al., 1996). The percentage of recurrence increases to 80% after a history of two depressive episodes (Burcusa and Iacono, 2007).

The comorbidity between GAD and depression is more the rule than the exception (Klenk et al., 2011), with studies estimating it at up to 80% of the cases (Judd et al., 1998; Lamers et al., 2011). This comorbidity is associated with slower recovery and greater chronicity, recurrence rates, health costs, hospitalization rates, disability days, suicide attempts, and psychosocial disability (Hirschfeld, 2001; Wittchen, 2002). Additionally, depression and GAD are both relevant risk factors for the development of medical conditions such as vascular and pulmonary disease, lipid disorders, and asthma (Hirschfeld, 2001; Kroenke et al., 2007). The average annual cost of comorbid GAD and depression is about 4,235 USD per person and, when other somatoform disorders are developed, such as pain disorders, it increases to 12,624 USD (Zhu et al., 2009). Importantly, the presence of comorbid GAD and depression has predicted poorer therapeutic outcomes using psychotropic medication (Van Balkom et al., 2008) and psychological therapy (Newman et al., 2010; Coplan et al., 2015).

### Brief Interventions for GAD and Depression

There are at least two main reasons to emphasize the need for developing effective, brief interventions for comorbid GAD and depression. Firstly, premature psychotherapy termination is a frequent phenomenon in most clinical settings (Hilsenroth et al., 1995; Strosahl et al., 2012). For instance, the review conducted by Phillips (2014) found that between 40 and 55% of clients terminated the treatment between the first and second sessions. Other studies have found that the average number of sessions completed varies between four and six (Olfson et al., 2009), and the modal number of sessions was only one (Brown and Jones, 2005). Importantly, there is some evidence indicating that clients who terminated therapy prematurely have similar outcomes to the individuals who never began the therapy (Stark, 1992). Secondly, developing brief interventions is essential because psychological therapy provided in primary care settings for depression and GAD is usually brief due to the limited budget in mental health care or because health professionals opt to attend patients for brief, time-limited therapy to improve access to mental health services for all patients in the clinic (Saxena et al., 2007; Stiles et al., 2008; Robinson and Reiter, 2016).

The need for developing effective, brief interventions has been strongly emphasized within the concept of “minimal intervention needed for change” or MINC. This concept refers to “the minimal level of intervention intensity, expertise, and resources needed to achieve a clinically significant improvement” (Glasgow et al., 2014, p. 26). Accordingly, it is crucial to develop brief psychological interventions that can be realistically adopted in mental health services. However, the data of the efficacy of brief interventions obtained so far is not especially encouraging for comorbid GAD and depression. For instance, the meta-analysis conducted by Cape et al. (2010) revealed that, although brief interventions provided in primary care had similar efficacy than longer treatments for anxiety disorders, the effect sizes obtained for depression and mixed anxiety and depression were considerably smaller (*d* = 0.33 and 0.26, respectively). Therefore, there seems to be ample space for improving the efficacy of brief psychological interventions for comorbid depression and anxiety disorders.

### Common Transdiagnostic Processes Between GAD and Depression

A promising way to advance in the direction of developing psychological interventions based on the concept of MINC is to analyze the transdiagnostic processes involved in GAD and depression. In this sense, the high comorbidity between GAD and depression might be due to sharing some transdiagnostic processes such as worry (Borkovec, 1994), rumination (Nolen-Hoeksema, 2004), and experiential avoidance (Hayes et al., 1996). According to prospective and experimental studies, worry and rumination play a crucial role in the onset and maintenance of GAD and depression (Harvey A. G. et al., 2004; Ehring and Watkins, 2008). Whereas excessive worry is a core characteristic of GAD (Borkovec, 1994), rumination plays a significant role in depression (Nolen-Hoeksema, 2004). Due to the similarity between worry and rumination and the fact that individuals with GAD show high levels of rumination, and individuals with depression high levels of worry, the term repetitive negative thinking (RNT; Harvey A. G. et al., 2004; Ehring and Watkins, 2008) has been proposed to include both processes. Some therapeutic approaches aim to reduce engagement in unconstructive RNT, such as metacognitive therapy (MCT; Wells, 2009) and rumination-focused cognitive-behavioral therapy (RF-CBT; Watkins, 2016).

Functionally, worry and rumination can be conceptualized as experiential avoidance (EA) strategies. EA is a central construct in acceptance and commitment therapy (ACT; Hayes et al., 1999; Wilson and Luciano, 2002). It consists of a verbal regulation strategy involving deliberate efforts to avoid and escape from discomforting private experiences, even at the cost of behaving inconsistently with one’s values and goals (Hayes et al., 1996). EA strategies may take multiple topographies, but all pertain to the same functional class of behavior to the extent that they are directed at reducing discomfort. Empirical evidence supports the role of EA in the onset and maintenance of emotional disorders such as GAD and depression (Roemer and Orsillo, 2002; Boulanger et al., 2010; Ruiz, 2010).

### RNT-Focused ACT

A recent approach has explicitly integrated RNT and EA within a therapeutic approach called RNT-focused ACT (Ruiz et al., 2016a, 2018a). This approach is an attempt to provide ACT with a more in-depth focus on relational frame theory (RFT; Hayes et al., 2001) by incorporating recent theoretical and empirical analysis of its clinical applications (Ruiz et al., 2016a; Ruiz, 2019). Specifically, this approach includes, among others, (a) the RFT conceptualization of psychological flexibility (Törneke et al., 2016), (b) the conceptualization of values and triggers for RNT as hierarchical networks of positive and negative reinforcers (Gil-Luciano et al., 2019), (c) a temporal specification of experiential avoidance cycles (Ruiz et al., 2016a), (d) an RFT conceptualization of RNT (Ruiz, 2019), (e) and the theoretical and empirical analysis of the relational processes involved in defusion, self-as-context, and values components and metaphors (e.g. Luciano et al., 2011; Villatte et al., 2015; Sierra et al., 2016; Törneke, 2017; Gil-Luciano et al., 2017; López-López and Luciano, 2017; Criollo et al., 2018). For the sake of brevity, we will focus the exposition on the first four points.

The RNT-focused ACT approach highlights that values are hierarchical reinforcers, or higher-order meaning functions (Luciano, 2017), established in the individual’s learning history (Barnes-Holmes et al., 2004; Luciano et al., 2012). For instance, as indicated in Gil-Luciano et al. (2019), Figure 1 (right panel) depicts a hierarchical relational network of reinforcers in which the individual established that a “meaningful life” consists of developing friendships characterized by trust and sharing and enjoying literature by increasing knowledge and developing writing skills. Within each branch of the hierarchy, some objectives and actions acquire reinforcing functions because they are connected to the previously mentioned values. Similarly, the individual will derive a relational network in an opposite relation with the first one, which will be the “other side of the coin” of values (Gil-Luciano et al., 2019).

**FIGURE 1:**
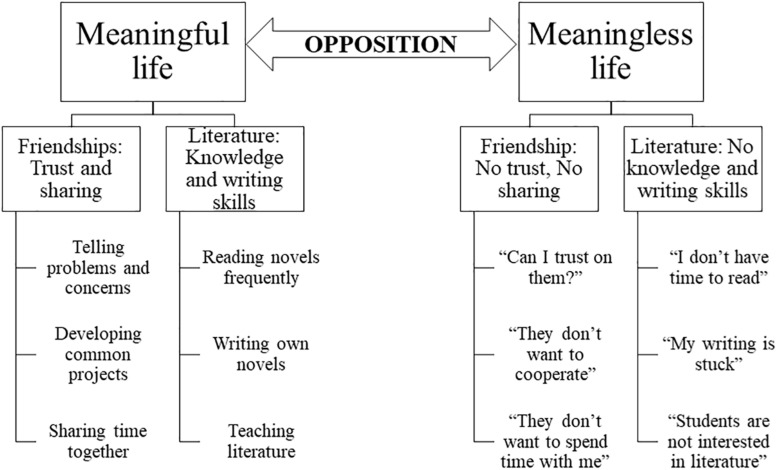
Example of a hierarchical network of positive reinforcers **(left)** and negative reinforcers that might function as triggers for engaging in RNT **(right)**.

For example, sharing a problem with a friend might have a reinforcing function, even when the person is re-experiencing negative feelings while telling it because it is part of developing intimate friendships. In contrast, events indicating an opposite result will acquire aversive functions. For instance, when being ignored by a friend, the person might derive the aversive thought, “I can’t trust him,” which is hierarchically related in opposition to the value of developing intimate friendships. The most important thing is that the individual will react in the presence of this thought with a particular aim.

Törneke et al. (2016) identified two main ways of responding to our own behavior. The first way is responding in coordination with the immediate discriminative or controlling functions of the thought. This way, when the thought is aversive, the reaction will have a function of avoidance. For example, the person might respond by yelling at the friend for not paying attention to the conversation or by ruminating about the friend’s behavior in an attempt to understand him. The second way is responding in hierarchy with the deictic “I,” which means discriminating that the thought is just a momentary event and to respond under the control of values (e.g. politely asking the friend why he is not paying attention). This pattern of behavior consisting of responding in hierarchy with the deictic “I” and redirecting attention to values is the basis of psychological flexibility, which is the main aim of ACT.

**FIGURE 2:**
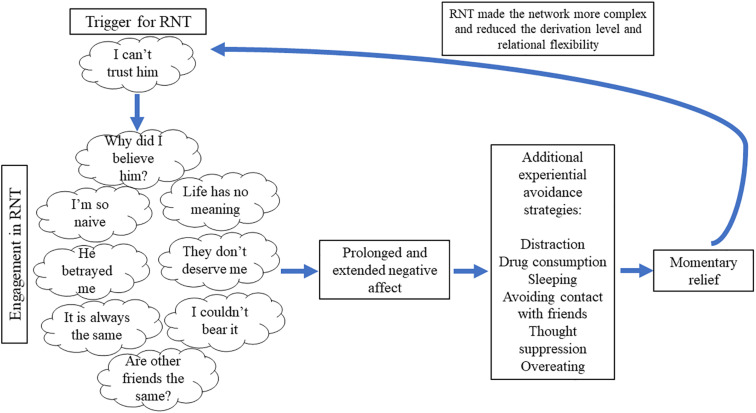
Description of a pernicious cycle of RNT and its consequences.

Throughout most learning histories, RNT usually becomes the predominant and first reaction in coordination to aversive experiences due to the sophisticated human’s linguistic abilities. In other words, individuals learn to react in less impulsive ways by engaging in RNT in order to reduce the discomfort caused by the thoughts and emotions (Côté et al., 2002). However, Figure 2 shows that RNT usually has a paradoxical effect because it prolongs negative affect as it focuses on negative content (Ehring and Watkins, 2008; Newman and Llera, 2011; Ruiz et al., 2016a). The prolonged negative affect usually leads the individual to engage in additional experiential avoidance strategies such as thought suppression, distraction, overeating, substance consumption, etcetera (e.g. Nolen-Hoeksema et al., 2007). These strategies usually provoke a reduction in the negative affect until new triggers for RNT surface.

RNT processes provoke at least three critical counterproductive effects. Firstly, the network of thoughts increases in complexity because myriads of new relations are established during the process (Barnes-Holmes et al., 2017). This effect facilitates the initiation of a new RNT process because more thoughts might begin to work as triggers (e.g. following Figure 2, the individual might engage in RNT when experiencing the thought, “Life has no meaning”). Secondly, the derivation level of the network of thoughts is reduced because of the repetition of the thinking process. This effect provokes that the individual will engage in a similar RNT process more rapidly and with higher automaticity, which resembles the common belief of uncontrollability of the RNT process among individuals with GAD and depression (e.g. Wells, 2009). Lastly, relational flexibility is reduced because thoughts are usually related in the same way during the RNT process. For instance, a particular thought of the RNT chain (e.g. “Why did I believe him?”) is most of the time followed by a related negative thought (e.g. “I’m so naïve”). The reduced relational flexibility provokes that the individual will experience more difficulties in disengaging from RNT by thinking in alternative ways, which resembles how these individuals tend to come back to thinking about the same thing over and over again.

In conclusion, the repetition of RNT cycles provokes that the individual’s life begins to focus more on RNT and the associated experiential avoidance strategies and less on his or her values. From this standpoint, GAD and depression are usually patterns of behavior characterized by RNT under the control of private experiences related in opposition to values. Accordingly, the aim of RNT-focused ACT is to develop the skill of disrupting unconstructive RNT in response to the hierarchical triggers and redirecting attention to valued actions (i.e. behavior under the control of hierarchical positive reinforcers). Focusing the intervention on the hierarchical triggers should provoke a more rapid and generalizable effect due to how the transformation of functions through hierarchical relations works (Gil et al., 2012, 2014; Gil-Luciano et al., 2019). This means that explicitly targeting the hierarchical triggers (e.g. fear of not developing the desired friendships) should produce the transformation of functions of thought integrated into it (e.g. “I can’t trust him”). However, targeting only specific thoughts contained in the hierarchical trigger might produce only a limited effect (Gil-Luciano, 2018).

### Empirical Evidence of RNT-Focused ACT

The empirical analysis of the RNT-focused ACT approach has followed a progressive strategy. The initial studies analyzed the feasibility of very brief, RNT-focused ACT protocols in emotional problems by employing single-case experimental designs (SCEDs). In the first study, Ruiz et al. (2016a) analyzed whether a 1-session protocol was sufficient to significantly reduce RNT in participants with mild to moderate emotional suffering. Eleven individuals participated in a two-arm, randomized multiple-baseline design. During the 6-week follow-up, nine participants showed significant reductions in most of the RNT measures. The intervention obtained very large effect sizes in all RNT-related measures and emotional symptoms. Ruiz et al. (2018a) conducted a second study analyzing the effect of a 2-session, RNT-focused ACT protocol in the treatment of 10 participants suffering from moderate emotional disorders. Nine participants showed clinically significant changes in emotional symptoms, with very large effect sizes (*d* = 2.44 and 2.68).

These initial studies encouraged testing brief RNT-focused ACT protocols with problems in which psychological interventions often find difficulties in reaching the usual level of efficacy. Ruiz et al. (2019) analyzed the efficacy of a 3-session protocol in three participants diagnosed with GAD, who indicated that the couple relationship was the main worry domain. The intervention obtained very a large effect size in reducing pathological worry (*d* = 3.19), and the three participants showed clinically significant changes. Similarly, Salazar et al. (2020) conducted a non-concurrent, multiple-baseline design with nine children with the main diagnosis of child depression and applied a 3-session protocol. No participant showed the diagnosis of child depression or comorbid disorders at the 4-week follow-up.

### Aim of the Study

This study aims to build upon previous studies by testing the efficacy of the 3-session, RNT-focused ACT protocol used in Ruiz et al. (2019) in participants suffering from comorbid GAD and depression. As previously discussed, testing the efficacy of brief approaches to treat comorbid GAD and depression is especially relevant due to the high prevalence of this comorbidity and the high disability, health risks, and economic costs that it provokes. A delayed multiple-baseline design was conducted with six adult participants who showed clinically significant levels of depression and GAD symptoms and at least severe symptom level in one of the disorders. The reporting of this SCED follows the SCRIBE statement (Tate et al., 2016).

## Materials and Methods

### Participants

The recruitment was conducted through advertisements in social media that included the following questions: “Do you spend too much time distressed about the past or the future? Do you want to be more focused on the things that are important to you?” Seventy-six individuals responded to the advertisements and answered an online survey. Initial inclusion criteria were: (a) being older than 18 years; (b) stating that they had experienced emotional difficulties for at least 12 months, (c) stating that symptoms significantly interfered in at least three life domains out of 8 (family, friendships/social relationships, training, work, couple relationship, leisure time, health, and spirituality), and (d) presenting severe scores in depression or anxiety according to the Depression Anxiety and Stress Scale – 21 (see the Outcome measures section). The interference of the emotional symptoms on life domains was measured by asking participants: “Please, select the life domains in which your sadness or anxiety is affecting your life.” The initial exclusion criterion was being in psychological or psychiatric treatment, including psychotropic medication.

Sixty-three potential participants were rejected according to the initial inclusion and exclusion criteria: five individuals were younger than 18 years, 19 were entangled with thoughts, memories, and worries for less than 1 year (they were invited to an alternative study if they experienced these problems for at least 6 months), 7 were receiving psychological or psychiatric treatment, and 32 did not show severe scores on depression and/or anxiety (they were invited to an alternative study). Of the remaining 13 potential participants, 5 did not respond to emails or did not attend the informative session. In conclusion, eight participants met the initial inclusion criteria and attended an interview conducted by a psychologist who had received extensive training in psychological assessment.

In this interview, the terms and conditions of the study were explained. Subsequently, the interviewer explored the presence of symptoms of GAD and depression based on the Mini International Neuropsychiatric Interview (MINI; Sheehan et al., 1998). The interviewer also asked questions related to the course of the emotional difficulties (e.g. How much time have you been feeling this way? How has feeling this way has affected your life? Have you been in psychological or psychiatric treatment? Have you experienced suicidal thoughts?). If the GAD and depression criteria were met, participants were asked to respond to two screening measures: the Personal Health Questionnaire – 9 (PHQ-9, Kroenke et al., 2001), which assesses depression, and the Generalized Anxiety Disorder – 7 (GAD-7, Spitzer et al., 2006), which assesses GAD. To participate in the study, individuals had to show clinically significant levels of depression and GAD symptoms (i.e. scores of at least 10 points in both the PHQ-9 and GAD-7) and a score of at least 15 points in the PHQ-9 or GAD-7. The latter score is the cutoff for the labels of “moderately severe” in the PHQ-9 and “severe” in the GAD-7 (note that the GAD-7 does not have the label “moderately severe”). Exclusion criteria were: (a) having suicidal thoughts more than half the days according to Item 9 of the PHQ-9; and (b) reporting frequent use of illegal drugs (e.g. marijuana, cocaine, etc.). Two participants were excluded: one because of experiencing suicidal thoughts more than half the days and one because of using illegal drugs frequently. Excluded participants were given the opportunity to receive immediate treatment or were directed to a mental health service. Participants who completed the study were remunerated with 25,000 Colombian pesos (approximately 8 United States dollars) as compensation for the intensive measurement carried out in the study.

The final sample consisted of 6 participants (2 men, mean age = 31.7, *SD* = 11.5). Table 1 shows demographic data of the participants, details of the problem, and the score range on the PHQ-9 and GAD-7. Pseudonyms are used throughout the manuscript. Participants showed a range of affected life areas between 4 and 6 and a problem duration between 4 and 25 years. Four participants had received psychological treatment in the past. Five of the six participants obtained severe scores on the PHQ-9 (*M* = 18.0, *SD* = 4.8). Likewise, five of the six participants obtained severe scores on the GAD-7 (*M* = 17.0, *SD* = 3.4).

**TABLE 1:** Demographic data, problem details, and scores on depression and generalized anxiety disorder.

**Participant**	**Sex**	**Age**	**Level of education**	**Life areas affected**	**Duration of the problem (years)**	**Previous therapy**	**PHQ-9**	**GAD-7**
Amelie	F	19	Under-graduate	5	6	Yes (public speaking fear)	19 (Mod. Severe)	17 (Severe)
Annie	F	33	Graduate	6	4	Yes (drug abuse and psychoanalysis to improve self-knowledge)	25 (Severe)	21 (Severe)
Marylin	F	29	Technician	4	10	Yes (hospitalized for severe depression and suicide attempt; suffering from fibromyalgia)	17 (Mod. Severe)	11 (Mod.)
Julia	F	34	Technician	4	4	No	19 (Mod. Severe)	16 (Severe)
Joaquin	M	52	Graduate	5	25	Yes (severe depression and anxiety)	18 (Mod. Severe)	18 (Severe)
Mateo	M	23	Under-graduate	4	10	No	10 (Mod.)	19 (Severe)

*Mod. = Moderate.*

### Design and Variables

The design of this study was a delayed multiple-baseline design across participants. The independent variable was the staggered introduction of a 3-session, RNT-focused ACT protocol. Following Kratochwill and Levin (2014), the minimum number of data points for the baseline was set at five. The protocol was implemented weekly. Afterward, a 12-week follow-up was conducted. Dependent variables were divided into outcome and process measures. As the main aim of this study was to explore the effect of the ACT protocol on treating comorbid depression and GAD, the outcome measures were scores on emotional symptoms (depression, anxiety, and stress) and pathological worry. Process measures were scores on experiential avoidance, cognitive fusion, valued living, and perseverative thinking. Since only one intervention was tested and the dependent measures were measured though automatic emails on the Internet, blinding procedures were not implemented.

### Outcomes Measures

#### Depression Anxiety and Stress Scales – 21

Depression Anxiety and Stress Scales – 21 [DASS-21; Lovibond and Lovibond, 1995; Spanish version by Ruiz et al. (2017a)]. The DASS-21 evaluates the negative emotional states experienced during the last week through 21-items with a 4-point Likert-type scale (3 = *applied to me very much or most of the time*; 0 = *did not apply to me at all*). The Spanish version of the DASS-21 has a hierarchical factor structure with three first-order factors (Depression, Anxiety, and Stress) and a second-order factor that is an overall indicator of emotional symptoms. The DASS-21 showed good internal consistency in Colombia, with Cronbach’s alphas of 0.93, 0.88, 0.83, and 0.83 for the DASS-Total, DASS-Depression, DASS-Anxiety, and DASS-Stress, respectively.

#### Penn State Worry Questionnaire – 11

Penn State Worry Questionnaire – 11 [PSWQ-11; Meyer et al., 1990; Spanish version by Ruiz et al. (2018b)]. The PSWQ is a measure of GAD-type worry. It consists of 11 items with a 5-point Likert-type scale (5 = *very typical of me*; 1 = *not at all typical of me*). The PSWQ-11 possesses excellent internal consistency in Colombia, with a Cronbach’s alpha of 0.95.

### Process Measures

#### Acceptance and Action Questionnaire – II

Acceptance and Action Questionnaire – II [AAQ-II; Bond et al., 2011; Spanish version by Ruiz et al. (2016b)]. The AAQ-II is a general measure of experiential avoidance and is one of the most used measures of ACT processes. It consists of seven items with a 7-point Likert-type scale (7 = *always true*; 1 = *never true*). The AAQ-II showed excellent psychometric properties in Colombia (Cronbach’s alpha of 0.91).

#### Cognitive Fusion Questionnaire

Cognitive Fusion Questionnaire [Gillanders et al., 2014; Spanish version by Ruiz et al. (2017b)]. The CFQ is a general measure of cognitive fusion, and, together with the AAQ-II, it is one of the most frequently used measures of ACT processes. It consists of seven items with a 7-point Likert-type scale (7 = *always true*; 1 = *never true*). The CFQ showed excellent psychometric properties in Colombia (Cronbach’s alpha of 0.93).

#### Perseverative Thinking Questionnaire

Perseverative Thinking Questionnaire (PTQ; Ehring et al., 2011). The PTQ is a content-independent measure of the tendency to engage in RNT when facing negative experiences or problems. It consists of 15 items with a 4-point Likert-type scale (4 = *almost always*; 0 = *never*). The PTQ has excellent internal consistency, high re-test reliability, and convergent and predictive validity. Preliminary data from our laboratory indicate that the PTQ possesses excellent internal consistency in Colombia (mean Cronbach’s alpha of 0.96).

#### Valuing Questionnaire

Valuing Questionnaire (VQ; Smout et al., 2014; Spanish version by Ruiz et al., submitted). The VQ is a measure of valued living averaged across life during the past week. It consists of 10 items with a 7-point Likert-type scale (6 = *completely true*; 0 = *not at all true*) and has two subscales: Progress and Obstruction. The Spanish version showed good psychometric properties in Colombia (Cronbach’s alphas of 0.83 and 0.82 for Progress and Obstruction, respectively).

### Screening Measures

#### Personal Health Questionnaire – 9

Personal Health Questionnaire – 9 (PHQ-9; Kroenke et al., 2001). The PHQ-9 is a screening and severity measure of depression according to the criteria of the Diagnostic and Statistical Manual of Mental Disorders – IV-TR (DSM-IV-TR; American Psychiatric Association, 2000). It consists of a 9-item with a 4-point Likert-type scale (3 = *nearly every day*; 0 = *not at all*). The ranges for mild, moderate, moderately severe, and severe are scores of 5–9, 10–14, 15–19, and 20–27, respectively. We used the Spanish translation of the PHQ-9 for Colombia distributed by Pfizer, which showed good psychometric properties in initial studies in our laboratory with clinical (α = 0.86) and non-clinical samples (α = 0.89), and a one-factor structure.

#### Generalized Anxiety Disorder – 7

Generalized Anxiety Disorder – 7 (GAD-7; Spitzer et al., 2006). The GAD-7 is a screening and severity measure of GAD according to the diagnostic criteria of the DSM-IV-TR (American Psychiatric Association, 2000). It consists of 7 items with a 4-point Likert-type scale (3 = *nearly every day*; 0 = *not at all*). The score ranges for mild, moderate, and severe levels of GAD are 5–9, 10–14, and 15–21, respectively. We used the Spanish translation of the GAD-7 for Colombia distributed by Pfizer, which showed good psychometric properties in initial studies in our laboratory with clinical (α = 0.87) and non-clinical samples (α = 0.90), and a one-factor structure.

### RNT-Focused ACT Protocol

The 3-session RNT-focused ACT protocol was the same used in Ruiz et al. (2019). The first session had a duration of approximately 90 min, and the second and third sessions lasted about 60 min. The protocol was based on the conceptualization of psychological flexibility in RFT terms (Luciano et al., 2012; Törneke et al., 2016) and aimed at developing the ability to discriminate ongoing triggers for worry/rumination, take distance from them (i.e. defusion), and behave according to what is most important at that moment for the individual in the long term (i.e. values).

The complete description of the protocol is available at https://bit.ly/2rq5Vps (Supplementary Presentations S1, S2). The aims of Session 1 were: (a) to present the rationale of the intervention, (b) to identify the main triggers to engage in RNT and the experiential avoidance strategies associated with RNT, (c) to promote realizing the pernicious effect of engaging in RNT and related experiential avoidance strategies, and (d) to promote the identification of the RNT process and to train in defusion. The aims of Session 2 were: (a) to review the advances since the first session, (b) multiple-exemplar training in identifying triggers for RNT and defusing from them using deictic, hierarchical framing and regulatory functions, and (c) to identify further valued actions to engage in as an alternative to engaging in RNT. Lastly, the aims of Session 3 were: (a) to review the advances since the second session, (b) values clarification through several experiential exercises, (c) to plan committed action, and (d) to close the intervention. Sessions 1 and 2 were the same as the protocol used in Ruiz et al. (2018a), with the exception that Session 1 in this study had a more extensive introduction of the intervention rationale and included explicit interactions consisting of framing ongoing behavior in hierarchy with the deictic “I.”

During the intervention, participants received five audio files (30 min approximately) to practice what was worked on during the sessions daily. The audio file provided at the end of Session 1 aimed at developing the skill to notice the difference between engaging and not engaging in RNT and letting the triggers be, while choosing to behave in a valued direction. Participants were given three audio files at the end of Session 2, similar to the three first exercises conducted: (a) the centering/defusion exercise, (b) the free association exercise, and (c) the “daydreaming and worrying exercise.” At the end of Session 3, participants received an additional audio file with a values exercise that summarized the content of this session.

All sessions were videotaped to analyze protocol integrity. Two independent observers with training in RNT-focused ACT protocols were given a complete version of the protocol. They were asked to review the sessions and identify if the therapist addressed the content of the epigraphs of the three sessions. Both observers indicated that the protocol was followed accurately in all cases.

### Procedure

The study was implemented in the Clinical Psychology laboratory of a Colombian university. The Institutional Ethics Committee approved the procedures of the study. The self-report described above were administered online through Typeform^1^. Participants who met the initial inclusion criteria were invited to an assessment and informative session led by the third author. When the individuals met the final inclusion criteria, the study functioning was introduced, and all informed consents were signed (all individuals agreed to participate). Afterward, participants responded to the first baseline evaluation. Participants provided baseline data every week for four weeks. The recruitment process was prolonged for 2 months, but the study began for the participants as soon as they attended the informative session and signed the informed consent. The latter decision was made for two reasons: (a) the limited number of potential participants due to the relatively restrictive inclusion criteria, and (b) ethical considerations regarding the severity of the participants’ psychological disorders. During the baseline, participants were monitored every week. It was determined with the participants that, if they experienced an extraordinary increase in emotional difficulties, the baseline would be finished, and the intervention would begin immediately.

The Theil-Sen slope (Sen, 1968) was computed at the end of the baseline collection with each participant to explore whether there were statistically significant tendencies during the baseline. All participants were scheduled to initiate the protocol implementation because there were no significant tendencies in the baselines for the outcome measures. The protocol was introduced weekly and in an individual format. During the intervention, participants were also assessed every week during the intervention and every 2 weeks during the 3-month follow-up. The first author, who is an experienced ACT therapist and has served as a therapist in other ACT studies, implemented the protocols in all cases.

After conducting the 3-month follow-up, participants were invited to an interview to close the study. In this interview, they were offered additional intervention if necessary and were explicitly asked if they received additional therapeutic support (e.g. psychotropic medication or psychotherapy) during the follow-up. No participant felt that it was necessary to receive further sessions, and none of them received additional therapeutic support during the follow-up. When finishing this interview, participants were compensated for their participation in the study.

### Data Analysis

**FIGURE 3:**
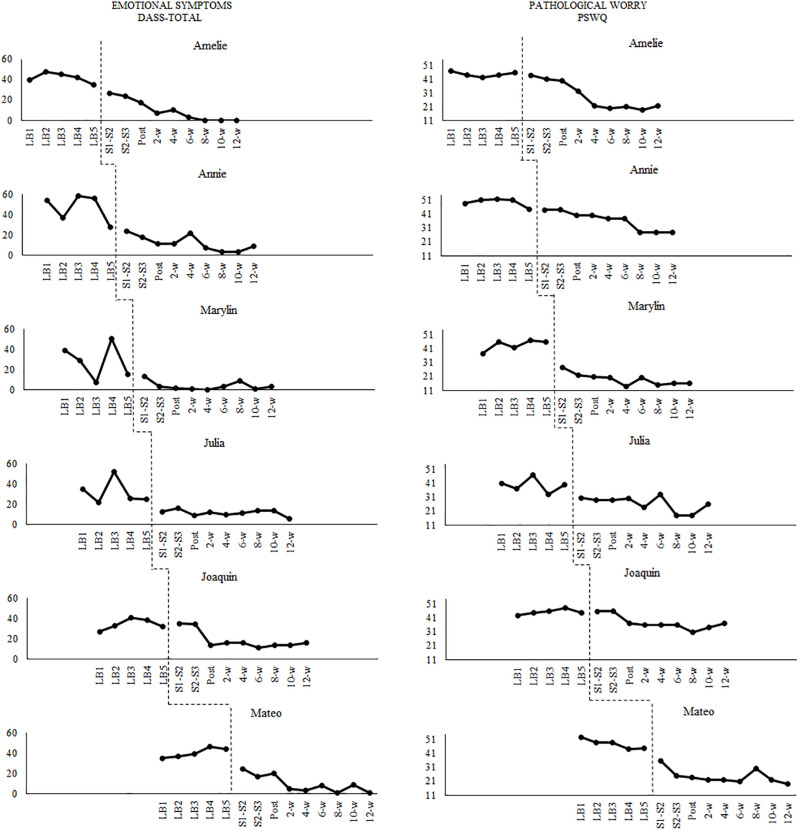
Participants’ evolution in emotional symptoms and pathological worry across the study.

The raw data of this study are accessible at https://bit.ly/2D9YvcG (Supplementary Data Sheet S1). Before implementing the interventions, participants’ baselines in primary outcome variables (DASS-Total and PSWQ) were graphed to explore scores’ stability and trends (see Figure 3). Scores on the PSWQ were quite stable across baselines (Participants 3 and 6 showed slight deteriorating and improvement trends, respectively). In contrast, more variability was present in the baseline of the DASS-21 scores (only Participant 6 showed a slight deteriorating trend). Although the presence of variability is not ideal, it is frequent in clinical datasets. However, variability is only an issue if it does not permit the observation of the intervention effect (Hayes, 1981). This was not the case in the current study.

The data analyses conducted in this study are similar to the ones described in detail in Ruiz et al. (2018a). Accordingly, we will only present a summary of them. The non-parametric Theil-Sen slope (Sen, 1968; Vannest et al., 2012) was computed using the online calculator provided by Vannest et al. (2011) to assess the presence of statistically significant trends in the baseline. No statistically significant trends in outcomes were found in baselines (if that were the case, the participant would have received the intervention, but his or her data would be excluded from the study). After collecting all data of the study, a bottom-up analysis of single-case experimental designs (SCED) was conducted (Parker and Vannest, 2012). After introducing the intervention, the data showed significant change levels and/or improvement trends. Most scores reached stability at the last three follow-up observations. Accordingly, we computed the within-participant statistical analyses taking into account all baseline data and only the last three follow-up points.

We selected the JZS + AR Bayesian hypothesis testing for SCED (de Vries and Morey, 2013, 2015) to analyze the within-participant results. The JZS + AR Bayesian model accounts for the serial dependence typical of SCEDs with an autoregressive [AR(1)] model. This method yields a *Bayes factor* (*B*_*ar*_) that quantifies the relative evidence in the data for the hypothesis of intervention effect (i.e. the true means of both phases differ: *B_*ar*_* > 1) and for the hypothesis of no intervention effect (i.e. the true mean in the baseline equals the true mean in the intervention phase: *B*_*ar*_ < 1). According to Jeffreys (1961), Bayes factors are interpreted with the following ranges: 1 = No evidence of treatment effect; 1–3 = Anecdotal evidence of treatment effect; 3–10 = Substantial evidence of treatment effect; 10–30 = Strong evidence of treatment effect; 30–100 = Very strong evidence of treatment effect; and 100 = Extreme evidence of treatment effect. Values of *B*_*ar*_ lower than one are interpreted similarly but in favor of the hypothesis of no treatment effect.

The JZS + AR model estimates two main parameters: (a) an effect size that it is named δ and consists of standardizing the difference in true means between phases; and (b) a parameter called *b* for the lag 1 (*ð*) autocorrelation. As in Ruiz et al. (2018a), we selected a Cauchy distribution with *r* = 1 for δ and *b* = 5 for the lag 1 autocorrelation and conducted sensitivity analysis (Gelman et al., 2014) that investigated the robustness of the results with *r* values of 0.5 and 2.0 (see Supplementary Table S1). The BayesSingleSub R package (de Vries and Morey, 2015) was used to compute these analyses. Since previous studies showed a high degree of efficacy of RNT-focused ACT protocols (Ruiz et al., 2016a, 2018a), we computed a one-sided Bayes factor that tested the null hypothesis that δ = 0 against the alternative hypothesis that δ > 0. According to de Vries et al. (2016), a clinically significant change requires a *B*_*ar*_ > 3 and crossing a cutoff point in the last follow-up measure that places the participant closer to the mean of the functional population than to the clinical one.

The between-case standardized mean difference effect size for SCED was computed to obtain overall estimates of the effect size of the intervention for each dependent variable at the 12-week follow-up (Pustejovsky et al., 2014). This d-statistic was derived from statistical theory and has known distribution, standard error, and significance test. Importantly, this effect-size measure is in the same metric as the Cohen’s d used in between-groups, randomized designs (see the mathematical developments in Hedges et al., 2012, 2013; Pustejovsky et al., 2014). This statistical method models single-case data with a hierarchical linear model, uses restricted maximum likelihood estimation, takes into account the autocorrelation typical in SCED, and corrects small sample bias using Hedges’ *g*. Three or more participants are necessary to compute this method in multiple-baseline designs. The d-statistic for SCED was computed according to the guidelines provided by Valentine et al. (2016) using the R package scdhlm (Pustejovsky, 2016). As this method accounts for potential trends in both the baseline and intervention, all data were used to compute the d-statistic. Standard guidelines suggest that values of *d* between 0.20 and 0.49 represent a small effect size, values between 0.50 and 0.79 represent a medium effect size, and values of 0.80 or higher represent large effect sizes (Cohen, 1992).

## Results

### Within-Participant Results

Figure 3 shows the evolution of the scores on the outcome measures. There were no significant trends in the baselines. The visual analysis shows that the ACT protocol was effective in decreasing emotional symptoms (DASS-21 total scores) and pathological worry (PSWQ scores) in all participants. For the sake of space, the figures corresponding to the process measures can be accessed at https://osf.io/d9cyp/ (Supplementary Figures S1–S5). Only one statistically significant trend was found for Amelie in AAQ-II scores according to the Theil-Sen slope analysis. Visual inspection shows that the intervention was effective in decreasing experiential avoidance, cognitive fusion, and RNT in all participants. Regarding the measures of valued living, the intervention was visually effective for five participants in the VQ-Progress, and all participants in the VQ-Obstruction.

Table 2 shows the main results of the JZS + AR Bayesian model. Five out of 6 participants showed at least substantial evidence of an intervention effect according to Bayes factors in overall emotional symptoms (i.e. DASS-total scores), whereas all participants showed substantial evidence in pathological worry (i.e. PSWQ scores). All participants showed evidence for an intervention effect in experiential avoidance (i.e. AAQ-II scores), cognitive fusion (i.e. CFQ scores), and repetitive negative thinking (i.e. PTQ scores). Lastly, 2 and 3 participants showed at least substantial evidence of change in values progress (i.e. VQ-Progress) and obstruction (i.e. VQ-Obstruction), respectively.

**TABLE 2:** Results of the JZS + AR analysis and clinically significant change for each participant and measure with a prior distribution of *r* = 1.

		**Amelie**	**Annie**	**Marylin**	**Julia**	**Joaquin**	**Mateo**
**Primary outcome measures**							
DASS – Total (emotional symptoms)	δ	8.6	2.6	1.0	1.3	3.3	6.6
	*B*_*ar*_	>**100**	**14.5**	2.1	**3.2**	**31.7**	>**100**
	*CSC*	**YES**	**YES**	NO	**YES**	**YES**	**YES**
PSWQ (pathological worry)	δ	11.0	7.6	7.2	2.7	4.0	4.2
	*B*_*ar*_	>**100**	>**100**	>**100**	**17.8**	**73.5**	**73.1**
	*CSC*	**YES**	**YES**	**YES**	**YES**	NO	**YES**
**Secondary outcome measures**							
AAQ-II (experiential avoidance)	δ	7.7	4.5	3.7	2.6	2.9	3.0
	*B*_*ar*_	>**100**	**80.1**	**44.5**	**16.2**	**23.5**	**24.9**
	*CSC*	**YES**	NO	**YES**	**YES**	NO	**YES**
CFQ (cognitive fusion)	δ	23.9	5.9	2.1	2.7	2.6	3.0
	*B*_*ar*_	>**100**	>**100**	**9.5**	**16.7**	**15.5**	**22.3**
	*CSC*	**YES**	**YES**	**YES**	**YES**	NO	**YES**
PTQ (perseverative thinking)	δ	5.8	5.1	4.2	5.2	7.1	2.4
	*B*_*ar*_	>**100**	>**100**	**80.6**	>**100**	>**100**	**12.3**
	*CSC*	**YES**	**YES**	**YES**	**YES**	NO	**YES**
VQ – Progress values	δ	8.6	0.9	0.3	0.2	3.5	0.1
	*B*_*ar*_	>**100**	2.0	0.8	0.6	**39.6**	0.5
	*CSC*	**YES**	NO	NO	NO	**YES**	NO
VQ – Obstruction values	δ	2.5	1.7	0.6	1.0	2.2	0.6
	*B*_*ar*_	**11.5**	**5.6**	1.2	2.0	**9.8**	1.2
	*CSC*	**YES**	**YES**	NO	NO	**YES**	NO

*B_ar_ = Bayes Factors of the JZS + AR model. B_ar_ > 1 supports the hypothesis of intervention effect. B_ar_ > 3 are in bold to highlight where at least substantial evidence of treatment effect was found.*

Table 2 also shows the results of clinically significant change (CSC) in outcome and process measures. Concerning primary outcome measures, 5 out of 6 participants showed CSC in DASS-total and PSWQ. Also, 5 of 6 participants showed CSC in CFQ and PTQ. For the AAQ-II scores, 4 of 6 participants showed CSC. Lastly, 2 and 3 participants showed CSC in VQ-Progress and VQ-Obstruction, respectively.

### Across-Participant Results

**FIGURE 4:**
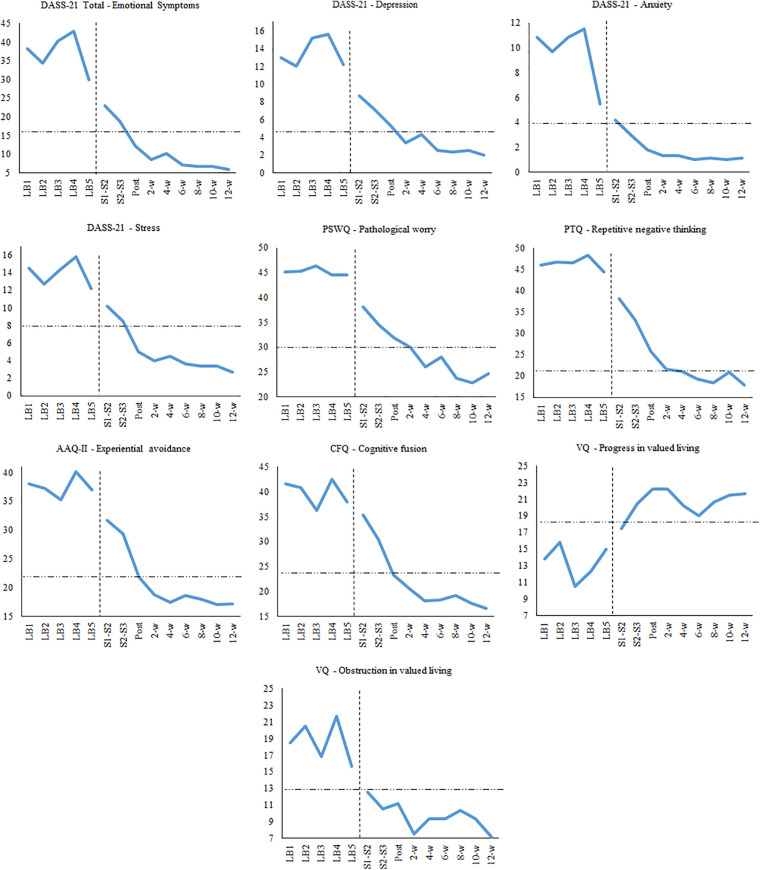
Mean scores on outcome and process measures across the study.

Figure 4 shows the mean results across participants in all the variables of the study. The mean scores for baselines were computed by averaging the scores of all participants in the first week, second week, third week, and so. Horizontal, dashed lines represent the mean scores of non-clinical samples in Colombia for each measure. At posttreatment, participants showed scores similar to the non-clinical samples in most of the measures. Most changes were observed at posttreatment, but participants continued improving during the 3-month follow-up. Overall, the changes in the outcomes and process measures took place simultaneously.

Table 3 shows the mean scores in all measures during baseline, posttreatment, and 6-, and 12-week follow-ups. Standardized mean differences for SCED were very large at posttreatment on the primary outcomes (DASS-Total: *d* = 2.16, DASS-Depression: *d* = 1.45, DASS-Anxiety: *d* = 1.47, DASS-Stress: *d* = 2.28, PSWQ: d = 2.69), but the effect sizes were higher at the 3-month follow-up (DASS-total: *d* = 3.34, DASS-Depression: *d* = 2.37, DASS-Anxiety: *d* = 1.90, DASS-Stress: *d* = 3.49, PSWQ: *d* = 4.52). Effect sizes were also very large for process measures at the 3-month follow-up (AAQ-II: *d* = 3.46; CFQ: *d* = 3.90; PTQ: *d* = 4.52; VQ-Progress: *d* = 0.92; VQ-Obstruction: *d* = 1.98).

**TABLE 3:** Means and standard deviations of each self-report measure at baseline, posttreatment, 6- and 12-week follow-ups, and effect sizes at posttreatment and 12-week follow-up.

	**Baseline**	**Post**	**6-week F-U**	**12-week F-U**	***d*-statistic for SCED Post treatment**		***d*-statistic for SCED 12-w F-U**	
	***M (SD)***	***M* (*SD)***	***M* (*SD)***	***M* (*SD)***	***d (SE)***	***95% CI***	***d (SE)***	***95% CI***
**Primary outcomes**								
DASS-Total: Emotional symptoms	37.17 (6.94)	12.17 (6.37)	7.17 (3.60)	5.83 (5.04)	2.16 (0.47)	[1.3, 3.1]	3.34 (0.76)	[2.0, 4.9]
DASS – Depression	13.60 (4.06)	5.33 (2.94)	2.50 (2.07)	2.00 (2.53)	1.45 (0.40)	[0.8, 2.3]	2.37 (0.78)	[1.1, 4.0]
DASS – Anxiety	9.67 (3.70)	1.83 (2.32)	1.00 (1.10)	1.17 (1.47)	1.47 (0.43)	[0.7, 2.3]	1.90 (0.62)	[0.9, 3.2]
DASS – Stress	13.90 (2.28)	5.00 (2.83)	3.67 (2.07)	2.67 (2.50)	2.28 (0.50)	[1.4, 3.3]	3.49 (0.74)	[2.2, 5.0]
PSWQ: Pathological worry	45.17 (3.49)	31.83 (8.33)	28.00 (8.56)	24.67 (7.47)	2.69 (0.95)	[1.0, 4.6]	4.52 (0.98)	[2.8, 6.6]
**Secondary outcomes (process measures)**								
AAQ-II: Experiential avoidance	37.57 (4.71)	21.83 (6.55)	18.67 (9.07)	17.17 (10.26)	2.06 (0.67)	[0.9, 3.4]	3.46 (0.80)	[2.1, 5.1]
CFQ: Cognitive fusion	39.87 (4.10)	23.33 (8.82)	18.33 (8.69)	16.67 (11.18)	2.32 (0.60)	[1.3, 3.6]	3.90 (0.91)	[2.3, 5.8]
PTQ: Perseverative thinking	46.37 (4.01)	25.67 (11.59)	19.33 (9.48)	17.83 (8.80)	3.27 (0.91)	[1.6, 5.1]	4.52 (0.90)	[3.0, 6.4]
VQ: Valued living – Progress	13.50 (7.03)	22.17 (3.82)	19.00 (6.81)	21.67 (4.13)	0.99 (0.33)	[0.5, 1.7]	0.92 (0.51)	[0.1, 0.9]
VQ: Valued living – Obstruction	18.63 (3.31)	11.17 (2.56)	9.33 (2.58)	7.17 (5.27)	1.44 (0.34)	[0.8, 2.1]	1.98 (0.49)	[1.1, 3.0]

*AAQ-II = Acceptance and Action Questionnaire – II; CFQ = Cognitive Fusion Questionnaire; DASS = Depression, Anxiety, and Stress Scales – 21; PSWQ = Penn State Worry Questionnaire; PTQ = Perseverative Thinking Questionnaire; SCED = Single-case experimental design; VQ = Valuing Questionnaire.*

## Discussion

The comorbidity between GAD and depression is very high, especially in primary care settings, and has been associated with greater chronicity, recurrence rates, health costs, and psychological disability (Hirschfeld, 2001; Wittchen, 2002; Zhu et al., 2009; Klenk et al., 2011). Unfortunately, the presence of this comorbidity predicts poorer therapeutic outcomes (Van Balkom et al., 2008; Newman et al., 2010; Coplan et al., 2015). Specifically, the empirical evidence shows that brief interventions tested for this problem in primary care have obtained small effect sizes (e.g. Cape et al., 2010). Accordingly, developing and testing brief psychological interventions for comorbid GAD and depression is crucial because of their potential to be adopted in primary care settings, where a shorter duration of treatment is the norm (Robinson and Reiter, 2016). A promising way to advance in this direction is to focus on the transdiagnostic processes involved in these disorders, which might improve the efficiency of interventions delivered in this setting.

In recent years, research has identified several transdiagnostic processes involved in depression and GAD, including RNT and EA (e.g. Hayes et al., 1999; Harvey A. G. et al., 2004; Wells, 2009; Barlow et al., 2011; Watkins, 2016). A recent functional contextual approach has linked RNT and experiential avoidance in a model of brief intervention called RNT-focused ACT (Ruiz et al., 2016a, 2018a, 2019; Dereix-Calonge et al., 2019; Salazar et al., 2020). The current study advances the evidence for the efficacy of brief RNT-focused ACT protocols in participants suffering from comorbid and severe GAD and depression. Six adults who met the criteria for both diagnoses received a 3-session RNT-focused ACT protocol. They showed stable levels of emotional symptoms and pathological worry during the 5 weeks of baseline.

To analyze clinically significant changes produced by the intervention, the Bayesian framework for SCED suggested by de Vries et al. (2016) was adopted. This framework is stricter than the most used method advocated by Jacobson and Truax (1991) and does not rely on the conceptual problems of *p*-values. Of the 6 participants, 5 showed CSC in both the DASS-21 and PSWQ. Most participants also showed CSC in RNT (5/6), experiential avoidance (4/6), and cognitive fusion (5/6). The standardized mean difference effect sizes for SCED were very large at the 12-week follow-up for outcome (DASS-total: *d* = 3.34; PSWQ: *d* = 4.52) and process measures (AAQ-II: *d* = 3.46; CFQ: *d* = 3.90; PTQ: *d* = 4.52). Importantly, these effect sizes share the same metric as the between-group Cohen’s *d*.

The results on values measures (i.e. VQ-Progress and VQ-Obstruction) were more modest according to the JZS + AR, which indicated that only 2 participants showed CSC in progress in values and 3 participants showed CSC in obstruction in values. The effect sizes for the values measures were also the lowest ones in this study (*d* = 0.92 and 1.98 for Progress and Obstruction, respectively). These results indicate that more explicit emphasis on values can be needed to obtain effect sizes similar to the other process measures.

The effect sizes obtained in this study were very large and improved from posttreatment to the 3-month follow-up. This finding is coherent with previous studies using RNT-focused ACT protocols (Ruiz et al., 2016a, 2018a, 2019). The augmentation of the intervention efficacy during the follow-up period might be due to the continuous practice and increased skill in disengaging from RNT. In this sense, it is not very likely to completely stop engaging in RNT after only a few sessions. Contrarily, this skill might be improved after some weeks of daily practice during which the individual begins to discriminate the triggers and the RNT process better and to disrupt the process while focusing on valued actions. However, the design of this study does not allow us to test this hypothesis. Further research should analyze the decrease of RNT as a mediator of the effect of the intervention effect.

Some limitations of this study are worth noting. The most important limitation is that dependent variables were measured exclusively through self-reports. Accordingly, further studies might analyze the intervention effect through independent clinician-administered assessments, ecological momentary assessment, or in-session participants’ behavior and verbalizations. Secondly, potential comorbidity with other emotional or personality disorders was not explored. Further studies might make use of a complete structured diagnostic interview. Thirdly, we used a delayed multiple-baseline design because of the difficulty of recruiting participants and for ethical reasons given the participants’ high level of emotional symptoms. This design does not allow the same level of experimental control as concurrent multiple-baseline designs (Harvey M. T. et al., 2004). However, some characteristics of this study make this limitation less relevant: (a) participants had been experiencing emotional symptoms for at least 12 months (i.e. symptoms were not momentary); (b) the 5 measurement points of baseline represent weekly measures, which indicates that the baseline showed no improvement trend across 1.5 months; and (c) maturation confounding effects are not a concern when considering adult behavior of different ages and with a very prolonged clinical problem (i.e. it is unlikely that biological or psychological processes would have had similar effects across the participants only due to the mere passage of time). Fourthly, a general limitation of SCEDs is that they lack active control conditions; thus, they do not control for the non-specific effects of therapy. Further studies should analyze the efficacy of the RNT-focused ACT protocol versus a psychological placebo. Fifthly, the design of this study did not permit us to analyze potential mediators of the effect of the intervention. In this sense, it is possible to analyze the processes of change at the individual level in SCED; however, a more intensive assessment should be conducted (e.g. Boswell et al., 2014). Lastly, only one therapist implemented the ACT protocol analyzed. Further studies should include several therapists to control for the effect of the therapist’s characteristics.

The effect sizes found in the current study were unusually large and contrasted with the weighted effect sizes found in a meta-analysis of the effect of cognitive behavior therapy (CBT) and ACT for major depression and GAD (e.g. Cuijpers et al., 2016; Bai et al., 2020). However, these findings should be taken with caution because, although the metric of the d-statistic computed in this study is the same as in group designs, the effect sizes found in SCED studies are usually higher than those found in group designs such as randomized controlled trials (Parker and Vannest, 2009; Shadish et al., 2016). Overall, this study indicates that RNT-focused ACT protocols are promising for the treatment of depression and GAD and warrant conducting randomized controlled trials to compare their effect versus waiting-list control conditions or brief versions of empirically established treatments such as behavioral activation or cognitive therapy.

## Conclusion

In conclusion, this study constitutes a promising step in the analysis of brief RNT-focused ACT protocols for the treatment of comorbid and severe GAD and depression. Further studies need to be conducted to establish the efficacy of this type of protocol and analyze its long-term effect. Importantly, if subsequent studies replicate the findings of this one, the RNT-focused ACT approach would be an excellent candidate to be adopted in mental health services.

## Data Availability Statement

All datasets generated for this study are included in the article/Supplementary Material.

## Ethics Statement

The studies involving human participants were reviewed and approved by the Comité de Bioética – Fundación Universitaria Konrad Lorenz. The patients/participants provided their written informed consent to participate in this study. Written informed consent was obtained from the individual(s) for the publication of any potentially identifiable images or data included in this article.

## Author Contributions

FR and CL contributed to the conception and design of the study. CF recruited the participants and collected and organized the database. FR implemented the interventions. JS-F and VC performed the statistical analysis. FR, CL, CF, JS-F, and VC-B prepared the manuscript.

## Conflict of Interest

The authors declare that the research was conducted in the absence of any commercial or financial relationships that could be construed as a potential conflict of interest.

## Footnotes

1
www.typeform.com

## Supplementary Material

FIGURE S1 Participants’ evolution in experiential avoidance across the study.

FIGURE S2 Participants’ evolution in cognitive fusion across the study.

FIGURE S3 Participants’ evolution in repetitive negative thinking across the study.

FIGURE S4 Participants’ evolution in progress in values across the study.

FIGURE S5 Participants’ evolution in obstruction in values across the study.

TABLE S1 Results in the JZS+AR analysis for each participant and measure with prior distributions of *r* = 0.5, *r* = 1.0, and *r* = 2.0.

DATA SHEET S1 Dataset of the study.

PRESENTATION S1 English version of the RNT-focused ACT protocol employed in the study.

PRESENTATION S2 Spanish version of the RNT-focused ACT protocol employed in the study.

## References

[B1] American Psychiatric Association (2000). *Diagnostic and Statistical Manual of Mental Disorders DSM-IV-TR*, 4th Edn. Washington, D.C: American Psychiatric Association.

[B2] Bai Z. Luo S. Zhang L. Wu S. Chi I. (2020). Acceptance and commitment therapy (ACT) to reduce depression: a systematic review and meta-analysis. *J. Affect. Disord.* 260 728–737. 10.1016/j.jad.2019.09.040 31563072

[B3] Barlow D. H. Farchione T. J. Fair5holme C. P. Ellard K. K. Boisseau C. L. Allen L. B. (2011). *Unified Protocol for Transdiagnostic Treatment of Emotional Disorders: Therapist guide.* New York, NY: Oxford University Press.

[B4] Barnes-Holmes D. Barnes-Holmes Y. Luciano C. McEnteggart C. (2017). From the IRAP and REC model to a multi-dimensional multi-level framework for analyzing the dynamics of arbitrarily applicable relational responding. *J. Context. Behav. Sci.* 6 434–445. 10.1016/j.jcbs.2017.08.001

[B5] Barnes-Holmes Y. Barnes-Holmes D. McHugh L. Hayes S. C. (2004). Relational frame theory: some implications for understanding and treating human psychopathology. *Int. J. Psychol. Psychol. Ther.* 4 355–375.

[B6] Bond F. W. Hayes S. C. Baer R. A. Carpenter K. M. Guenole N. Orcutt H. K. (2011). Preliminary psychometric properties of the acceptance and action questionnaire – II: a revised measure of psychological inflexibility and experiential avoidance. *Behav. Ther.* 42 676–688. 10.1016/j.beth.2011.03.007 22035996

[B7] Borkovec T. D. (1994). “The nature, functions and origins of worry,” in *Worrying: Perspectives on Theory, Assessment and Treatment*, eds Davey G. Tallis F., (Sussex: Wiley & Sons), 5–33.

[B8] Boswell J. F. Anderson L. M. Barlow D. H. (2014). An idiographic analysis of change processes in the unified transdiagnostic treatment of depression. *J. Consult. Clin. Psychol.* 82 1060–1071. 10.1037/a0037403 25045911

[B9] Boulanger J. L. Hayes S. C. Pistorello J. (2010). “Experiential avoidance as a functional contextual concept,” in *Emotion Regulation and Psychopathology: A Transdiagnostic Approach to Etiology and Treatment*, eds Kring A. Sloan D., (New York, NY: Guilford Press), 107–136.

[B10] Brown G. S. Jones E. R. (2005). Implementation of a feedback system in a managed care environment: what are patients teaching us? *J. Clin. Psychol.* 61 187–198. 10.1002/jclp.20110 15609353

[B11] Burcusa S. L. Iacono W. G. (2007). Risk for recurrence in depression. *Clin. Psychol. Rev.* 27 959–985. 10.1016/j.cpr.2007.02.005 17448579 PMC2169519

[B12] Cape J. Whittington C. Buszewicz M. Wallace P. Underwood L. (2010). Brief psychological therapies for anxiety and depression in primary care: meta-analysis and meta-regression. *BMC Med.* 8:38. 10.1186/1741-7015-8-38 20579335 PMC2908553

[B13] Cohen J. (1992). A power primer. *Psychol. Bull.* 112 155–159. 19565683 10.1037//0033-2909.112.1.155

[B14] Coplan J. D. Aaronson C. J. Panthangi V. Kim Y. (2015). Treating comorbid anxiety and depression: psychosocial and pharmacological approaches. *World J. Psychiatry* 5:366. 10.5498/wjp.v5.i4.366 26740928 PMC4694550

[B15] Côté S. Tremblay R. E. Nagin D. Zoccolillo M. Vitaro F. (2002). The development of impulsivity, fearfulness, and helpfulness during childhood: patterns of consistency and change in the trajectories of boys and girls. *J. Child Psychol. Psychol.* 4 609–618. 10.1111/1469-7610.00050 12120857

[B16] Criollo A. B. Díaz-Muelle S. Ruiz F. J. García-Martín M. B. (2018). Common physical properties improve metaphor effect even in the context of multiple examples. *Psychol. Rec.* 68 513–523. 10.1007/s40732-018-0297-9

[B17] Cuijpers P. Cristea I. A. Karyotaki E. Reijnders M. Huibers M. J. (2016). How effective are cognitive behavior therapies for major depression and anxiety disorders? A meta-analytic update of the evidence. *World Psychiatry* 15 245–258. 10.1002/wps.20346 27717254 PMC5032489

[B18] de Vries R. M. Meijer R. R. van Bruggen V. Morey R. D. (2016). Improving the analysis of routine outcome measurement data: what a Bayesian approach can do for you. *Int. J. Method Psychol. Res.* 25 155–167. 10.1002/mpr.1496 26449152 PMC6877109

[B19] de Vries R. M. Morey R. D. (2013). Bayesian hypothesis testing for single-subject designs. *Psychol. Methods* 18 165–185. 10.1037/a0031037 23458719

[B20] de Vries R. M. Morey R. D. (2015). A tutorial on computing Bayes factors for single-subject designs. *Behav. Ther.* 46 809–823. 10.1016/j.beth.2014.09.013 26520223

[B21] Dereix-Calonge I. Ruiz F. J. Sierra M. A. Peña-Vargas A. Ramírez E. S. (2019). Acceptance and commitment training focused on repetitive negative thinking for clinical psychology trainees: a randomized controlled trial. *J. Context. Behav. Sci.* 12 81–88. 10.1016/j.jcbs.2019.02.005

[B22] Ehring T. Watkins E. R. (2008). Repetitive negative thinking as a transdiagnostic process. *Int. J. Cognit. Ther.* 1 192–205. 10.1521/ijct.2008.1.3.192

[B23] Ehring T. Zetsche U. Weidacker K. Wahl K. Schönfeld S. Ehlers A. (2011). The perseverative thinking questionnaire (PTQ): validation of a content-independent measure of repetitive negative thinking. *J. Behav. Ther. Exp. Psychol.* 42 225–232. 10.1016/j.jbtep.2010.12.003 21315886 PMC3042595

[B24] Gelman A. Carlin J. B. Stern H. S. Dunson D. B. Vehtari A. Rubin D. B. (2014). *Bayesian Data Analysis*, Vol. 2. Boca Raton, FL: CRC Press.

[B25] Gil E. Luciano C. Ruiz F. J. Valdivia-Salas S. (2012). A preliminary demonstration of transformation of functions through hierarchical relations. *Int. J. Psychol. Psychol. Ther.* 12 1–20.

[B26] Gil E. Luciano C. Ruiz F. J. Valdivia-Salas S. (2014). Towards a functional analysis of hierarchical categorization. A further experimental example. *Int. J. Psychol. Psychol. Ther.* 14 137–153.

[B27] Gillanders D. T. Bolderston H. Bond F. W. Dempster M. Flaxman P. E. Campbell L. (2014). The development and initial validation of the cognitive fusion questionnaire. *Behav. Ther.* 45 83–101. 10.1016/j.beth.2013.09.001 24411117

[B28] Gil-Luciano B. (2018). *Analysis of the Hierarchical Responding in Psychological Flexibility.* doctoral thesis, University of Almería, Almería.

[B29] Gil-Luciano B. Calderón-Hurtado T. Tovar D. Sebastián B. Ruiz F. J. (2019). How are triggers for repetitive negative thinking organized? A relational frame analysis. *Psicothema* 31 53–59. 30664411 10.7334/psicothema2018.133

[B30] Gil-Luciano B. Ruiz F. J. Valdivia-Salas S. Suárez-Falcón J. C. (2017). Effect of framing behavior through deictic/hierarchical relations and specifying augmental functions in promoting psychological flexibility. *Psychol. Rec.* 67 1–9. 10.1007/s40732-016-0200-5

[B31] Glasgow R. E. Fisher L. Strycker L. A. Hessler D. Toobert D. J. King D. K. (2014). Minimal intervention needed for change: Definition, use, and value for improving health and health research. *Transl. Behav. Med.* 4 26–33. 10.1007/s13142-013-0232-1 24653774 PMC3958586

[B32] Harvey A. G. Watkins E. R. Mansell W. Shafran R. (2004). *Cognitive Behavioural Processes Across Disorders: A Transdiagnostic Approach to Research and Treatment.* Oxford: Oxford University Press.

[B33] Harvey M. T. May M. E. Kennedy C. H. (2004). Nonconcurrent multiple baseline designs and the evaluation of educational systems. *J. Behav. Educ.* 13 267–276. 10.1023/b:jobe.0000044735.51022.5d

[B34] Hayes S. C. (1981). Single case experimental design and empirical clinical practice. *J. Consult. Clin. Psychol.* 49 193–211. 10.1037/0022-006x.49.2.193 7217485

[B35] Hayes S. C. Barnes-Holmes D. Roche B. (2001). *Relational Frame Theory. A Post-Skinnerian Account of Human Language and Cognition.* New York, NY: Kluwer, Academic Press. 10.1016/s0065-2407(02)80063-5 11605362

[B36] Hayes S. C. Strosahl K. D. Wilson K. G. (1999). *Acceptance and Commitment Therapy. An Experiential Approach to Behavior Change.* New York, NY: Guilford Press.

[B37] Hayes S. C. Wilson K. G. Gifford E. V. Follette V. M. Strosahl K. D. (1996). Experiential avoidance and behavioral disorders: a functional dimensional approach to diagnosis and treatment. *J. Consult. Clin. Psychol.* 64 1152–1168. 10.1037/0022-006x.64.6.1152 8991302

[B38] Hedges L. V. Pustejovsky J. E. Shadish W. R. (2012). A standardized mean difference effect size for single case designs. *Res. Synth. Methods* 3 224–239. 10.1002/jrsm.1052 26062165

[B39] Hedges L. V. Pustejovsky J. E. Shadish W. R. (2013). A standardized mean difference effect size for multiple baseline designs across individuals. *Res. Synth. Methods* 4 324–341. 10.1002/jrsm.1086 26053946

[B40] Hilsenroth M. J. Handler L. Toman K. M. Padawer J. R. (1995). Rorschach and MMPI-2 indices of early psychotherapy termination. *J. Consult. Clin. Psychol.* 63 956–965. 10.1037/0022-006x.63.6.956 8543718

[B41] Hirschfeld R. M. A. (2001). The comorbidity of major depression and anxiety disorders: recognition and management in primary care. *Primary Care Compan. J. Clin. Psychol.* 3 244–254. 10.4088/pcc.v03n0609 15014592 PMC181193

[B42] Jacobson N. S. Truax P. (1991). Clinical significance: a statistical approach to defining meaningful change in psychotherapy research. *J. Consult. Clin. Psychol.* 59 12–19. 10.1037/0022-006x.59.1.12 2002127

[B43] Jeffreys H. (1961). *Theory of Probability.* Oxford: Oxford University Press.

[B44] Judd L. L. Akiskal H. S. Maser J. D. Zeller P. J. Endicott J. Coryell W. (1998). Major depressive disorder: a prospective study of residual subthreshold depressive symptoms as predictor of rapid relapse. *J. Affect. Disord.* 50 97–108. 10.1016/s0165-0327(98)00138-4 9858069

[B45] Kessler R. C. (2000). The epidemiology of pure and comorbid generalized anxiety disorder: a review and evaluation of recent research. *Acta Psychiatry Scand.* 102 7–13. 10.1111/j.0065-1591.2000.acp29-02.x 11131470

[B46] Kessler R. C. Berglund P. Demler O. Jin R. Koretz D. Merikangas K. R. (2003). The epidemiology of major depressive disorder: results from the national comorbidity survey replication (NCS-R). *J. Am. Med. Assoc.* 289 3095–3105. 10.1001/jama.289.23.3095 12813115

[B47] Kessler R. C. Bromet E. J. (2013). The epidemiology of depression across cultures. *Annu. Rev. Publ. Health* 34 119–138. 10.1146/annurev-publhealth-031912-114409 23514317 PMC4100461

[B48] Klenk M. M. Strauman T. J. Higgins E. T. (2011). Regulatory focus and anxiety: a self-regulatory model of GAD-depression comorbidity. *Pers. Individ. Differ.* 50 935–943. 10.1016/j.paid.2010.12.003 21516196 PMC3079259

[B49] Kratochwill T. R. Levin J. R. (2014). *Single-Case Intervention Research: Methodological and Statistical Advances.* Washington, DC: American Psychological Association.

[B50] Kroenke K. Spitzer R. L. Williams J. B. (2001). The PHQ-9. *J. Gen. Int. Med.* 16 606–613. 10.1046/j.1525-1497.2001.016009606.x PMC1495268 11556941

[B51] Kroenke K. Spitzer R. L. Williams J. B. Monahan P. O. Löwe B. (2007). Anxiety disorders in primary care: Prevalence, impairment, comorbidity, and detection. *Ann. Int. Med.* 146 317–325. 17339617 10.7326/0003-4819-146-5-200703060-00004

[B52] Kupfer D. J. Frank E. Wamhoff J. (1996). “Mood disorders: Update on prevention of recurrence,” in *Interpersonal Factors in the Origin and Course of Affective Disorders*, eds Mundt C. Goldstein M. J. Hahlweg K. Fiedler P., (London: Gaskell/Royal College of Psychiatrists), 289–302.

[B53] Lamers F. Va Oppen P. Comijs H. C. Smit J. H. Spinhoven P. Van Balkom A. J. (2011). Comorbidity patterns of anxiety and depressive disorders in a large cohort study: the Netherlands study of depression and ANXIETY (NESDA). *J. Clin. Psychiatry* 72 341–348. 10.4088/jcp.10m06176blu 21294994

[B54] López-López J. C. Luciano C. (2017). An experimental analysis of defusion interactions based on deictic and hierarchical framings and their impact on cognitive performance. *Psychol. Rec.* 67 485–497. 10.1007/s40732-017-0250-3

[B55] Lovibond P. F. Lovibond S. H. (1995). The structure of negative emotional states: comparison of the depression anxiety stress scales (DASS) with the beck depression and anxiety inventories. *Behav. Res. Ther.* 33 335–343. 10.1016/0005-7967(94)00075-u 7726811

[B56] Luciano C. (2017). The self and responding to the own’s behavior. Implications of coherence and hierarchical framing. *Int. J. Psychol. Psychol. Ther.* 17 267–275.

[B57] Luciano C. Ruiz F. J. Vizcaíno-Torres R. Sánchez V. Gutiérrez-Martínez O. López-López J. C. (2011). A relational frame analysis of defusion interactions in acceptance and commitment therapy. A preliminary and quasi-experimental study with at-risk adolescents. *Int. J. Psychol. Psychol. Ther.* 11 165–182.

[B58] Luciano C. Valdivia-Salas S. Ruiz F. J. (2012). “The self as the context for rule-governed behavior,” in *The Self and Perspective Taking: Research and Applications*, eds McHugh L. Stewart I., (Oakland, CA: Context Press), 143–160.

[B59] Meyer T. J. Miller M. L. Metzeger R. L. Borkovec T. D. (1990). Development and validation of the penn state worry questionnaire. *Behav. Res. Ther.* 28 487–495. 10.1016/0005-7967(90)90135-6 2076086

[B60] Newman M. G. Llera S. J. (2011). A novel theory of experiential avoidance in generalized anxiety disorder: a review and synthesis of research supporting a contrast avoidance model of worry. *Clin. Psychol. Rev.* 31 371–382. 10.1016/j.cpr.2011.01.008 21334285 PMC3073849

[B61] Newman M. G. Przeworski A. Fisher A. J. Borkovec T. D. (2010). Diagnostic comorbidity in adults with generalized anxiety disorder: impact of comorbidity on psychotherapy outcome and impact of psychotherapy on comorbid diagnoses. *Behav. Ther.* 41 59–72. 10.1016/j.beth.2008.12.005 20171328 PMC2827339

[B62] Nolen-Hoeksema S. (2004). “The response styles theory,” in *Depressive Rumination: Nature, Theory and Treatment*, eds Papageorgiou C. Wells A., (Chichester: Wiley), 107–123.

[B63] Nolen-Hoeksema S. Stice E. Wade E. Bohon C. (2007). Reciprocal relations between rumination and bulimia, substance abuse, and depressive symptoms in female adolescents. *J. Abnorm. Psychol.* 116 198–207. 10.1037/0021-843x.116.1.198 17324030

[B64] Olfson M. Mojtabai R. Sampson N. A. Hwang I. Druss B. Wang P. S. (2009). Dropout from outpatient mental health care in the United States. *Psychiatry Serv.* 60 898–907. 10.1176/ps.2009.60.7.898 PMC2774713 19564219

[B65] Parker R. I. Vannest K. J. (2009). An improved effect size for single-case research: nonoverlap of all pairs. *Behav. Ther.* 40 357–367. 10.1016/j.beth.2008.10.006 19892081

[B66] Parker R. I. Vannest K. J. (2012). Bottom-up analysis of single-case research designs. *J. Behav. Educ.* 21 254–265. 10.1007/s10864-012-9153-1

[B67] Phillips E. L. (2014). *Psychotherapy Revised: New Frontiers in Research and Practice.* Hillsdale, NJ: Routledge.

[B68] Pustejovsky J. E. (2016). *scdhlm: A Web-based Calculator for Between-case Standardized Mean Differences (Version 0.3.1) [Web application].* Available at: https://jepusto.shinyapps.io/scdhlm

[B69] Pustejovsky J. E. Hedges L. V. Shadish W. R. (2014). Design-comparable effect sizes in multiple baseline designs: a general modeling framework. *J. Educ. Behav. Stat.* 39 368–393. 10.3102/1076998614547577

[B70] Robinson P. J. Reiter J. T. (2016). *Behavioral Consultation and Primary Care. A Guide to Integrating Services*, 2nd Edn. New York, NY: Springer.

[B71] Roemer L. Orsillo S. M. (2002). Expanding our conceptualization of and treatment for generalized anxiety disorder: integrating mindfulness/acceptance-based approaches with existing cognitive-behavioral models. *Clin. Psychol. Sci. Pract.* 9 54–68. 10.1093/clipsy.9.1.54

[B72] Ruiz F. J. (2010). A review of acceptance and commitment therapy (ACT) empirical evidence: correlational, experimental psychopathology, component and outcome studies. *Int. J. Psychol. Psychol. Ther.* 10 125–162.

[B73] Ruiz F. J. (2019). Interfacing research on Clinical RFT and ACT: The case of RNT-focused ACT. Invited address at the 17th ACBS World Conference, Dublin.

[B74] Ruiz F. J. Flórez C. L. García-Martín M. B. Monroy-Cifuentes A. Barreto-Montero K. García-Beltrán D. M. (2018a). A multiple-baseline evaluation of a brief acceptance and commitment therapy protocol focused on repetitive negative thinking for moderate emotional disorders. *J. Context. Behav. Sci.* 9 1–14. 10.1016/j.jcbs.2018.04.004

[B75] Ruiz F. J. Monroy-Cifuentes A. Suárez-Falcón J. C. (2018b). Penn state worry questionnaire-11 validity in Colombian and factorial equivalence across gender and nonclinical and clinical samples. *An. Psicol.* 34 451–457. 10.6018/analesps.34.3.300281

[B76] Ruiz F. J. García-Beltrán D. M. Monroy-Cifuentes A. Suárez-Falcón J. C. (2019). Single-case experimental design evaluation of RNT-focused acceptance and commitment therapy in GAD with couple-related worry. *Int. J. Psychol. Psychol. Ther.* 19 261–276.

[B77] Ruiz F. J. García-Martín M. B. Suárez-Falcón J. C. Odriozola-González P. (2017a). The hierarchical factor structure of the depression anxiety and stress Scale-21. *Int. J. Psychol. Psychol. Ther.* 17 93–101.

[B78] Ruiz F. J. Suárez-Falcón J. C. Riaño-Hernández D. Gillanders D. (2017b). Psychometric properties of the cognitive fusion questionnaire in colombia. *Rev. Lat Am. Psicol.* 49 80–87. 10.1016/j.rlp.2016.09.006

[B79] Ruiz F. J. Riaño-Hernández D. Suárez-Falcón J. C. Luciano C. (2016a). Effect of a one-session ACT protocol in disrupting repetitive negative thinking: a randomized multiple-baseline design. *Int. J. Psychol. Psychol. Ther.* 16 213–233.

[B80] Ruiz F. J. Suárez-Falcón J. C. Cárdenas-Sierra S. Durán Y. Guerrero K. Riaño-Hernández D. (2016b). Psychometric properties of the acceptance and action questionnaire-II in Colombia. *Psychol. Rec.* 66 429–437. 10.1007/s40732-016-0183-2

[B81] Salazar D. M. Ruiz F. J. Ramírez E. S. Cardona-Betancourt V. (2020). Acceptance and commitment therapy focused on repetitive negative thinking for child depression: a randomized multiple-baseline evaluation. *Psychol. Rec.* (in press). 10.3389/fpsyg.2020.00356 PMC7082425 32231614

[B82] Saxena S. Thornicroft G. Knapp M. Whiteford H. (2007). Resources for mental health: scarcity, inequity, and inefficiency. *Lancet* 370 878–889. 10.1016/s0140-6736(07)61239-2 17804062

[B83] Sen P. K. (1968). Estimates of the regression coefficient based on Kendall’s tau. *J. Am. Stat. Assoc.* 63 1379–1389. 10.1080/01621459.1968.10480934

[B84] Shadish W. R. Zelinsky N. A. Vevea J. L. Kratochwill T. R. (2016). A survey of publication practices of single-case design researchers when treatments have small or large effects. *J. Appl. Behav. Anal.* 49 656–673. 10.1002/jaba.308 27174301

[B85] Sheehan D. V. Lecrubier Y. Sheehan K. H. Amorim P. Janavs J. Weiller E. (1998). The mini-international neuropsychiatric interview (M.I.N.I.): the development and validation of a structured diagnostic psychiatric interview for DSM-IV and ICD-10. *J. Clin. Psychiatry* 59 22–33. 9881538

[B86] Sierra M. A. Ruiz F. J. Flórez C. L. Riaño-Hernández D. Luciano C. (2016). The role of common physical properties and augmental functions in metaphor effect. *Int. J. Psychol. Psychol. Ther.* 16 265–279.

[B87] Smout M. Davies M. Burns N. Christie A. (2014). Development of the valuing questionnaire (VQ). *J. Context. Behav. Sci.* 3 164–172. 10.1016/j.jcbs.2014.06.001

[B88] Spitzer R. L. Kroenke K. Williams J. B. Löwe B. (2006). A brief measure for assessing generalized anxiety disorder: The GAD-7. *Arch. Int. Med.* 166 1092–1097. 16717171 10.1001/archinte.166.10.1092

[B89] Stark M. M. (1992). Dropping out of substance abuse treatment: A clinically oriented review. *Clin. Psychol. Rev.* 12 93–116. 10.1016/0272-7358(92)90092-m

[B90] Stiles W. B. Barkham M. Connell J. Mellor-Clark J. (2008). Responsive regulation of treatment duration in routine practice in United Kingdom primary care settings: replication in a larger sample. *J. Consult. Clin. Psychol.* 76:298. 10.1037/0022-006x.76.2.298 18377125

[B91] Strosahl K. D. Robinson P. J. Gustavsson T. (2012). *Brief Interventions for Radical Change: Principles and Practice of Focused Acceptance and Commitment Therapy.* Oakland, CA: New Harbinger.

[B92] Tate R. L. Perdices M. Rosenkoetter U. McDonald S. Togher L. Shadish W. (2016). The single-case reporting guideline in behavioural interventions (SCRIBE) 2016: explanation and elaboration. *Arch. Sci. Psychol.* 4 10–31. 10.1037/arc0000027

[B93] Törneke N. (2017). *Metaphor in Practice: A Professional’s Guide to Using the Science of Language in Psychotherapy.* Oakland, CA: New Harbinger Publications.

[B94] Törneke N. Luciano C. Barnes-Holmes Y. Bond F. W. (2016). “Relational frame theory and three core strategies in understanding and treating human suffering,” in *The Wiley Handbook of Contextual Behavioral Science*, eds Zettle R. D. Hayes S. C. Barnes-Holmes D. Biglan A., (New York, NY: Wiley-Blackwell), 254–272. 10.1002/9781118489857.ch12

[B95] Valentine J. C. Tanner-Smith E. E. Pustejovsky J. E. (2016). *Between-case Standardized Mean Difference Effect Sizes for Single-Case Designs: A Primer and Tutorial Using the scdhlm Web Application.* Oslo: The Campbell Collaboration.

[B96] Van Balkom A. J. Van Boeijen C. A. Boeke A. J. P. Van Oppen P. Kempe P. T. Van Dyck R. (2008). Comorbid depression, but not comorbid anxiety disorders, predicts poor outcome in anxiety disorders. *Depress. Anxiety* 25 408–415. 10.1002/da.20386 17960642

[B97] Vannest K. J. Parker R. I. Davis J. L. Soares D. A. Smith S. L. (2012). The theil-sen slope for high stakes decision from progress monitoring. *Behav. Disord.* 37 271–280. 10.1177/019874291203700406

[B98] Vannest K. J. Parker R. I. Gonen O. (2011). *Single-Case Research: Web-Based Calculator for SCR Analysis, Version 1.0 (Web-based Application).* College Station, TX: Texas A&M University.

[B99] Villatte M. Villatte J. L. Hayes S. C. (2015). *Mastering the Clinical Conversation: Language as Intervention.* New York, NY: Guilford Press.

[B100] Watkins E. R. (2016). *Rumination-Focused Cognitive-Behavioral Therapy for Depression.* New York, NY: Guilford Press.

[B101] Wells A. (2009). *Metacognitive Therapy for Anxiety and Depression.* New York, NY: Guilford Press.

[B102] Wilson K. G. Luciano C. (2002). *Terapia de Aceptación y Compromiso. Un Tratamiento Conductual Orientado a los Valores [Acceptance and Commitment Therapy. A Values-Oriented Behavioral Treatment].* Madrid: Pirámide.

[B103] Wittchen H. U. (2002). Generalized anxiety disorder: prevalence, burden, and cost to society. *Depress. Anxiety* 16 162–171. 10.1002/da.10065 12497648

[B104] Wittchen H. U. Hoyer J. (2001). Generalized anxiety disorder: nature and course. *J. Clin. Psychol.* 62 15–21. 11414546

[B105] World Health Organization [WHO] (2017). *Depression and Other Common Mental Disorders: Global Health Estimates.* Geneva: WHO.

[B106] Zhu B. Zhao Z. Ye W. Marciniak M. D. Swindle R. (2009). The cost of comorbid depression and pain for individuals diagnosed with generalized anxiety disorder. *J. Nerv. Ment. Dis.* 197 136–139. 10.1097/nmd.0b013e3181963486 19214050

