# Corrigendum: A multiple-baseline evaluation of acceptance and commitment therapy focused on repetitive negative thinking for comorbid generalized anxiety disorder and depression

**DOI:** 10.3389/fpsyg.2023.1258820

**Published:** 2023-08-02

**Authors:** Francisco J. Ruiz, Carmen Luciano, Cindy L. Flórez, Juan C. Suárez-Falcón, Verónica Cardona-Betancourt

**Affiliations:** ^1^Faculty of Psychology, Fundación Universitaria Konrad Lorenz, Bogotá, Colombia; ^2^Departament of Psychology, Universidad de Almería, Almería, Spain; ^3^Madrid Institute of Contextual Psychology, Madrid, Spain; ^4^Department of Behavioral Sciences Methodology, Universidad Nacional de Educación a Distancia, Madrid, Spain; ^5^Universitat Oberta de Catalunya, Barcelona, Spain

**Keywords:** acceptance and commitment therapy, relational frame theory, depression, generalized anxiety disorder, repetitive negative thinking

In the published article, the Funding statement was omitted in error. The correct Funding statement appears below.

