# Supplementary figures and images for: A Multiple-Baseline Evaluation of Acceptance and Commitment Therapy Focused on Repetitive Negative Thinking for Comorbid Generalized Anxiety Disorder and Depression
Source: Front Psychol. 2020 Mar 13;11:356. doi: 10.3389/fpsyg.2020.00356 (PMC7082425; doi:10.3389/fpsyg.2020.00356)

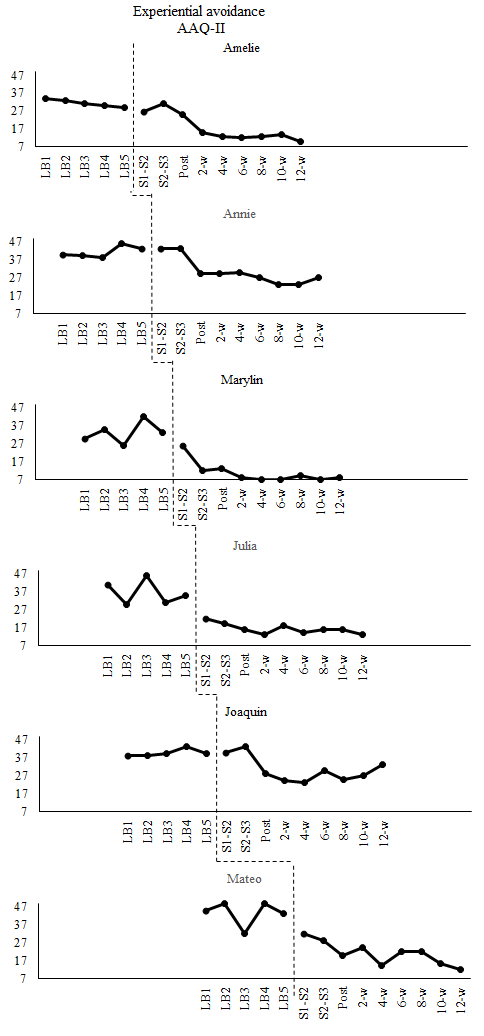

Supplement: FIGURE S1 — Participants’ evolution in experiential avoidance across the study. [file Image_1.tif]

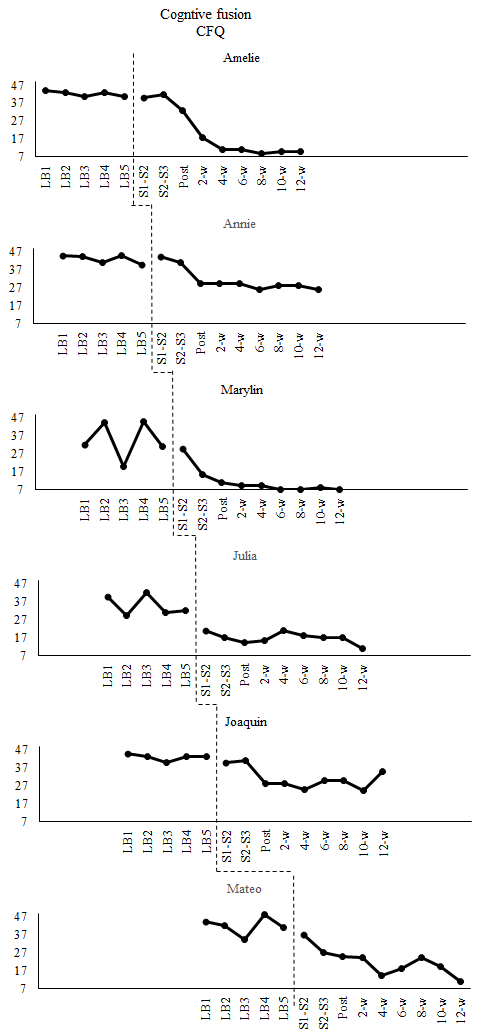

Supplement: FIGURE S2 — Participants’ evolution in cognitive fusion across the study. [file Image_2.tif]

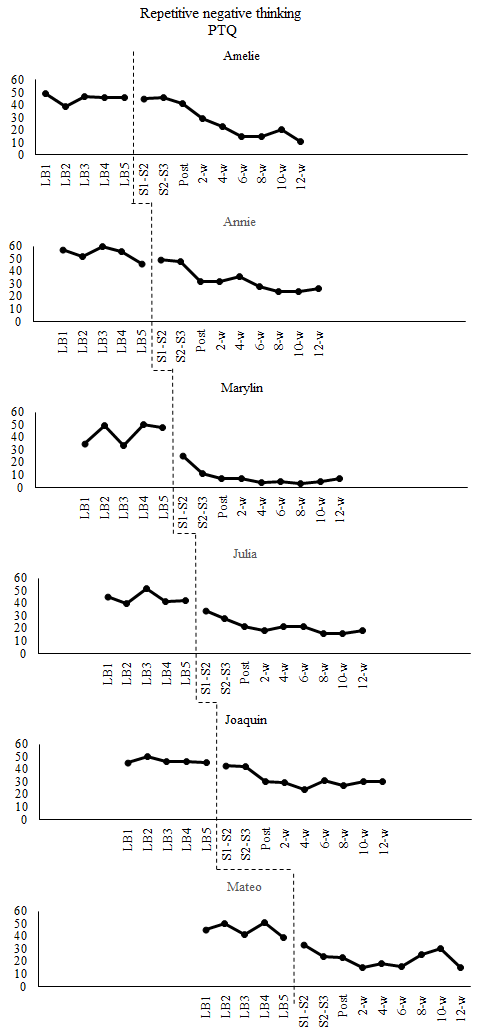

Supplement: FIGURE S3 — Participants’ evolution in repetitive negative thinking across the study. [file Image_3.tif]

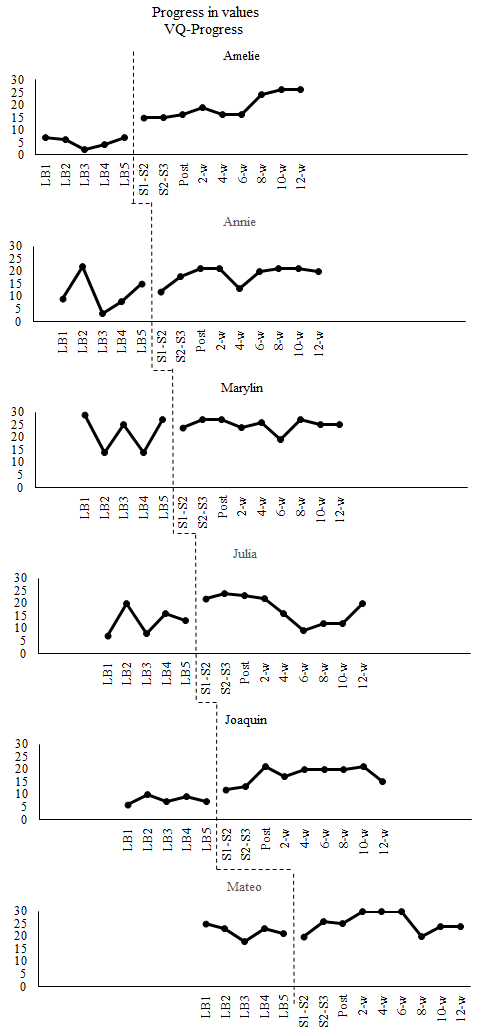

Supplement: FIGURE S4 — Participants’ evolution in progress in values across the study. [file Image_4.tif]

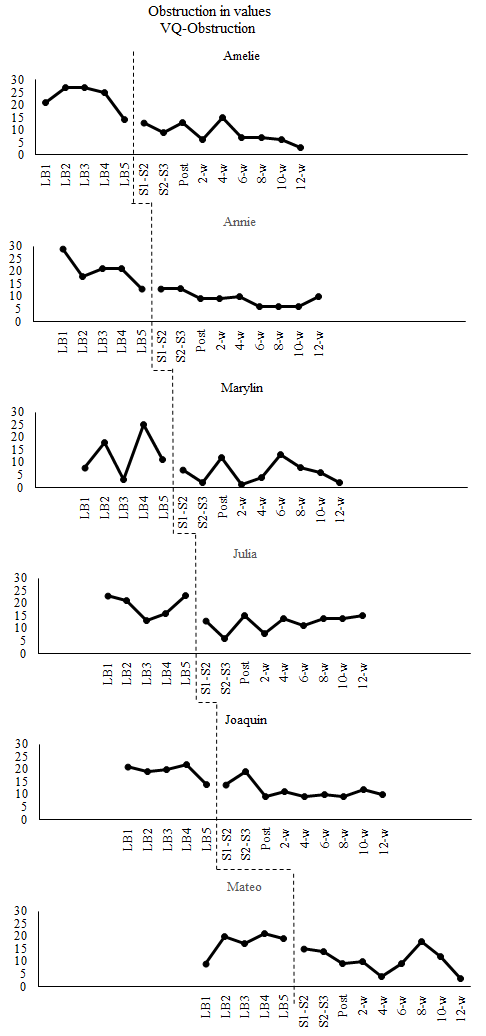

Supplement: FIGURE S5 — Participants’ evolution in obstruction in values across the study. [file Image_5.tif]
