# Supplementary material for: A Multiple-Baseline Evaluation of Acceptance and Commitment Therapy Focused on Repetitive Negative Thinking for Comorbid Generalized Anxiety Disorder and Depression
Source: Front Psychol. 2020 Mar 13;11:356. doi: 10.3389/fpsyg.2020.00356 (PMC7082425; doi:10.3389/fpsyg.2020.00356)
Supplement: TABLE S1 — Results in the JZS+AR analysis for each participant and measure with prior distributions of r = 0.5, r = 1.0, and r = 2.0. [file Table_1.DOCX]

Appendix A. Results in the JZS+AR Analysis for each Participant and Measure with Prior Distributions of *r* = 0.5, *r* = 1.0, and *r* = 2.0

|  |  |  | P1 | P2 | P3 | P4 | P5 | P6 |
| --- | --- | --- | --- | --- | --- | --- | --- | --- |
| PRIMARY OUTCOMES | | | | | | | | |
| DASS – Total | *r* = 0.5 | *δ* | 8.5 | 2.1 | 0.7 | 0.9 | 3.0 | 6.5 |
|  |  | *B_ar_* | **>100** | **8.4** | 2.4 | 2.8 | **19.8** | **>100** |
|  | *r* = 1 | *δ* | 8.6 | 2.6 | 1.0 | 1.3 | 3.3 | 6.6 |
|  |  | *B_ar_* | **>100** | **14.5** | 2.1 | **3.2** | **31.7** | **>100** |
|  | *r* = 2 | *δ* | 8.8 | 2.9 | 1.2 | 1.5 | 3.6 | 6.8 |
|  |  | *B_ar_* | **>100** | **18.1** | 1.8 | **3.0** | **49.9** | **>100** |
| PSWQ  (Pathological worry) | *r* = 0.5 | *δ* | 11.0 | 7.5 | 7.2 | 2.4 | 3.7 | 3.9 |
|  |  | *B_ar_* | **>100** | **>100** | **>100** | **11.7** | **39.3** | **33.0** |
|  | *r* = 1 | *δ* | 11.0 | 7.6 | 7.2 | 2.7 | 4.0 | 4.2 |
|  |  | *B_ar_* | **>100** | **>100** | **>100** | **17.8** | **73.5** | **73.1** |
|  | *r* = 2 | *δ* | 11.1 | 7.8 | 7.5 | 3.0 | 4.3 | 4.4 |
|  |  | *B_ar_* | **>100** | **>100** | **>100** | **26.6** | **97.2** | **>100** |
| SECONDARY OUTCOMES | | | | | | | | |
| AAQ-II  (Experiential avoidance) | *r* = 0.5 | *δ* | 7.6 | 4.2 | 3.5 | 2.3 | 2.5 | 2.7 |
|  |  | *B_ar_* | **>100** | **47.2** | **30.1** | **11.3** | **12.2** | **14.9** |
|  | *r* = 1 | *δ* | 7.7 | 4.5 | 3.7 | 2.6 | 2.9 | 3.0 |
|  |  | *B_ar_* | **>100** | **80.1** | **44.5** | **16.2** | **23.5** | **24.9** |
|  | *r* = 2 | *δ* | 7.9 | 4.8 | 4.0 | 3.0 | 3.2 | 3.3 |
|  |  | *B_ar_* | **>100** | **>100** | **62.6** | **23.7** | **30.2** | **32.1** |
| CFQ  (Cognitive fusion) | *r* = 0.5 | *δ* | 24.1 | 5.7 | 1.8 | 2.3 | 2.2 | 2.7 |
|  |  | *B_ar_* | **>100** | **>100** | **6.8** | **11.1** | **10.7** | **14.6** |
|  | *r* = 1 | *δ* | 23.9 | 5.9 | 2.1 | 2.7 | 2.6 | 3.0 |
|  |  | *B_ar_* | **>100** | **>100** | **9.5** | **16.7** | **15.5** | **22.3** |
|  | *r* = 2 | *δ* | 24.1 | 6.0 | 2.5 | 3.0 | 3.0 | 3.3 |
|  |  | *B_ar_* | **>100** | **>100** | **11.8** | **23.2** | **22.8** | **32.7** |
| PTQ  (Perseverative thinking) | *r* = 0.5 | *δ* | 5.7 | 4.8 | 4.0 | 5.0 | 7.0 | 2.0 |
|  |  | *B_ar_* | **>100** | **59.6** | **53.2** | **76.6** | **>100** | **8.1** |
|  | *r* = 1 | *δ* | 5.8 | 5.1 | 4.2 | 5.2 | 7.1 | 2.4 |
|  |  | *B_ar_* | **>100** | **>100** | **80.6** | **>100** | **>100** | **12.3** |
|  | *r* = 2 | *δ* | 6.1 | 5.2 | 4.5 | 5.3 | 7.3 | 2.7 |
|  |  | *B_ar_* | **>100** | **>100** | **>100** | **>100** | **>100** | **17.4** |
| VQ (Progress values) | *r* = 0.5 | *δ* | 8.4 | 0.7 | 0.2 | 0.2 | 3.3 | 0.08 |
|  |  | *B_ar_* | **>100** | 2.0 | 1.0 | 0.8 | **26.9** | 0.7 |
|  | *r* = 1 | *δ* | 8.6 | 0.9 | 0.3 | 0.2 | 3.5 | 0.1 |
|  |  | *B_ar_* | **>100** | 2.0 | 0.8 | 0.6 | **39.6** | 0.5 |
|  | *r* = 2 | *δ* | 8.7 | 1.2 | 0.5 | 0.3 | 3.9 | 0.1 |
|  |  | *B_ar_* | **>100** | 1.7 | 0.5 | 0.4 | **65.1** | 0.3 |
| VQ (Obstruction values) | *r* = 0.5 | *δ* | 2.1 | 1.3 | 0.4 | 0.7 | 1.8 | 0.4 |
|  |  | *B_ar_* | **7.2** | **4.2** | 1.3 | 2.0 | **7.0** | 1.3 |
|  | *r* = 1 | *δ* | 2.5 | 1.7 | 0.6 | 1.0 | 2.2 | 0.6 |
|  |  | *B_ar_* | **11.5** | **5.6** | 1.2 | 2.0 | **9.8** | 1.2 |
|  | *r* = 2 | *δ* | 2.8 | 2.1 | 0.8 | 1.3 | 2.5 | 0.8 |
|  |  | *B_ar_* | **14.4** | **6.2** | 0.9 | 1.8 | **11.6** | 0.9 |
