# Supplementary material for: A Multiple-Baseline Evaluation of Acceptance and Commitment Therapy Focused on Repetitive Negative Thinking for Comorbid Generalized Anxiety Disorder and Depression
Source: Front Psychol. 2020 Mar 13;11:356. doi: 10.3389/fpsyg.2020.00356 (PMC7082425; doi:10.3389/fpsyg.2020.00356)
Supplement: PRESENTATION S1 — English version of the RNT-focused ACT protocol employed in the study. [file Presentation_1.pdf]

THREE-SESSION ACT PROTOCOL FOCUSED ON DISRUPTING REPETITIVE NEGATIVE THINKING PATTERNS FOR COMORBID DEPRESSION AND GENERALIZED ANXIETY DISORDER

SESSION 1

1. Establishing the context for a brief intervention.

“The work that I propose for you to perform is based on a model of brief intervention that aims to achieve major changes as soon as possible. As you may know if you have previously gone through a therapeutic process, the psychological interventions sometimes proceed slowly and require a large amount of information during the assessment. In our case, when suggesting a brief intervention, we are going to try to go straight to the point, to the heart of the problem that you are experiencing. This has the advantage that, if the work we do is useful, we will surely notice it quickly. However, when going directly to the point, there may be times when you feel that we are going very fast and that the session may be intense. If, at any time, you think we should go a little more slowly or you feel that something important has been left out, feel free to let me know. Even though I propose a brief intervention, you will always set the pace of the work, and whatever we do will be aimed at making the change you want in your life. In any case, I'd like to let you know that, although you're going through some difficult times that have brought you here, I think you're a complete, responsible, and valuable person, who is capable of doing work that goes directly to the point so that it will be useful as soon as possible. Do you think this is a good work plan?”

2. Presentation of the logic of the intervention.

“First of all, allow me to comment on the logic of the intervention and the work we are going to do. This intervention is designed in accordance with recent research that grants worry and rumination (turning things over excessively in one’s mind, entanglement, etc.) a crucial role in the development and maintenance of emotional problems in people’s lives, making them feel more anxious and sad and failing to fulfill their life goals”.

“With this in mind, the work I propose we do during these sessions is to develop the ability to detect when we are fruitlessly entangled in our minds and to be able to let this be and focus on what is really important in our lives”.

“First of all, let me explain for a moment why we sometimes get entangled, turning things over in our minds, etc. It turns out that everyone has had lots of thoughts and feelings throughout their lives; we are going to have them today and we will continue to have thousands and thousands for the rest of our lives. For example, surely you can remember for a minute what thoughts came to your mind when you were 8 years old... when you were 15 years old... the ones you had a year ago... the ones you had yesterday... the ones you're having right now... Throughout life, we have surely been observing how thoughts come and go while the only constant is us, that is, the person, PEPE (patient's name). However, sometimes, we get a thought or fear related to something that we do not want to happen or a thought related to the need to explain something important and, although

those thoughts are ours—just a momentary thing that we could let go of like the rest of the thoughts—, as they are very annoying, we forget that they are only thoughts and that we can choose how much importance we grant them... Whether we let them be and go on to something else—because all thoughts are in the end momentary—or we entangle with them and grant them power over our life during that time, which sometimes turns into hours, then days, months, and even years. And then we entangle thinking about them, we place all our attention on them and we think and think, we worry and we mull over them, over and over again... Many times, that mulling over is very repetitive, but we keep on doing it over and over again. And over and over again... Often, we do not even feel that it is really us who are choosing to entangle with them. We simply do it automatically, without realizing that, at any time, we could choose to let go of our fear and concern. We completely forget that we are facing thoughts that are ours and we end up creating alternative realities in our minds…"

"As I said earlier, what we are going to try to do is for PEPE to learn to realize how, in the face of certain thoughts of his, he chooses, often without knowing it, to get entangled and entangled with them... As if, in the face of the flow of thoughts, he placed a dam or a reservoir in front of some of them so that they get stuck and PEPE begins to look at them and analyze them over and over again until they start to spin around like a whirlpool... And we often forget that we are really the ones who put up the dam and we form the whirlpool of thoughts... without realizing that we can choose to open the dam, let them go through and focus our attention on what is important to us…"

"In view of what we have commented on previously, during this session, you may notice something a little strange: sometimes we will stop so you can see how PEPE is having certain thoughts, and you will notice at each step what you're choosing to do: whether you close the dam or open it and let those thoughts pass through so we can focus on what is important in PEPE's life. For example, allow yourself for a moment to observe the thoughts or feelings that come to your mind at this moment without doing anything with them. To help us, we will use these pieces of paper. Whatever it is, it's OK... Simply write it on the paper and look at it as if it were a painting or a landscape: without doing anything with it, simply observing it... Notice how PEPE is choosing to contemplate it and let it be. And now open the dam and let it go through... Who just chose to let it go?"

"Let another thought come to you... The only thing that you should do at this moment is to choose to write it down and to contemplate it on the paper... Realize again that it is a thought of yours and that you can choose whether to open the dam or get entangled in mulling over this thought..." This is repeated with three more thoughts.

3. **Presentation of the work outline and establishment of worry/rumination as the focus of assessment and intervention.**

"Throughout the intervention, I propose to work with this outline. Here, we are going to put your name, the name of the person who has all thoughts, memories and feelings… In general, everyone can perform actions during the day that go in two directions. On the one hand, we can do things that go in the direction that brings us closer to what is important for us and enriches our life, the kind of things that make us feel proud, that make us think that the day was worthwhile when we go to sleep and we sleep with a smile of satisfaction on our face, the kind of actions that we will feel proud to remember having done for a long time, even when we are old. Allow yourself to notice the thoughts that come to your mind without doing anything with them. Just notice what they are… Write down in this piece of paper or imagine that you write them down… And note that you can choose to follow paying attention to me."

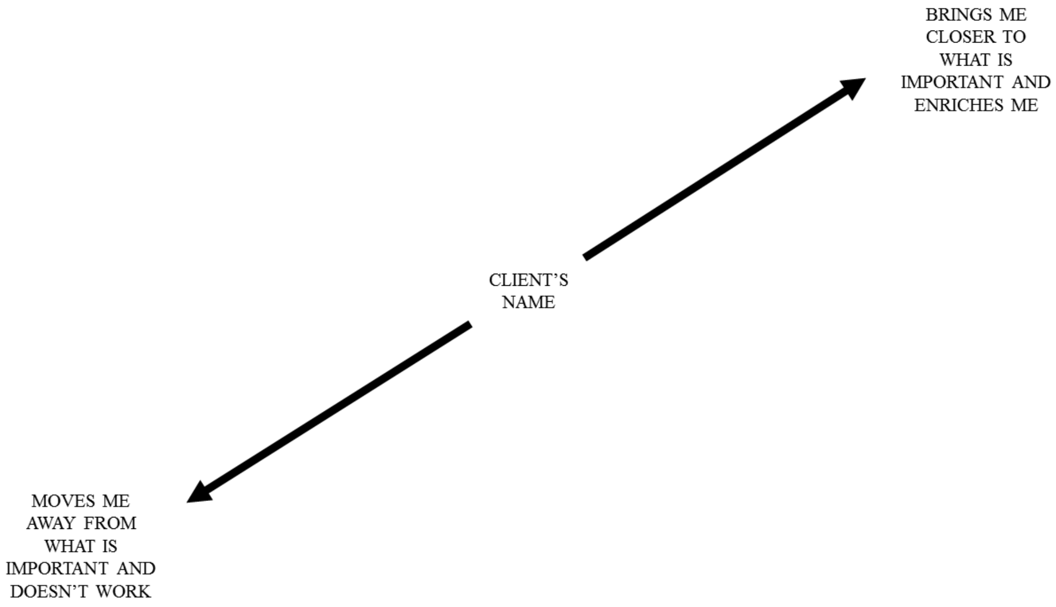

On the other hand, we can do things that move us away from what is important to us or keep us stuck without moving towards what is important and enriching. They are the kind of things that we would not feel especially proud of at the end of the day or with the passing of years. Again, allow yourself to simply note what thought goes through your mind without doing anything with it, just note yourself being the person who is observing it. Virtually all people perform actions in both directions throughout the day, some people do more pointing up and to the right and others do more pointing down and to the left.

Usually, people seeking psychological intervention are doing more down-left actions than up-right ones, and that is why they feel dissatisfied with their lives. The work that I propose for you to do here is to see what you do in your day-to-day life that takes you in one or the other direction, so that you will do increasingly more things that bring you closer to what is important to you and enrich your life, and increasingly fewer things that distance you from what is important and that have entangled you. Does this make sense to you? If we achieve this, could we say that the work would have been successful?"

"In this session, I propose to review how much time you spend doing things that move you away from what is important or, at least, do not allow you to go in that direction and that keep you entangled.

Many times, the things that are beginning to move us away from what is important or that keep us stuck have to do with spending a lot of time entangled in our own thoughts, worries, memories, etc. That is, mulling things over, arguing with ourselves, thinking about why something works that way, and so on, over and over, etc. Mulling things over is not necessarily a bad thing. For example, there are times when we worry about the future and thus anticipate possible problems and find solutions. Sometimes we think about something that has happened and discover why it occurred and that serves us for the present and the future. However, often, thinking too much or mulling things over is not useful, and in spite of that, we go on ceaselessly, losing contact with the important things that are happening or that we could do at that time. In other words, we are going towards the left, away from what is important for us."

4. **Identification of the hierarchy of worry/rumination triggers and the chain of main thoughts.**

The therapist now explores what private event is at the top of the hierarchy of the worry/rumination triggers. Therefore, it is important for the therapist to ask particularly through metaphors to facilitate the client's responding with the top of the hierarchy (the "Daddy" of fears or things that should be explained). One way to ask is as follows:

"At this time, I invite you to think for a few minutes about when you get entangled in your mind. Often, the trigger of getting entangled with our thoughts is the fear that something will happen or the need to find out why certain things happened. Imagine that you have a magic gun that you can shoot at the thoughts that entangle you, but you only have one bullet. Which thought would you shoot? Which one could you shoot so you could kill more than "one bird" with one shot? What thought could you shoot that is the daddy of your fears, the daddy that nourishes and sustains the rest of thoughts? What thought would make the other thoughts fall like a house of cards after you shot it?"

"Allow yourself to notice for a moment who has just answered and whose thought that is which often acts as the boss... Just watch and see how you can choose to look at it written in the outline without doing anything with it... During this time, I would like to talk to PEPE, not to that thought that sometimes gains a lot of strength in PEPE's life ... what do you choose to do? Do we let the thought—Pepe's boss—speak or do you choose to speak yourself? Surely, the boss is there for some reason; it appears for some reason, but allow PEPE to be the one speaking... Because nobody knows better than PEPE about PEPE's life... Not even his boss…"

"Now I invite you to talk for a while about what the boss that often comes to PEPE is made of...."

At this point, the client responds by indicating a thought that may or may not be at the top of the hierarchy of triggers. Next, cues are presented to intuit whether or not the client identified the top, and questions are made to further develop the functional analysis. The goal of the therapist at this point is to identify the top trigger ("Daddy") and triggers it contains ("kids"), identifying with the client how his/her process of worry/rumination usually proceeds.

- **Cues that indicate that the private event pointed out by the client is not the top of the hierarchy**:
  - ❑ It is a particular fear with an "expiration date" (i.e., fear of failing an exam next week, fear of acting silly at a public event, a romantic break-up, failing in something concrete, etc.).
  - ❑ The private event does not seem to be overly aversive.
  - ❑ The private event has appeared relatively recently in the client's life, but their emotional problems stem from further back.

- **Useful questions when we suspect that the private event isn't at the top of the hierarchy**:
  - ❑ Example 1: the client says he/she is afraid of not passing a subject.

Is it something that happens to you often? If the client says "Yes," it is likely to form part of something more general.

Does it usually happen in areas other than your studies? Are there more things in your life that you afraid of "failing"? If the client indicates other areas, it is likely that the "daddy" is fear of failing, or "I am a failure."

What comes to mind when you think you're going to fail the subject? If the client indicates thoughts related to the subject (e.g., "I'll have to study it all over again"), this would indicate that it is possible that there is nothing "bigger." However, if he/she responds with something more generic (e.g., "I am not going to be a good professional," "I doubt if I

am capable”), this would indicate that there is probably something more encompassing, such as the fear not being a good professional or “I'm not smart.”
Why would it be so bad for you to fail the subject?
Do you think that there is a bigger fear or thought that feeds this fear? Does that fear have a daddy that generated it?

- Example 2: The client says that he/she is afraid his/her partner will leave him/her.

Has this happened with other partners? If so, maybe it is fear of not having a partner.
Does it also happen with other people even if they are not your partner? If so, maybe it is fear of being alone or not having protection.
Why would it be so bad for your partner to leave you?
If your partner left you, would that prove that, deep inside, there is something wrong with you?
What would be the worst thought that you might have about yourself if your partner left you?

- **Cues indicate that the private event indicated by client is at the top of the hierarchy**:

- ❑ It is abstract, and more concrete thoughts derive from it.
- ❑ It is present in several areas of the life of the client.
- ❑ It is “old.”
- ❑ It is extremely distressing for the client.
- ❑ It is the thought that client would have if his/her life did not go as he wished.

- **Useful questions when we suspect that the private event is at the top of the hierarchy**:

- ❑ In how many areas of your life is this thought present and causes distress?
- ❑ Would this be the thought that would hurt you the most if it were true?
- ❑ How old is this thought? Does it have “kids”? Has it been reproduced in more concrete fears?
- ❑ If we could “remove” it, would the rest of the distressing thoughts make sense or disappear?

Throughout the evaluation, the therapist tries to draw an outline similar to the one presented in the following image. It is important to identify the top of the hierarchy and some of the most representative thoughts through which the worry/rumination process passes. Usually, clients are not especially skilled at discriminating the worry/rumination process, so it is very useful to try to specify how it happens.

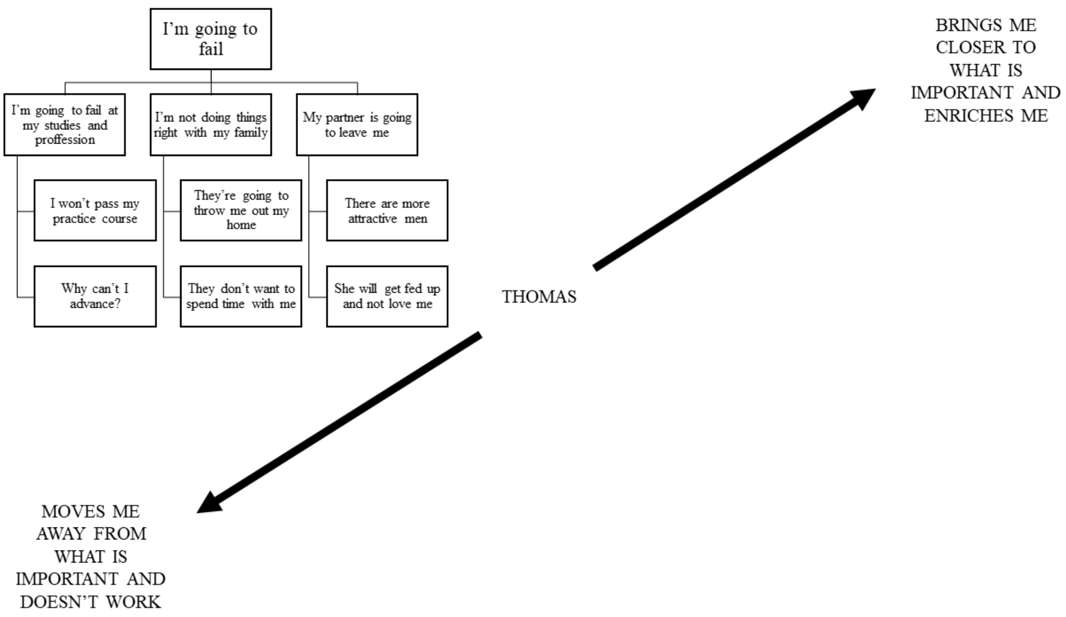

During the previous process, when PEPE specifies other "bosses" or "little bosses", we ask him to notice for a moment how what he just said is another thought and he can choose at that time to contemplate it, let it be and remain focused on the conversation or he can choose to entangle with that "little boss", and we can spend the session doing the same thing that happens every day in PEPE's life.

5. **Analysis of other experiential avoidance strategies that the worry/rumination pattern gives rise to.**

After identifying the hierarchy and the typical thoughts that are part of the process of worry/rumination, the therapist explores which experiential avoidance strategies the client puts into practice to avoid the distress generated.

- Explore whether worry/rumination gives rise to implementing additional experiential avoidance strategies. Examples: "According to what you tell me, it seems that Thomas spends many hours a day mulling things over ... I suppose that must consume lots of energy... Can you identify yourself doing something to stop thinking?" "Have you ever felt the desire to stop thinking? What do you usually do to try it?" "It is quite common for people who mull things over to try to do different things to stop thinking; can you identify yourself doing something with that goal?"
- Exploring the usefulness of experiential avoidance strategies. Examples: "Would you find it useful to do this (e.g., watch TV, call a friend to distract themselves, partying, take drugs, play video games, surf the internet, etc.) to stop thinking? Do you manage to do it? How do you feel when you manage to "turn off" your mind? But, how long does the relief last? How long does it take the "daddy" or

one of his “kids” to return? While you are trying to stop thinking, do you do things that go towards the top-right or bottom-left? Is doing this the best thing that Thomas could do at that time to go up-right?”

During this process, the therapist must complete the down-left part of the diagram by writing down the experiential avoidance strategies provoked by worry/rumination (see the next example).

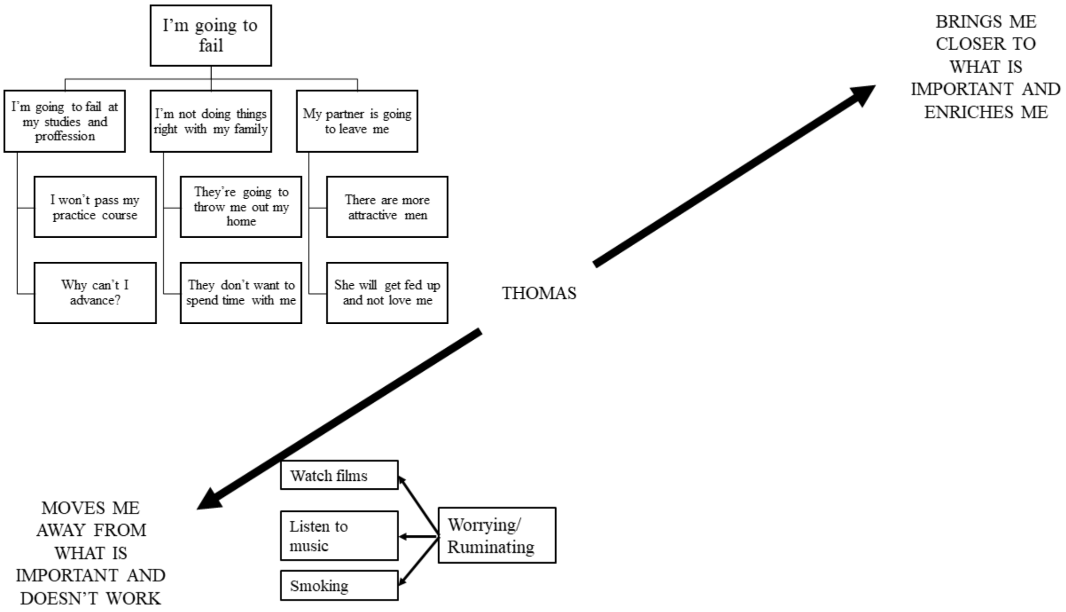

6. **Contacting the consequences of worry/rumination and the other experiential avoidance strategies.**

“Let me try to make an overview of what we have been talking about to see if I've understood your experience correctly. There are times during the day when Thomas gets these thoughts (the thoughts are quickly mentioned, pointing out which is the “daddy” according to the hierarchy built). When this happens, of course, Thomas doesn't like it, he feels bad and begins to mull things over for X time. During that time, Thomas does not do what is important to him and would enrich his life; rather, what Thomas does is controlled by his “Daddy” and his “kids”, as if they controlled his life and Thomas was their slave. At other times, Thomas tries to stop thinking, being entangled, worrying, mulling things over, and for that, he does… (the main experiential avoidance strategies are pointed out). Sometimes, Thomas even does things to try to prevent the “daddy” or the “kids” from appearing, to keep them quiet, etc.”

After the client confirms that as his/her experience, the therapist tries to extend the “inflexible x-ray pattern:”

- Explore how much time the client spends on the inflexible pattern and whether the "big one" is increasingly more present. Examples: "How long is Thomas entangled with all these thoughts and trying to do things to not think about them? Do you feel that during this time the "daddy" is stronger and has more "kids"? Do you feel that the "daddy" is becoming bigger or smaller? According to your experience, if things go on like this, would the "daddy" of your fears ever lose his strength?
- Explore the consequences of the inflexible pattern regarding valued areas. Examples: "How are things going that are important in the life of Thomas? Is Thomas dedicating to them the time they deserve? If we look back, has Thomas improved in the things that are important to him as the "daddy" became more present in his life?

7. **Introduction of a physical metaphor that sums up and amplifies the consequences of the experiential avoidance pattern and proposes an alternative based on discriminating the worry/rumination process, taking perspective and establishing contact with valued directions and actions.**

"I propose that we do a little exercise that may be similar to what happens when the "daddy" or the "kids" come and you mull things over and over and you end up doing things to make them go away, but that are not useful in your life. I'm going to write the "daddy" on this paper. Imagine that on this other sheet of paper you're doing a job or a report... Something that is important to you in your daily life..." (the task is set depending on the characteristics of the client; the important thing is that the client perceives that this activity would be really important and is the best thing he/she could do at that moment to go up-right).
After one minute in which the client is focused on the task, the therapist abruptly places paper with the "daddy" in the client's face.
"But, at a given moment, the "daddy" appears and stands in front of your eyes... And it seems that you don't like looking at him, right?" (in general, clients remain paralyzed looking at the paper and stop doing their task).

"Then, I get the feeling that you try not to see that paper and you try to remove it by pushing it forward with your hand... Try it... Try harder…"
(The client pushes the paper, but the therapist resists with his arm so that the client begins to experience a great struggle to remove the thought, which is unsuccessful).

"Look at what happens. What happened with the report/work you were doing?" "How much effort did you make to remove the daddy? What did you gain by pushing?"

"The daddy and the children eventually go away and Thomas goes back to writing... But I get the feeling that after a while... They come back again!" (The therapist again places

the paper in the client's face). “And then... What do you do? You push again, right?” ... and after a long while, perhaps they'll go away, but after a while... they're back!... And the client pushes again. Do it. Look, how do you feel after pushing so much? Do you usually feel that way at the end of the day: exhausted, tense, with pain in your shoulders, neck, arms? (In general, clients often feel tension in the neck, shoulders, and back as a result of the stress caused by worrying/ruminating, so establishing this similarity is often useful to establish better contact with the metaphor). In addition, when the daddy or the kids appear, how many important things can you do while fighting with them?”

Is this struggle like what happens to you over time? Is this struggle against the piece of paper what your life is like?

Notice something, what happens when they appear and you start to push? How strong are they when you start to push? Is the slip of paper stronger or weaker when you push? Throughout this time, do you have the feeling that the “daddy” has become stronger and has increasingly more “kids”?

Imagine that you had binoculars and you could see yourself within a year while you continue pushing every time you get one of these thoughts. How do you see you life if you do this for one more year? How do you see yourself? (happy? tired? with gray hair?...). Imagine yourself in five more years... what would you be like then? Do you like your image of your life? It is better or worse than now?... Imagine that what we're seeing is a film. What title would you give it?

However, let's look at something... We have seen that when the “daddy” or the “kids” appear, you make them stronger by pushing them... If by pushing them, you only make them stronger, what do you think you could do? (The answer is shaped until the participant sees that one option is to stop pushing, let the “daddy” contact by placing his hand in a position that allows him to continue with the work/report. After that, we get the participant to write and every so often, thoughts come, and he just lets them touch him and continues to write).

“By letting the daddy or the kids touch you without pushing them away, can you do things that go in the direction of enriching your life, that is, towards the top-right?... Imagine that you learn to let the thoughts touch you without having to be pushing them away and you pick up the binoculars again... How would see yourself in 1 year?... And in 5 years?... Who would be in control of Thomas' life? Thomas or Thomas' “daddy” and his “kids”? What would be the title of the film in this case?

8. **Practice in the discrimination of the process of worry/rumination and perspective-taking with a physical exercise.**

“Now, if you agree, I propose an exercise that can help you to see the difference between pushing and not pushing thoughts, so will be easier to do this in your daily life. The idea of the exercise is to see what the process of becoming entangled in your thoughts is like and how you could stop mulling things over. The exercise may be a little strange, you might think we are being a bit silly, but we are going to do it together and I think it may be of great help. Are you willing?”

“What we are going to do is a simulation of how you become entangled and what you could do to untangle yourself. Have you ever had the experience of reading for quite a while, and then you realize that you have not understood a word because you were thinking about other things?”

“We're going to something like this. I invite you start to reading this book (a book opened at the beginning of a chapter is placed before the client) and after a bit, I will show you one of the thoughts. First of all, I’m going to ask you to try to get entangled, saying the thoughts that come to mind. When that happens, you have to get up from the chair, put it in the middle of the room, and walk around it. Walking around it is similar to thinking about the thought that just emerged and when you think about the thought, another related thought emerges... And then, as you are choosing to be entangled, you go around again, and another related thought comes... okay? The idea of the exercise, as I mentioned, is that you can see what the tangle is made of. Are you ready?”

The client starts reading. 15 seconds later, the therapist places the paper on which the “daddy” is written on top of the book and says: “here is the 'daddy', what do you choose to do?”

The client gets up and is given the post-it with the “daddy” on it, and is asked to put it in front of his face and begin to turn it around with his hands while he is walking around the chair. When the client has completed a turn, he is asked: and now, what would come to mind, or after this thought, what other thought emerges? When the client states the next thought, he receives another post-it and is told: “and now, Thomas can choose what he wants to do with his thought. What you choose to do?” The client begins to turn the post-it over and is asked to notice that he is the one who has chosen to continue turning it over: “Look at how Thomas is the person who has chosen to continue mulling over his thoughts.”

Repeat the process with 10 thoughts.

When the previous part of the exercise ends, the client is told: “How did you feel mulling over your thoughts? Are you tired? Is this sort of like what happens in real life? How much progress did you make reading while turning your thoughts around?”

“Thomas, take a look at one thing... When you chose mull over the “daddy,” another thought that you don't like appeared right away, right? And, at that time, you chose to turn that thought over, and what happened? Another came... And you chose to turn another one over and... The next one came... how long could this process last? How would you end it?”

“The funny thing is that Thomas really could choose what to do with every thought. In the previous part of the exercise, you always chose turn the thought around, but now we are going to do the opposite. You are going to read the book and, from time to time, I'll put some papers on it, as before. In this case, what I suggest you to do is to simply look at the paper and gently put it aside, be aware that you have chosen to put it there and keep on reading... After a little while, I'll do the same thing again and I invite you to choose to put it aside gently, to realize that Thomas can choose and continue reading.”

10 thoughts are presented.

“How did you feel during the exercise? Could you continue reading? What was the book about?... Look at the difference between pushing thoughts away and walking around the chair, or choosing not to fight with them, let them be and engage in what can enrich your life…

During the next week, I invite you to practice stopping pushing and mulling over the “daddy” and “kids”. There may be times when, without realizing it, you find yourself entangled with your thoughts. It doesn't matter, remember that, at every moment, you can choose to mull over the thought that you are having or let it be. There will be days when you even get angry with yourself for having become entangled. You could even choose to get entangled about why you've become entangled... I simply suggest that you not mull over them and you start to do something that is important for you.

AUDIO 1

This audio aims to help you develop the ability to realize the difference between being entangled and not getting entangled in thoughts and to learn to let them be, choosing to do what is best for you…

We suggest you do some exercises to allow yourself to notice sensations and thoughts. If you wish, you can close your eyes to concentrate only on my voice... If at some point you get distracted with a thought, don't bother about it, just welcome it and choose to continue to listen to my voice here and now…

Start by choosing to pay attention to your breathing. Simply notice how you inhale air and then, how you exhale it... Do not attempt to alter the rhythm of your breathing, simply notice the feelings caused by your becoming aware of your breath…
Realize that you are choosing here and now to focus on noticing your breathing. Notice how the breath is a part of you but normally, you do not pay much attention to it. Keep on noticing the feelings caused by breathing for a few seconds (15-second pause).

Now I would like to invite you to think for a minute about things that are important to you during the day or during the next 24 hours... If you wish, you can make a list on paper or you can just do it in your head. Choose for a moment to think about what things you can do today that can enrich your life (30-second pause).

Well, now try to imagine doing one of those actions that you have thought can enrich your life. You can choose which one you want, but we recommend you imagine one that you believe that will be harder because your mind will surely come up with some thoughts, memories, or feelings with which you can become entangled…

Allow yourself to imagine the situation in which you would perform that action that would enrich your life... where is it? ... Are there more people around? … What would be happening? … Allow yourself to experience for a moment the thought that may appear at this time that could prevent you from doing what is important to you…

Then, when this thought appears, we propose that you make an effort to become entangled and mull over it. Notice how you choose to be entangled here and now and see what other thought comes to mind... Notice that this thought is yours and that when it comes, you can choose to be entangled... Be aware that you choose become entangled and let the next thought arrive... Notice for a moment what comes... And realize that when it comes, you can choose to remain entangled... Entangle yourself and go on to the next thought, which would mean more mulling thoughts over... Notice for a moment how you surely lost contact with what you were going to do... And get entangled with the next thought that arrives... Notice again how a part of you chooses again to be entangled... Look at what

would happen if you continue choosing to be entangled in thoughts, if you continue pushing them... how long would you remain entangled? Would you be proud at the end of the day about the time spent pushing your own thoughts?

Imagine again the situation in which you would do the action you mentioned... Notice the thought that could come to mind and choose whether you want to fight it or, on the contrary, put it on a balloon and simply observe it, let it float freely in the air... Do not try to change it, whatever the thought, it's okay... Just realize that you are the person who is noticing that thought, and you can choose how much weight you want that thought to have in your life…

Notice yourself choosing to perform the action you had in mind... It is likely that while you are doing it, another thought will come that could entangle you... Allow yourself to notice it and, again, choose whether you want to fight it or whether you prefer to put it on another balloon and let it float freely in the air... Notice yourself choosing to do this, and quickly go back to the activity you were carrying out…

Remember that, throughout the day, lots and lots of thoughts will come to mind, some may be pleasant, others neutral, and others unpleasant... Remember that no matter what they are, you can choose what weight you want them to have in your day... As always, the question is: what do you choose to do with your thought: get entangled in it or let it go while focusing on what's important to you?

We have finished the exercise. If you wish, you can practice alone with any other situation in which you can become entangled during the day. In any case, remember that the important thing is to develop the ability, and that this skill, like riding a bike, only develops by doing the things that are important to you, noticing any thoughts that may appear in your path without starting to fight them. Also remember that it doesn't matter if you sometimes become entangled with your thoughts. You can always start to choose to notice them, choose to stop fighting, and start to do what is important at this time.

SESSION 2

1. Initial defusion exercise

“To start this second session, I invite you to do a short exercise with your eyes closed to remember some of the things we saw in the first session and keep practicing letting thoughts and emotions be without struggling with them, pushing them away, or mulling over them. To do the exercise, it's better for you to close your eyes so that you can focus better.”

I'd like that you focus on your breath... You should notice how air goes in and comes out of your nose... If, at some point, you notice that you are distracted because some thoughts or worries come to mind, simply notice it, realize that they are thoughts YOU are having, welcome them, and choose to focus on the exercise we are doing…
Allow yourself to take a deep breath and notice how swollen your chest and your belly become... Notice the rhythm of your breathing without trying to alter it, your only goal at this time is to observe it…
Now, let's focus on what is the air that enters your nose is like. Is it cold or hot? Stay there, noticing how cold air enters and then, how hot air comes out…
Notice how that air enters like a balloon swelling up in your chest and belly and goes out deflating afterwards... Notice how that feeling comes and goes and you are simply here and now choosing to notice it without doing anything about it... Just noticing that it is a feeling you have at this time…
Answer the following questions to yourself: who is breathing?... Who is feeling that sensation, that balloon that inflates and deflates? ... Who is choosing to observe that feeling here and now? … Who is behind all this that you're feeling?... OK, let’s allow 10 seconds so you can see yourself there, choosing to focus on how you take in and let air out.

Now I invite you choose to focus on the sounds we can hear around us... Notice that you can choose to do anything, that YOU have the ability to choose what you want to do... And observe yourself choosing to listen to the sounds around us. Focus on the sounds coming from the hall... if you notice sounds from somewhere else, it doesn't matter, welcome those sounds and observe yourself choosing to focus on the sounds from the hall…
Tell me, who is listening to those sounds, that noise? …

Now I invite you to contemplate the thoughts that come to mind... I would like you to observe that the mind never ceases to present thoughts, one after another... What I'd like is to invite you here and now to notice those thoughts coming, just observe them, look at them, as if you were looking at a landscape that you like or a picture... You simply observe it without trying to change it, without straining against the thought…

Notice what thought visits you right now. Anything that comes to your mind is OK... Right? Now, imagine that you are holding a balloon in your hands... Imagine that you write on its surface the thought you are having right now... OK? … Just notice that the thought is there, on the balloon, and watch it... Allow yourself to choose not to fight it... Not to argue with it... Do not push it... Realize that you can simply observe that written thought... Tell me, who is watching that thought there?…
See how, if you wanted to, you could choose to argue with that thought, think about why you had it or try to push it away, but you are simply here and now, choosing voluntarily to observe it without doing anything with it... I invite you simply choose to let it go and allow it to float freely in the air.

Notice that you can now choose between staying focused on this thought or letting another one appear in your mind... How about you choose to let go of the thought of the balloon and allow yourself to notice what thought comes to mind now? …
It can be any thought, simply notice what thought comes... OK? What thought appeared?... Notice that you can choose to put that thought on the balloon…
Notice that the thought is now on the balloon and that you are choosing simply to watch it, not to confront it…
Realize who is having this thought, who is choosing what to do with it, and who is capable of observing all that and answering this question…
Now just allow yourself to let go of the balloon and let it float freely in the air…

Now, look at what other thought comes to mind... Just notice it and write a word that summarizes it on the balloon. Notice who has chosen the word that summed it up and notice who is choosing here and now to contemplate that thought without opposing it…

What you choose to do now? Stay focused on that thought or give way to another thought to keep practicing? … OK, notice yourself making that choice and see what other thoughts appear in your mind... Simply write a word that summarizes it on the balloon... Notice who has put that thought there and who is choosing to observe it without doing anything with it, without judging whether it is good or bad.

2. **Review of the experience after the first session**

"Have you had the experience of noticing the "daddy" and some "kids" and not get entangled with them like you just did? Have you felt entangled in your thoughts? Was it as usual or did you notice that you've untangled sooner than normal? Did you do different things that can enrich your life?

The goal of this session is to work on developing the ability to be with the thoughts and not get entangled in pushing them away or mulling over them. To do this, I'm going to propose various exercises to practice. Do you agree?

3. **Free association exercise**

“Now, I invite you to do a simple exercise to let thoughts be and not become entangled with them. The aim of the exercise is for you to contemplate what thoughts, memories, or images come to mind in reaction to some words that I will say to you. That is, all you have to do in this exercise is to notice the first thing that comes to mind spontaneously and realize how you can choose to just let that be, or you can mull over it, trying to understand it or push it away. If more things come to your mind after the first thought, simply realize that they are other thoughts that have visited you, and you can choose whether to let them be or push them away and make them stronger. Are you ready?”

Mango... (7 seconds)
Book... (7 seconds)
Holiday... (7 seconds)
Name of the person (e.g., Thomas)... (7 seconds)
House... (7 seconds)
Study... (7 seconds)
Fear... (7 seconds)
Daisies... (7 seconds)
Justice... (7 seconds)
Worry... (7 seconds)
Dream... (7 seconds)
Tangle... (7 seconds)

“How did it go? How was your experience? Could you notice how thoughts and images came to mind? Have you noticed yourself realizing that thoughts are your own transient creations? Have you noticed yourself choosing to let them be without mulling over them?”

4. **Exercise of becoming aware of fantasizing and worrying.**

“In the next exercise, I suggest, first, that you fantasize about something pleasant (for example: being on the beach or seeing your favorite landscape) for a couple of minutes. You can choose to do the exercise with your eyes open or closed. Every so often, I will knock on the table with my hand as a signal to make you aware that it is you who are consciously choosing to fantasize, to make you realize that you have created this fantasy, and for you to see that only you can choose whether to carry on with it or to let it go.”

ALLOW 2 MINUTES AND KNOCK ON THE TABLE EVERY 20 SECONDS (6 TIMES DURING THE INTERVAL).

“Now I propose you do the same thing with important worries that you had during the past week. Simply try to worry and, every so often, I will make the same sound to remind you that it is you who are choosing to worry and that you could stop if you wished.”

ALLOW 2 MINUTES AND KNOCK ON THE TABLE EVERY 20 SECONDS (6 TIMES DURING THE INTERVAL).

“How did it go? Did you become aware of how you were fantasizing or worrying? Is it different from what happens in your life?”

5. **Eye contact exercise.**

“Now I invite you to do a slightly more complicated exercise, but which can be very useful to keep practicing. I invite you to look me in the eye for a few minutes and just notice at each moment what thought and feeling comes to mind. It is likely that during this time, we will feel some discomfort, embarrassment, etc. The only thing we have to do, you and I, is to notice those thoughts and feelings, letting them be and choosing to continue to stare. Consider that to keep on looking is the most important thing you can do now to get closer to what is most important for you. So, are you willing to do the exercise? … Notice who just made that choice... Let us begin…”

THE EXERCISE IS CARRIED OUT FOR APPROXIMATELY 2 MINUTES. IF THE PARTCIPANTS GETS DISCTRACTED, THE THERAPIST GENTLY ASKS HIM TO NOTICE THE THOUGHT OR FEELING THAT DISTRACTED HIM AND CHOOSE TO FOCUS AGAIN ON THE TASK.

When it is over, review the client's experience: was he able to notice the thoughts coming and continue with the task?

6. **Exercise of counting backwards by 3s**

“Now, we'll do a simple arithmetic exercise, but we are going to put on weights to be able to exercise. I invite you to count backwards from 100 by 3s. That is: 100, 97, 94, 91... In the process, I'll say some of the thoughts that tend to distress you and that you usually remember. Your goal is to simply notice that they came, let them be, and choose to continue counting…”
The aim of this exercise is to use an arithmetic task that is complicated for the participant so that doubts may arise during the task about whether or not he is doing the task well,

the participant self-corrects himself, etc. The therapist must simply indicate to the participant that it doesn't matter whether he is doing well or badly, the only goal is to keep on doing the exercise until reaching zero.

7. Writing with the non-dominant hand

“Now, I invite you to do the following exercise. Like the other day, imagine that you have to write a very important report. This report is a giant step in the direction of what you want in life. However, you have to write it with your non-dominant hand... The aim of the exercise is for you to notice the feeling of difficulty, notice any thought that comes to mind while you are writing that it is not related to the report and let it be... Simply notice that it is not easy, observe the discomfort... “Notice that this discomfort and difficulty is part of doing something that is very important to you, and choose to continue trying to write without mulling over whether it is more or less difficult.”

At the end of the exercise, review the client's experience, emphasizing how, despite the discomfort and the feeling that he was not writing clearly, he chose to continue writing.

8. Exercise of suppressing versus letting it be

“Now, I invite you to do a little experiment about something that often happens to us. Sometimes, we don't want to think about something and, as we saw the other day, we begin to push it to make it go away. Let's see what happens when we try to get thoughts out of our head. Close your eyes and imagine a pink elephant... you see it? Now, imagine that, in order to do the most important things in your life, you should, by any means, not have that thought or image. Imagine that in order to do those things that are so important, you have to leave this room, but you cannot leave the room while you have the image of the pink elephant in your head... Therefore, try to erase it from your mind because if the image is there, even if only in the background, you cannot leave the room.”

Allow 2 minutes to try to do it…

“How did it go? Could you ever go through the door and start to do what is important for you if you first had to completely eliminate the image of the pink elephant?”

“Now let one of the thoughts that we talked the other day come to mind. Ready? Try to do the same... try, by all means, to stop thinking about it, throw it out of your mind before you can go towards the door…”

Allow 1 minute…

"Does this resemble what sometimes happens: that you do not engage in doing the things that are important to you because you are struggling so as not to think certain things?"

"Now, try to carry on with that thought in your head, let it come or go freely, while you walk towards the door and open it to go in the direction that you want in your life."

The participant gets up and goes to the door, noticing the thought…

"Could you do it? What if instead of trying to throw out the "daddy" and "kids", you let them be while you open the doors of your life?"

9. **Exploration of valuable actions to perform during the following weeks and specification of barriers (5 to 10 minutes)**

"If you agree, I propose we now talk about the doors that you can touch and try to open during the next week; that is, those actions, however small, that might bring about a change in your life. Actions that you will carry out and that feel proud of having done at the end of the day, because it is important for you.

What would they be? (Exploring in different areas or facets of the person)

What specific actions would you have to do? Where? When?…

Taking into account how important it would be to perform these actions, what could stop you? What "kids" could stand between Thomas and doing the things that are important for Thomas? What could Thomas do when these thoughts and/or emotions arrive? Could you could apply the exercises we did: that is, let those thoughts or emotions be without struggling with them and focus on doing what is important?"

10. **Closing and introduction of exercises in audio format and commitment to doing them**

"During the next week, I suggest that you practice with a few audio files that I will send to your email. In them, you'll find exercises similar to those we have done today. It is important that you practice every day with the audios and that you change from one to another to make the practice as varied possible. The idea is not for you become an expert in a particular exercise, but instead for you to have the most varied practice possible because research reports that the more varied the practice, greater effect it can have. Remember that the idea is to develop the ability to let the "daddy" and "kids" be, to notice the difference between being entangled and not entangled, and see how, at each step, you can choose to focus on what is more relevant to your life instead of unproductively mulling things over in your head.

AUDIO 2

In this audio, I invite you to do a simple exercise in order to notice what thoughts come and learn to let them be without pushing them away or become entangled with them. The only thing you have to do in this exercise is to contemplate what thoughts, memories, or images come to mind when listening to some words. Just notice that those thoughts are momentary things that just come to your mind and allow yourself to notice that you can choose to simply observe them and stay focused on the exercise. If at some point, you are distracted, it doesn't matter, simply notice how your mind took off in another direction, be nice to it, and go back to concentrating on the exercise.

Let's start, just focus on my voice and notice what comes to mind with the following words:

Papaya... (7-second pause)
Computer... (7-second pause)
Study... (7-second pause)
Sadness... (7-second pause)
Youth... (7-second pause)
Anxiety... (7-second pause)
Failure... (7-second pause)
Heat... (7-second pause)
Worry... (7-second pause)
Pain... (7-second pause)
Cinema... (7-second pause)
Tangle... (7 seconds pause)
Salad... (7-second pause)

We have finished the exercise. For a moment, think about this experience. Notice how the thoughts and images came to your mind. Realize that each one that you had during these minutes is a momentary thought and that, faced with any of them, you can choose whether you strengthen it or let it be.

AUDIO 3

The following exercise aims to make you aware of your thought process and how it sometimes runs on auto-pilot. For this purpose, we propose, firstly, that you fantasize about something nice for a couple of minutes (for example: being on the beach or watching your landscape or favorite movie). Every so often, you will hear a sound like this... (A noise is made like knocking on a door, for example). That sound will be a cue to make you aware that it is you who are consciously choosing to fantasize, to realize that you have created this fantasy, and to see that only you can choose to stay with it or let it go.

ALLOW 2 MINUTES IN SILENCE AND KNOCK ON THE TABLE EVERY 15 SECONDS.

Now, I suggest you do the same thing with some worry that you had during this past day or past week. Just try to worry and every so often you'll hear the same sound so that you will remember that it is you who are who are choosing to worry and that you could stop if you choose to.

ALLOW 2 MINUTES IN SILENCE AND KNOCK ON THE TABLE EVERY 15 SECONDS.

We have finished. We suggest that you try to apply this exercise at different times of the day in which we tend to function on auto-pilot. For example, you can use it while you shower, have lunch, walk, etc. Just allow yourself to notice every so often what you're thinking and feeling at that moment and to choose whether you want to continue on auto-pilot or simply concentrate on what you're doing.

AUDIO 4

In this exercise, I invite you to close your eyes and concentrate on my voice. If at some point, you realize that you are distracted, simply notice what thought distracted you, let it be, and notice yourself choosing to refocus on my voice.

Focus on your breathing for a few moments... Try to notice how air goes in and comes out of your nose... Allow yourself to take a deep breath and notice how your chest and belly swell up... Notice the rhythm of your breathing without trying to alter it; your only goal at this time is to observe it…

Notice how the air enters like a balloon swelling up in your chest and belly... And how it goes out afterwards, deflating... Notice how that feeling comes and goes, and you are simply choosing to notice it without doing anything about it... Just notice that it is a feeling that you have at this time and that it is not necessary for you to try to change it…

Now, allow yourself to feel the sensations of your body. Notice whether the place where you are sitting or lying down is soft or hard. You choose to notice the sensation produced in your body without trying to alter it. Focus your attention on the fact that you can just do it without judging whether the sensation is pleasant or unpleasant…

Also notice the position of your arms and your hands and where they are supported. Choose not to judge whether they are in a proper or improper position. Simply, allow yourself to be aware of where they are and the feelings that are produced without trying to change them.

Now I invite you to contemplate the thoughts that come to mind... Notice what thought visits you right now... Anything that comes to your mind is OK... Imagine you have a balloon in your hands and you write the thought you are having at this moment on its surface… Simply notice that the thought is on the balloon and watch it... Allow yourself to choose not to fight it... Not to argue with it... Not push it... Realize that you're capable of simply observing that written thought... I invite you choose to release it and let it float freely in the air.

Notice that you've let the balloon with your thought float in the air... And look at what other thoughts appear in your mind... Notice that you can choose to put that thought on the balloon…

See how the thought is now on the balloon and you are choosing to look at it and not confront it... Realize this: who is having this thought? Who is choosing what to do with it? And who is capable of observing all that and answer this question?... Now just let yourself drop the balloon and let it float freely in the air…

Now, notice what other thought comes to mind... Just notice it and write a word that summarizes it on the balloon. Notice who has chosen the word that summarized it and notice who is choosing here and now to contemplate that thought without opposing it…

What you choose to do now? Stay focused on that thought or give way to another thought to keep practicing? Notice yourself making that choice and see what other thoughts appear in your mind... Simply write a word that summarizes it on the balloon... Notice who has put that thought there and who is choosing to observe it without doing anything with it, without judging whether it is good or bad.

We continue with the exercise, but instead of focusing on the thought that comes to your mind, give yourself permission to think the thoughts that often lead you to begin mulling things over. Take a few seconds to imagine a recent moment and situation, perhaps today, yesterday, or last week, in which you had one of those thoughts that lead you to get entangled and fight them...

Allow yourself to remember where you were... was someone else there?... What began to happen that you made you begin to get entangled in your thoughts?... Take a look at what seems to have been the thought with which you started to get entangled... Notice that you can choose to put that written thought on the balloon and watch it. Do you choose to do that or do you prefer be entangled, fighting or arguing with the thought? Notice yourself choosing to leave it on the balloon and contemplate the thought without doing anything else... Imagine yourself choosing to do this... where would it lead you, what important things could you manage to do?... and if you did this one more year? ... And five years?

Now, I invite you to imagine another recent situation or moment in which you've been entangled, mulling things over... Where are you?... How did you get here? … Who else is there?... What starts to happen?... Take a look at what seems to have been thought that started to entangle you... At this point, I invite you to put that thought on the balloon to notice if this allows you to see it without reacting to it by fighting it... Notice yourself choosing to put it on the balloon. Contemplate the thought written on the balloon without doing anything else... which alternative you choose: fight the thought or weaken it, let it be and focus on what is important to do at that moment?…

Finally, I'd like you to allow yourself for a moment to remember all the thoughts you've had since we started this exercise... Realize the heap of thoughts you have had and that each one of them is yours.

Allow yourself for a moment to think of all the thoughts and emotions that you have had throughout your life…

And allow yourself to think of every thought you will have in the future, from this weekend to within 10 or 20 years…

Realize that all of them are yours, that they are a momentary part of you. Notice how, throughout your life, some thoughts have given way to others, and some emotions to others…

Notice how all the thoughts and emotions are transient, momentary, and that you can choose the weight you want to give them. You can choose to give them weight by fighting them, arguing, trying to eliminate them or escape from them, or you can simply release them and let them be floating in the air, sometimes going and other times coming, while you do the things that are important for you.

Notice, finally, that letting go of thoughts and emotions, setting them free as you did with the balloons and focusing on what is important is a skill that can be learned, like you learned to ride a bike. If, at some point, you fall and you start fighting thoughts and emotions, it doesn't matter, you have fallen many times, the only thing that matters is your determination to learn to let them be and do what you can enrich your life.

SESSION 3

1. **Review of functioning since the last session.**

Review of the session utility: How did it go since the last session? Do you think the session was useful to you? In what things did you notice that? What things have you remembered about it?

Using the audios: Have you used audios? How and how many times? Did they work for you? What for? (If he did not use them, explore why he did not uncritically and whether he thinks doing so could better develop the ability). Explore at what point he could do it and plan.

Valuable actions: How did you fare with the actions you wrote down in the last session? What do you feel proud of having done? What do you not feel proud of?

Triggers and reaction: with what bosses or little bosses have you entangled these days and that have prevented you from fulfilling your commitments? (Include "new bosses" within the others if they are hierarchically related to them; that is, if a "boss" appeared during this time but it turns out that it is contained in one of the already explored bosses, point out the hierarchy and how those bosses have "little bossy" thoughts).
Did you see yourself pushing when the bosses arrived? Do you have the feeling that you gave the boss more strength when you pushed? Was it as if you gave him the reins of your life for a while? How did you push? What were you doing? Are there things you used to do to push that you've stopped doing? Have you seen yourself doing new things?

2. **"Life Film" exercise.**

Today I propose to do an exercise. The objective of this exercise is that we see much more clearly the direction towards the right; that is, we shall see what things would make PEPE proud and what things he can do in that direction. Do you agree?

I invite you to get comfortable and close your eyes. For a minute, imagine that the most important thing right now is to be feeling how your legs and buttocks are resting on the chair... Notice whether the chair is soft or hard... Realize that you can choose to notice the feeling caused by sitting on the chair and that it is you who are observing that... Center your attention on the fact that PEPE has the ability to simply do it without judging whether the feeling is pleasant or unpleasant... Notice also the position of your arms and your hands and where they are supported... Let PEPE choose not to judge whether they are in an adequate or inadequate posture... Simply, allow yourself to be aware of where they are and the sensations they provoke without trying to modify them... Realize how you can also choose to notice the room temperature and whether some parts of your body are

colder or hotter than others... Finally, notice how PEPE is the one who can choose to focus on his breathing, notice its rhythm and without trying to modify it, let it be and just realize that he is the only person who can observe this…

In this exercise, I invite you to make a small autobiographical film. Imagine that you are sitting in an armchair in a movie theater and that they are going to project the movie of your life, your dreams and your disappointments, your desires and concerns... Let a picture of PEPE appear on the screen when he was younger... Allow yourself to have an image about a time when PEPE did not fight his bosses; that is, fighting his own thoughts and emotions... Allow yourself to look at the dreams that PEPE had at that time... (Pause 30 seconds). Allow yourself also look at what things PEPE felt proud to be doing... (Pause 30 seconds).

Now give permission for the screen to project images of what your life is like today. Simply project on the screen images and scenes of the things you usually do in those areas that are important to you. If it's okay with you, let's start with your family, and allow yourself to notice what things you currently do that the PEPE of years ago was proud of. Simply project some images related to that pride on the screen and allow yourself see how you can watch them from your seat. Allow the images to be projected and to have their own life without trying to modify them. Just be the person who allows himself to see those images... (Pause 30 seconds).

Tell me, what do you see on the screen that makes you proud of what you do nowadays in relation to your family?... No matter whether they are big or small things, just allow yourself to share with me what has appeared on the screen…

Now allow images to appear about what makes you feel proud of your performance in relation to your work, training, or occupations... Do not try to modify the images that come, just be the observer of those images and notice how those images are one more part of you (PAUSE 30 seconds).

Tell me, what do you see on the screen that makes you proud of what you do nowadays in relation to your work, training, or occupations?... No matter whether they are big or small things, just allow yourself to share with me what has appeared on the screen…

Now allow images to appear about the actions that make you feel proud in relation to your friends and/or hobbies... If you see yourself forcing the image, simply notice that you can take the observer stance of that mental movie and let the screen freely project whatever appears in your mind (PAUSE 30 seconds).

Tell me, what do you see on the screen that makes you proud about what you do nowadays in relation to your friends and/or hobbies?...

Now let images come about the actions that make you feel proud in relation to your self-care and health, as well as any area that is important for you and that we have not mentioned before... Allow yourself observe the images that appear (PAUSE 30 seconds).

Tell me, what do you see on the screen that will make you proud about what you do nowadays in relation to your health and other issues that are important to you?

Now I invite you to look at the scenes that appear on the screen of your mind in relation to the things you do nowadays about which you do not feel proud, about which the old PEPE would not feel proud either. This can be a bit difficult, but notice yourself letting the images flow and allow yourself be their observer (PAUSE 30 seconds).

Tell me, what do you see on the screen that you're not proud of?

Give yourself permission now to imagine or dream how you would like your life to be in 1 year... allow projecting on the screen what PEPE would be doing, what he would really dream of if he allowed himself... If you notice yourself modifying those scenes because you have the urge to lower your aspirations so as not to feel bad if you do not fulfill them, I just invite you to notice that PEPE is having that impulse and just let it be without entangling with it (PAUSE 30 seconds).

Also, during this exercise, I ask you to give me permission so that, when you see that your mind tries to sabotage you with buts, worries, or self-evaluations, we shall put these on a balloon and simply choose to let them be on it.

How would you like your life to be in 1 year? Take a minute to see the projected images on the screen and, when you're ready, tell me what you see.

Now allow images to come of how you wish your life were in five years. Take a few minutes to see the screen and when you're ready, tell me what you see.

Finally, allow yourself look at how you wish your life were in 10, 15, or 20 years. Allow yourself look at the screen without modifying the images and tell me what you see when you're ready.

Now I invite you to retake everything we've seen so far and let yourself feel how full your life would be, how you would feel after 10, 15, or 20 years doing those things that you would feel proud of in the different areas that are important to you. Realize who is the person who is imagining this and that without that person, those images would not exist, they would make no sense or have any reason for existing.

Now I invite you to come up with a few pictures of what your life would be like if you chose to allow the bosses (fears, memories, worries, etc.) to be the ones who command your life, if you get entangled when they come and you stop doing what is really important to you. How would your life be in 1 year if you continually allow the bosses to command?

Which bosses would have been commanding, those bosses who are the ones who support the rest of little bosses who can appear every day?

For a moment, I invite you to look them in the face and let yourself notice and feel them. Notice yourself choosing to put them on a balloon... and notice how by watching them without doing anything with them, PEPE has the ability to choose what kind of life he wants to have: the one he dreams about or the one the bosses command him to have. Moreover, notice how, as those thoughts on the balloon are yours, you can choose the strength you want them to have.

What would happen, what would you do if you chose to let that boss command? What other bosses would come? You can put each of them on a balloon... can you notice how, even if all those balloons come to you, you could still choose what you want to do: whether you are the boss and continue doing the things that you would feel proud of and that enrich your life or you let them command and watch how your life deteriorates?... Allow yourself for a moment feel how those bosses sometimes try to deceive you and give you excuses for not doing things that are important to you and try to keep you calm, but it is a tense and unproductive calmness... Notice also how, although it may be unpleasant to have those thoughts and let them be on the balloon, you can choose whether to let them control your life and entangle you with their excuses or whether you prefer to choose to do what is important to you and come each day a little closer to the satisfaction you felt a while ago when you allowed yourself to see what your life would have been like.

We have finished…

3. **Summary of the experience with the previous exercise, the garden metaphor and the commitment to valuable actions.**

Now I invite you to look to the right side of the outline, that is, to what is really important to you in life. The therapist summarizes the experience of the previous exercise and the things that seem important to the patient. Then, he introduces the garden metaphor.

GARDEN METAPHOR AND ASSESSMENT OF THE STATE OF VALUES: "Imagine that you live in a world where you have been a gardener, and what matters most to you in life is your garden. In it are the plants you love with all your heart, and spending all

day caring for them and watering them would make you be tremendously satisfied with your life”.

“According to what we saw in the previous exercise, what plants would you have? Can you think for a moment of the four plants that are most important to you?... What are they?”

“If you could rate how big, lush or pretty those plants are in your life, what score would you give them from 0 to 10? Could you water these plants and at the same time continue fighting with the “boss” and the “little bosses”? What would these plants be like from 0 to 10 after one more year of fighting the boss and the "little bosses"? And after 5 years?”

IDENTIFICATION OF VALUABLE DAILY ACTIONS AND BARRIERS: "Now, allow me to see several actions that you could commit yourself to do in your daily life to care for your plants, and that you are not doing because you are entangled in your mind." The patient and therapist collaboratively explore possible valuable actions that are concrete and feasible.

“If you did this day by day, how would your plants be in 1 year? And in 5 years?”

4. Closing of the intervention

We've finished the 3 scheduled sessions. Now we propose that you continue practicing. For this purpose, we will send you another audio. The idea is for you to practice for at least a month about 15-30 minutes a day with all the audios that we have sent you: a few days with each one. The ideal is that your practice should be varied, so that, even if you have a favorite audio, you change from day to day.

<u>AUDIO 5</u>

In this exercise, we invite you to focus on the things that are important to you, the things that you would like to do in your lifetime and that would make you feel proud of yourself, enthusiastic and excited because they allow you to approach the person you want to be and the life you want to live.

For this purpose, imagine that your life is like a path that you walk down day by day. On some days, you advance a lot; on others, you advance a little; and on others, you will surely remain still. Imagine that, at the end of the path are the important things for you, and each of them is a plant. Think for a moment which plants they would be and how well cared for they are... (allow 15 seconds pause).

Some of these plants may now be wilted, others will have some fallen leaves and no flowers, others will be very green, with fruits, and others will have a lot of weeds around... Allow yourself to see what the plants' status depends on. Who is the only person who can take care of your plants? Can all the plants be very green and beautiful without anyone taking care of them?

As those plants are at the end of the pathway, in order to take care of them every day, you have to walk towards the garden and take different actions to care for them and nourish them. There are days when we didn't even get to the end of the path... Other days, we fertilize them, we dig the dirt, we take the plants out into the sun, and each one of these actions, despite being so small and simple, in the long term, helps the plant to be as you want it to be. We invite you to choose one of those plants, whichever one you want, and for a moment think about what kind of actions you could do today that would nourish that plant. Remember that sometimes small and simple actions are enough, but they should be done on a daily basis. (Allow 15 seconds)

Now imagine that every day you do small actions to care for that plant. How would your plant be in a month? How would your life be in a month? Would you feel that you are closer to the person you want to be?

However, pathways sometimes have stones, slopes, it rains... Once in a while, we can stumble on a fallen tree or fall into a large mud puddle. And it is at those moments that moving towards the things that are important to us, becoming that person we want to be is not so easy... Can you imagine what obstacles can arise in your path? What difficulties may you find and with what thoughts might you entangle?

The question is... When you are walking and you trip on some obstacle, you can turn around and go back to your path and advance in spite of the obstacle or you can deviate from your path, stand still, etc. Who chooses to do one thing or the other? Surely, the

easiest option is to deviate or stand still because advancing sometimes implies that you would have to go through some discomfort. But would it be worthwhile to go through this discomfort if you could care for and nurture your plants? Would it be worthwhile to have advanced, despite the difficulties, in 1 month, 1 year or 5 years?

We invite you to pay attention during the day to what things you could do to water your plants and choose each moment whether you want to advance or stay anchored on your path.
