# Supplementary material for: A Multiple-Baseline Evaluation of Acceptance and Commitment Therapy Focused on Repetitive Negative Thinking for Comorbid Generalized Anxiety Disorder and Depression
Source: Front Psychol. 2020 Mar 13;11:356. doi: 10.3389/fpsyg.2020.00356 (PMC7082425; doi:10.3389/fpsyg.2020.00356)
Supplement: PRESENTATION S2 — Spanish version of the RNT-focused ACT protocol employed in the study. [file Presentation_2.pdf]

PROTOCOLO DE TRES SESIONES DE ACT CENTRADO EN DESMANTELAR PATRONES DE PENSAMIENTO NEGATIVO REPETITIVO PARA DEPRESIÓN Y TRASTORNO DE ANSIEDAD GENERALIZADA COMÓRBIDOS

SESIÓN 1

1. Establecimiento del contexto para realizar una intervención breve.

"El trabajo que te propongo realizar está basado en un modelo de intervención breve que tiene como objetivo intentar conseguir cambios importantes lo antes posible. A veces, como es posible que conozcas si has pasado anteriormente por un proceso terapéutico, las intervenciones psicológicas avanzan de manera lenta y recaban una gran cantidad de información durante la evaluación. En nuestro caso, al proponerte realizar una intervención breve, vamos a intentar ir directamente al grano, al corazón del problema que estás experimentando. Esto tiene la ventaja de que, si el trabajo que hacemos es útil, seguramente lo notaremos de manera rápida. Sin embargo, al ir directamente al grano, es posible que haya momentos en los que te parezca que vamos rápido y que la sesión pueda resultar intensa. Si en algún momento crees que debemos ir un poco más despacio o sientes que hay algo importante que ha quedado en el tintero, siéntete libre de hacérmelo saber. Aunque te propongo realizar una intervención breve, el ritmo del trabajo siempre lo vas a marcar tú y todo lo que hagamos irá en la dirección de hacer el cambio que deseas en tu vida. En cualquier caso, me gustaría comentarte que, aunque estés pasando por algunos momentos difíciles que te han traído hasta aquí, considero que eres una persona completa, responsable y valiosa, que está capacitada para que hagamos un trabajo que va directamente al grano para tratar de que sea útil lo antes posible. ¿Te parece bien el plan de trabajo?"

2. Presentación de la lógica de la intervención.

"En primer lugar, permíteme que te comente cuál es la lógica de la intervención y del trabajo que vamos a realizar. Esta intervención está diseñada de acuerdo con investigaciones recientes que otorgan a la preocupación y la rumia (darle excesivas vueltas a la cabeza, enredarse, etc.) un papel fundamental en el desarrollo y mantenimiento de problemas emocionales en la vida de las personas, haciendo que éstas se sientan más ansiosas y tristes y no consigan cumplir con el objetivo que tienen en su vida".

"Teniendo esto en cuenta, el trabajo que te propongo que hagamos durante estas sesiones es desarrollar la habilidad de detectar cuándo nos estamos enredando en nuestra mente de manera infructuosa y ser capaces de dejar esto estar y centrarnos en lo que es realmente importante en nuestras vidas".

"Antes de nada, permíteme explicarte durante un momento por qué a veces nos quedamos enredados, dando vueltas en nuestra mente, etc. Resulta que todas las personas hemos tenido montones de pensamientos y sentimientos a lo largo de nuestras vidas, los vamos a tener durante el día de hoy y seguiremos teniendo miles y miles en el tiempo que nos

queda de vida. Por ejemplo, seguro que puedes recordar durante un minuto qué pensamientos te venían a la cabeza cuando tenías 8 años… Cuando tenías 15 años… Los que tenías hace un año… Los que tuviste ayer… Los que estás teniendo ahora mismo… A lo largo de la vida seguramente hemos ido viendo que los pensamientos van y vienen mientras que lo único constante somos nosotros, es decir, la persona, PEPE (nombre del paciente). Sin embargo, en ocasiones, nos viene un pensamiento o un miedo relacionado con algo que no queremos que ocurra o un pensamiento relacionado con la necesidad de explicar algo importante y, aunque esos pensamientos son nuestros, solamente algo momentáneo que podríamos dejar pasar como el resto de pensamientos, al ser muy molestos nos olvidamos de que no son más que pensamientos y de que nosotros podemos elegir qué importancia queremos darles… Si los dejamos estar y pasar, porque todos los pensamientos son finalmente momentáneos, o nos enfrascamos con ellos y les damos el mando de nuestra vida por ese rato que a veces se convierte en horas, luego en días, meses e incluso años. Y entonces nos enfrascamos en pensar en ellos, llevamos toda nuestra atención hacia ellos y pensamos y pensamos, nos preocupamos y les damos vueltas, una y otra vez… Muchas veces, ese dar vueltas es muy repetitivo, pero seguimos haciéndolo una y otra vez. Y una y otra vez… En muchas ocasiones, incluso, ni siquiera sentimos que realmente somos nosotros los que estamos eligiendo enfrascarnos. Simplemente, lo hacemos de manera automática, sin darnos cuenta de que en cualquier momento podríamos elegir dejar estar el miedo y la preocupación. Nos olvidamos por completo de que estamos delante de pensamientos que son nuestros y terminamos creando realidades alternativas en nuestra mente…"

"Como te comentaba anteriormente, lo que vamos a intentar hacer es que PEPE aprenda a darse cuenta de cómo ante determinados pensamientos suyos, elige, muchas veces sin saberlo, enredarse y enredarse con ellos… Como si ante el flujo de pensamientos les pusiera un dique o un embalse ante algunos de modo que se quedan atascados y PEPE comienza a mirarlos y a analizarlos una y otra vez hasta que éstos empiezan a dar vueltas como formando un remolino… Y en muchas ocasiones se nos olvida que somos realmente nosotros los que ponemos el dique y formamos el remolino de pensamientos… Sin darnos cuenta de que nosotros podemos elegir abrir el dique, dejarlos pasar y centrar nuestra atención en lo que es fundamental para nosotros…"

"Dado lo que hemos comentado previamente, durante esta sesión quizás notes algo un poco extraño y es que en ocasiones nos detendremos para que notes cómo PEPE está teniendo determinados pensamientos y que notes a cada paso qué estás eligiendo hacer: si cerrar el dique o abrirlo y dejar esos pensamientos pasar para poder centrarnos en lo importante de la vida de PEPE. Por ejemplo, permítete por un momento observar los pensamientos o sensaciones que vienen a tu cabeza en este momento sin hacer nada con ellos. Para ayudarnos vamos a utilizar estos trozos de papel. Sea el que sea está bien… Simplemente, escríbelo en el papel y contémplalo como si fuera un cuadro o un paisaje: sin hacer nada con él, simplemente observándolo. Nota cómo es PEPE el que está

eligiendo contemplarlo y dejarlo estar. Y ahora abramos el dique y dejemos que pase… ¿Quién acaba de elegir dejarlo pasar?”

“Deja que te venga otro pensamiento, el que sea… Lo único que toca hacer en este momento es elegir escribirlo y contemplarlo en el papel… Date de nuevo cuenta de que es un pensamiento tuyo y de que tú puedes elegir si abres el dique o te enredas en darle vueltas a este pensamiento…” Se repite lo mismo con otros 3 pensamientos más.

3. **Exposición del esquema de trabajo y establecimiento de la preocupación/rumia como el foco de la evaluación e intervención.**

“A lo largo de la intervención te propongo trabajar con este esquema. Aquí vamos a poner tu nombre, el nombre de la persona que tiene todos los pensamientos, recuerdos y sensaciones… Por lo general, todas las personas podemos realizar acciones durante el día que van en dos direcciones. Por un lado, podemos hacer cosas que van en la dirección que nos acerca a lo que es importante para nosotros y enriquece nuestra vida, ese tipo de cosas que nos hacen sentir orgullosos, que hacen que cuando nos vamos a dormir pensemos que el día ha merecido la pena y nos durmamos con una sonrisa de satisfacción en la boca, ese tipo de acciones de las que nos sentiremos orgullosos de recordar que las hicimos dentro de mucho tiempo, incluso cuando seamos ancianos. Permítete por un momento notar los pensamientos que vienen a tu mente sin hacer nada con ellos… Solamente nota cuáles son… Escríbelos en el papel o imagina que los escribes… Y nota cómo puedes elegir seguir prestándome atención”.

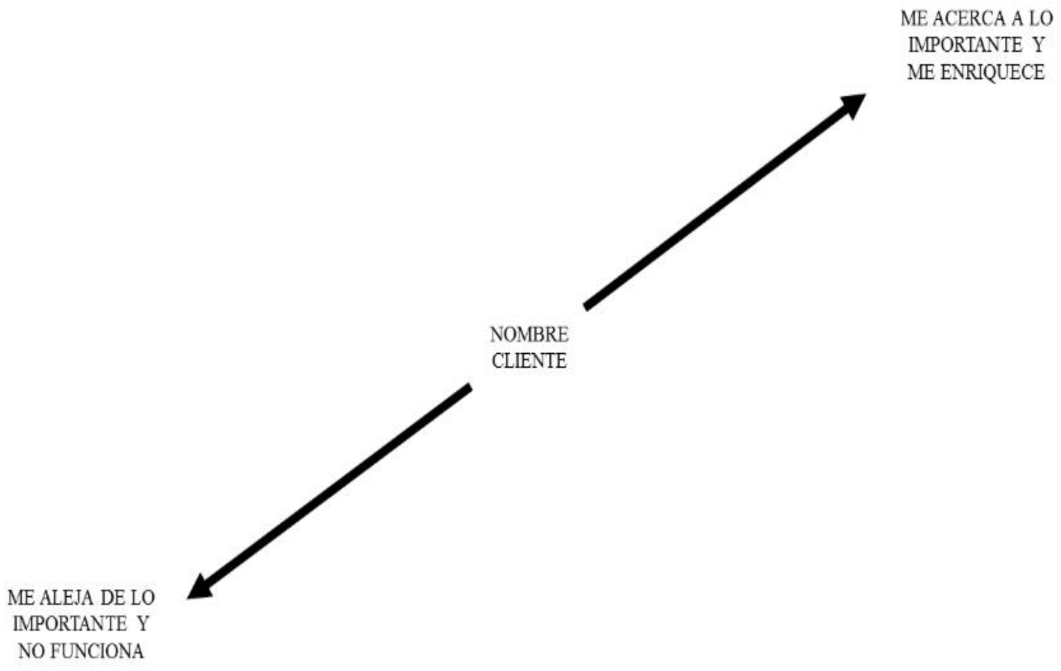

"Por otro lado, podemos hacer cosas que nos alejan de lo que es importante para nosotros o nos mantienen atascados sin avanzar hacia lo que es importante y nos enriquece. Son ese tipo de cosas de las que no nos sentiríamos especialmente orgullosos al final del día o con el paso de los años. De nuevo, simplemente permítete notar qué pensamiento pasa ahora por tu mente sin hacer nada con él, simplemente nótate siendo la persona que lo observa. Prácticamente, todas las personas realizan acciones en ambas direcciones a lo largo del día, unas personas hacen más hacia arriba y derecha y otras más hacia la abajo e izquierda. Habitualmente, las personas que piden intervención psicológica están realizando más acciones hacia abajo-izquierda que hacia la arriba-derecha, y por esa razón se sienten poco satisfechos con sus vidas. El trabajo que te propongo realizar aquí es ver qué cosas haces en tu día a día que te llevan en una u otra dirección, de modo que cada vez hagas más cosas que te acercan a lo que es importante para ti y enriquece tu vida, y cada vez hagas menos cosas que te alejan de lo importante y te mantienen enredado. ¿Tiene esto sentido para ti? Si consiguiéramos esto, ¿podríamos decir que el trabajo habría sido exitoso?"

"En esta sesión te propongo revisar cuánto tiempo pasas haciendo cosas que te alejan de lo importante o, al menos, que no te permiten ir en esa dirección y te mantienen enredado. Muchas veces, las cosas que empiezan a alejarnos de lo que es importante o nos mantienen atascados tienen que ver con pasar mucho tiempo enredados en nuestros propios pensamientos, preocupaciones, recuerdos, etc. Es decir, dándole vueltas a la cabeza, discutiendo con nosotros mismos, pensando en por qué algo funciona así, y así una y otra vez, etc. Darle vueltas a la cabeza no es necesariamente algo malo. Por ejemplo, hay veces en que nos preocupamos acerca del futuro y así anticipamos posibles problemas y le damos solución. Otras veces, pensamos acerca de algo que ha pasado y descubrimos por qué ocurrió y eso nos sirve para el presente y el futuro. Sin embargo, ocurre que en muchas ocasiones pensar demasiado o darle vueltas a la cabeza no es útil y, a pesar de eso, lo hacemos sin parar, perdiendo el contacto con las cosas importantes que están ocurriendo o que podrían hacerse en ese momento. En otras palabras, vamos en la dirección de la izquierda, alejándonos de lo que es importante para nosotros".

4. **Identificación de la jerarquía de disparadores de preocupación/rumia y la cadena de pensamientos principales.**

El terapeuta explora ahora cuál es el evento privado en la cúspide de la jerarquía de disparadores de preocupación/rumia. Para ello, es importante que el terapeuta pregunte de manera especial, a través de metáforas, para facilitar que el cliente responda con la cúspide de la jerarquía (el "papá" de los miedos o cosas que hay que dar explicación). A continuación, se expone una manera de preguntar:

“En este momento te invito a que pensemos durante unos minutos en cuándo te quedas enredado en tu mente. En muchas ocasiones, el desencadenante o disparador de dar vueltas a la cabeza es el temor a que algo ocurra o bien la necesidad de encontrar una razón por la que ocurren ciertas cosas. Imagina que tienes una pistola mágica con la que puedas disparar a los pensamientos que te llevan a enredarte, pero sólo tienes una bala. ¿A qué pensamiento le dispararías? ¿A cuál podrías dispararle con el que matarías a más de “un pájaro” de un tiro? ¿A qué pensamiento podrías disparar que es el papá de tus miedos, el papá que alimenta y da sustento al resto de pensamientos? ¿Qué pensamiento sería al que tras dispararle los demás pensamientos también caerían como un castillo de naipes?”

“Permítete notar por un momento quién acaba de contestar y de quién es ese pensamiento que en muchas ocasiones hace de jefe… Simplemente obsérvalo y mira cómo puedes elegir mirarlo escrito en el esquema sin hacer nada con él… Durante este tiempo, me gustaría hablar con PEPE, no con ese pensamiento que a veces toma mucha fuerza en la vida de PEPE… ¿Qué eliges hacer? ¿Dejamos que hable el pensamiento, el jefe de Pepe o eliges mejor hablar tú? Seguramente, el jefe está ahí por algo y aparece por alguna razón, pero permite que sea PEPE el que habla… Porque nadie sabe más que PEPE sobre la vida de PEPE… Ni siquiera su jefe…”

“Ahora te invito a que hablemos durante un rato de qué está hecho ese jefe que a menudo le viene a PEPE…”

En este punto, el cliente responde señalando un pensamiento que puede ser la cúspide de la jerarquía de disparadores o no. A continuación, se presentan pistas para intuir si el cliente identificó la cúspide o no y preguntas para seguir desarrollando el análisis funcional. El objetivo del terapeuta en este punto es identificar el disparado de la cúspide (“papá”) y los disparadores que contiene (“hijos”), identificando con el cliente cómo es habitualmente el proceso de preocupación/rumia.

- **<u>Pistas que indican que el evento privado señalado por el cliente no es la cúspide de la jerarquía</u>**:
  - ❑ Es un miedo concreto con “fecha de caducidad” (p. ej., miedo a suspender el examen de la semana que viene, miedo a hacer el ridículo en un evento público, una ruptura sentimental, fracasar en algo particular, etc.).
  - ❑ El evento privado no parece resultar excesivamente aversivo.
  - ❑ El evento privado ha aparecido de manera relativamente reciente en la vida del cliente, pero sus problemas emocionales son más antiguos.

- **<u>Preguntas útiles cuando sospechamos que el evento privado no es la cúspide de la jerarquía</u>**:

- Ejemplo 1: El consultante dice que le da miedo no aprobar una materia.

¿Es algo que te ocurre a menudo? Si el cliente señala que sí, es probable que forme parte de algo más general.
¿Suele pasarte en otras áreas distintas a tus estudios? ¿Hay más cosas en tu vida que te da miedo "suspender"? Si el cliente señala otras áreas, es probable que el "grande" sea el miedo a fracasar o "soy un fracaso".
¿Qué te viene a la cabeza cuando piensas que vas a suspender la materia? Si lo que señala el cliente son pensamientos relacionados con la materia (p. ej., "Voy a tener que volver a estudiarla") indicaría que es posible que no haya nada más "grande"; sin embargo, si responde con algo más genérico (p. ej., "No voy a ser un buen profesional", "Dudo sobre si soy capaz") estaría indicando que probablemente haya algo más abarcador como el miedo a no ser un buen profesional o "no soy inteligente".
¿Por qué sería tan malo para ti suspender la materia?
¿Crees que hay un miedo o pensamiento más grande que alimenta a este miedo? ¿Tiene ese miedo un papá que lo engendró?

- Ejemplo 2: El consultante dice que le da miedo que su pareja le deje.

¿Te ha ocurrido con otras parejas? Si es así, puede que se trate del miedo a estar sin pareja.
¿Te pasa también con otras personas aunque no sean tu pareja? Si es así, puede que se trate de miedo a estar solo o no tener protección.
¿Por qué sería tan malo que te dejara tu pareja?
Si tu pareja te dejara, ¿demostraría esto que hay algo en lo más profundo de ti que está mal?
¿Cuál sería el peor pensamiento que podrías tener sobre ti mismo si tu pareja te dejara?

- **Pistas que indican que el evento privado señalado por el cliente es la cúspide de la jerarquía**:

- Es abstracto y de él derivan pensamientos más concretos.
- Está presente en varias áreas de la vida del cliente.
- Es "viejo".
- Resulta extremadamente molesto para el cliente.
- Es el pensamiento que el cliente derivaría en el caso en que su vida no fuera como él quiere.

- **Preguntas útiles cuando sospechamos que el evento privado es la cúspide de la jerarquía**:

- ¿En cuántas áreas de tu vida está presente y te causa malestar este pensamiento?
- ¿Sería éste el pensamiento que más te dolería experimentar como cierto?
- ¿Cuántos años tiene este pensamiento? ¿Tiene "hijos"? ¿Se ha reproducido en miedos más concretos?

- ❑ Si pudiéramos "extirparlo", ¿tendrían el resto de pensamientos molestos sentido o desaparecerían?

A lo largo de la evaluación, el terapeuta trata de dibujar un esquema similar al que se presenta en la siguiente imagen. Es importante identificar la cúspide de la jerarquía y algunos de los pensamientos más representativos por los que transcurre el proceso de preocupación/rumia. Habitualmente, los clientes no son especialmente hábiles discriminando cómo es el proceso de preocupación/rumia, por lo que trata de especificar cómo transcurre suele ser de gran utilidad.

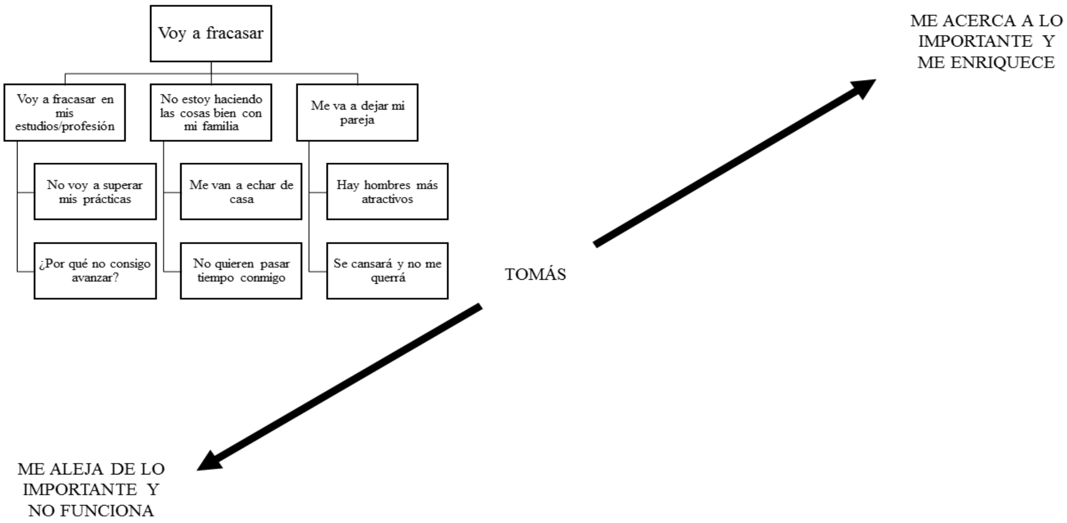

Durante el proceso anterior, cuando PEPE especifica otros "jefes" o "jefecillos", le pedimos que por un momento note cómo eso que acaba de decir es otro pensamiento y que puede elegir en ese momento contemplarlo, dejarlo estar y seguir centrado en la conversación o bien puede elegir darle vueltas a ese "jefecillo" y que se nos vaya la sesión en lo mismo que pasa a diario en la vida de PEPE.

5. **Análisis de otras estrategias de evitación experiencial a las que da lugar el patrón de preocupación/rumia.**

Una vez identificada la jerarquía y los pensamientos típicos que forman parte del proceso de preocupación/rumia, el terapeuta pasa a explorar qué estrategias de evitación experiencial pone en práctica el cliente para evitar el malestar generado.

- Explorar si la preocupación/rumia da lugar a poner en práctica estrategias de evitación experiencial adicionales. Ejemplos: "Por lo que me comentas, parece que Tomás pasa muchas horas al día dándole vueltas a la cabeza… Supongo que eso debe consumir mucha energía… ¿Te ves haciendo algo para dejar de pensar?" "¿Has experimentado alguna vez las ganas de dejar de pensar? ¿Qué sueles hacer

para intentarlo?" "Es bastante común que la gente que le da muchas vueltas a la cabeza intente hacer distintas cosas para dejar de pensar, ¿te ves haciendo algo con ese objetivo?"

- Explorar la utilidad de las estrategias de evitación experiencial. Ejemplos: "¿Te resulta útil hacer esto (p. ej., ver TV, llamar a un amigo para distraerse, ir de fiesta, consumir drogas, jugar videojuegos, navegar en internet, etc.) para dejar de pensar? ¿Lo consigues? ¿Cómo te sientes cuando consigues "apagar" tu mente? Pero, ¿cuánto tiempo dura el alivio? ¿Cuánto tarda en volver el "papá" o uno de sus "hijos"? Mientras estás intentando dejar de pensar, ¿haces cosas que van hacia la arriba-derecha o hacia abajo-izquierda? ¿Hacer esto es lo mejor que podría hacer Tomás en ese momento para ir hacia arriba-derecha?"

Durante este proceso, el terapeuta debe ir completando la parte de abajo a la izquierda del esquema colocando las estrategias de evitación experienciales a las que da lugar la preocupación/rumia (véase el siguiente ejemplo).

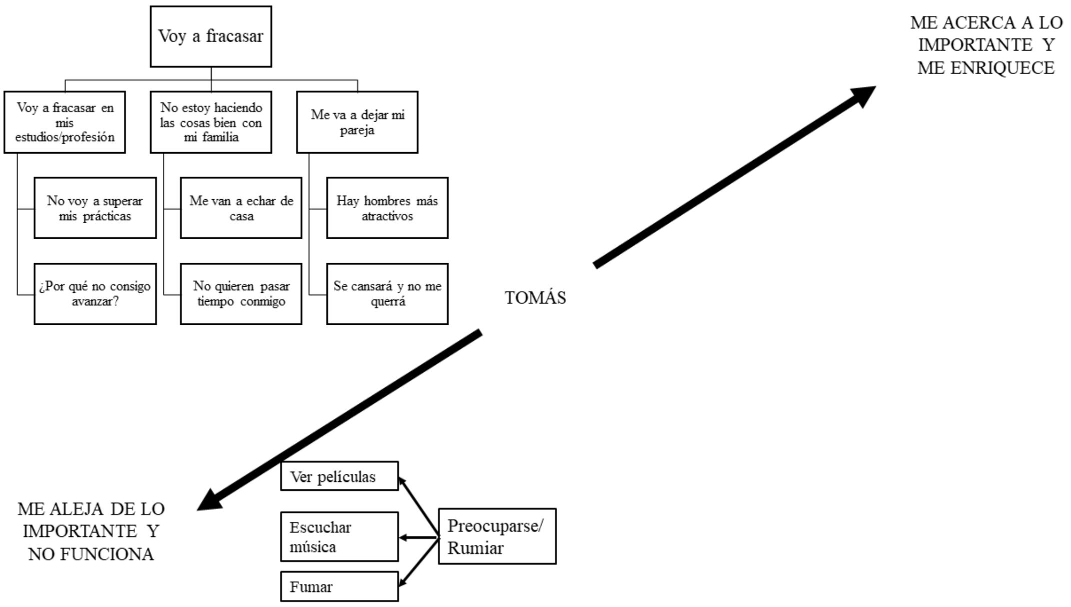

6. **Contactar con las consecuencias de la preocupación/rumia y las restantes estrategias de evitación experiencial.**

"Déjame que intente hacer un resumen de lo que hemos venido hablando para ver si he entendido bien tu experiencia. Hay veces en el día en que a Tomás le aparecen estos pensamientos (se señalan los pensamientos rápidamente señalando cuál es el "papá" de acuerdo a la jerarquía construida). Cuando este sucede, a Tomás, como es lógico, no le gusta, se siente mal y empieza a darle vueltas a la cabeza durante X tiempo. Durante ese tiempo, Tomás no hace lo que es importante para él y enriquecería su vida; más bien, lo que hace Tomás está controlado por su "papá" y sus "hijitos", como si éstos mandaran en su vida y Tomás fuera un esclavo de ellos. Otras veces, Tomás intenta dejar de pensar, de

enredarse, de preocuparse, de darle vueltas a su cabeza y para eso hace _____ (se señalan las principales estrategias de evitación experiencial). Incluso, otras veces Tomás hace cosas para intentar que el "papá" o los "hijitos" no aparezcan, que se queden callados, etc."

Una vez que el cliente constata que ésa es su experiencia, el terapeuta intenta ampliar la "radiografía del patrón inflexible":

- Explorar cuánto tiempo lleva el cliente con el patrón inflexible y si el "grande" está cada vez más presente. Ejemplos: "¿Cuánto tiempo lleva Tomás enredado con todos estos pensamientos y tratando de hacer cosas para no pensar en ellos? ¿Sientes que durante este tiempo el "papá" está más fuerte y tiene más "hijitos"? ¿Sientes que el "papá" está haciéndose más grande o más pequeño? Según tu experiencia, si las cosas siguen así, ¿algún día perderá fuerza el "papá" de tus miedos?
- Explorar las consecuencias del patrón inflexible en relación con las áreas valiosas. Ejemplos: "¿Cómo están las cosas que son importantes en la vida de Tomás? ¿Está Tomás dedicándoles el tiempo que se merecen? Si echáramos la vista atrás, ¿ha ido mejorando Tomás en las cosas que son importantes para él conforme el "papá" estaba más presente en su vida?

7. **Introducción de una metáfora física que resume y amplifica las consecuencias del patrón de evitación experiencial y propone una alternativa basada en discriminar el proceso de preocupación/rumia, tomar perspectiva y contactar con direcciones y acciones valiosas.**

"Te propongo que hagamos un pequeño ejercicio que puede ser parecido a lo que ocurre cuando vienen el "papá" o los "hijitos" y les das vueltas y vueltas y terminas haciendo cosas para que se vayan, pero que no son útiles en tu vida. Voy a escribir en este papel el "papá". Imagina que en esta otra hoja de papel estás haciendo un trabajo o un informe… Algo que es importante para ti en tu día a día…" (dependiendo de las características del cliente, se ajusta la tarea; lo importante es que el cliente perciba que esa actividad sería realmente importante y es lo que mejor podría hacer en ese momento para ir hacia arriba-derecha).
Después de un minuto en el que el cliente está concentrado en su tarea, el terapeuta pone bruscamente el papel con el "papá" delante de la cara del cliente.
"Pero, en un momento determinado aparece el "papá" y se ponen en frente de tu vista… Y parece que no te gusta mirarlo, ¿verdad?" (por lo general, los clientes se quedan paralizados mirando el papel y dejan de hacer la tarea).

"Entonces, me da la sensación de que intentas no ver ese papel y tratas de quitarlo empujándolo con la mano hacia delante… Inténtalo… Inténtalo con más fuerza…"

(el cliente empuja el papel, pero el terapeuta ofrece resistencia con su brazo de modo que el cliente comienza a experimentar una gran lucha que no fructifica para quitarse el pensamiento).

"Fíjate en lo que ocurre. ¿Qué pasó con el informe/trabajo que estabas realizando?" "¿Cuánto esfuerzo pusiste para quitar al papá? ¿Qué conseguiste empujando?

"El papá o los hijos eventualmente se van y Tomás se pone de nuevo a escribir… Pero me da la sensación de que después de un rato… ¡Otra vez aparecen!" (el terapeuta vuelve a poner el papel delante de la cara del cliente). "Y entonces… ¿Qué haces? Otra vez vuelves a empujar, ¿verdad?"… Y tras un buen rato, quizás se van, pero al rato… ¡Otra vez!... Y otra vez a empujar. Hazlo. Fíjate, ¿cómo te sientes después de tanto empujar? ¿Sueles sentirte así al final del día: agotado, tenso, con dolor en los hombros, cuello, brazos? (por lo general, los clientes suelen sentir tensión en el cuello, hombro y espalda fruto del estrés que provocar preocuparse/rumiar, por lo que establecer esta similitud suele ser útil para contacten aún mejor con la metáfora). Además, cuando viene el papá o los hijos, ¿cuántas cosas importantes para ti puedes hacer mientras luchas contra ellos?"

¿Se parece esta lucha a lo que te pasa a lo largo de los días? ¿Se parece esta lucha contra el papelito a cómo es tu vida?

Fíjate en una cosa, ¿qué ocurre cuando aparecen y empiezas a empujar? ¿Cuánta fuerza tienen cuando empiezas a empujar? ¿Se pone el papelito más o menos fuerte cuando empujas? A lo largo de este tiempo, ¿tienes la sensación de que el "papá" se ha hecho más fuerte y que han ido saliendo cada vez más "hijitos"?

Imagínate que tuvieras unos binoculares y pudieras verte a ti mismo dentro de un año mientras sigues empujando cada vez que aparece uno de estos pensamientos. ¿Cómo ves tu vida haciendo esto durante un año más? ¿Cómo te ves a ti mismo? (¿Feliz? ¿Cansado? ¿Con canas?...). Imagínate dentro de cinco años más… ¿Cómo estarías? ¿Te gusta cómo ves tu vida? ¿Está mejor o peor que ahora?… Imagínate que esto que estamos viendo fuera una película. ¿Qué título le pondrías?

Sin embargo, déjame que miremos una cosa… Hemos visto que cuando el "papá" o los "hijos" aparecen, les das más fuerza empujándolos… Si empujándolos sólo consigues darles más fuerza, ¿qué se te ocurre que podrías hacer? (Se moldea la respuesta hasta que el participante vea que una opción es dejar de empujar, dejar que el "papá" contacte poniendo la mano en una posición en la que le permita seguir con el trabajo/informe. Tras esto, ponemos al participante a escribir y cada poco tiempo van llegando pensamientos y él deja que simplemente le toquen y sigue escribiendo).

“Dejando que el papá o los hijos te toquen y sin empujarlos, ¿puedes hacer cosas que van en la dirección de enriquecer tu vida, es decir, hacia la arriba-derecha?... Imagina que aprendes a dejar que los pensamientos te toquen sin tener que estar empujándolos y coge de nuevo los binoculares… ¿Cómo te verías a ti mismo dentro de 1 año?... ¿Y dentro de 5 años?... ¿Quién estaría mandando en la vida de Tomás? ¿Tomás o el “papá” de Tomás y sus “hijitos”? ¿Cómo se llamaría en este caso la película?

8. **Práctica con un ejercicio físico en la discriminación del proceso de preocupación/rumia y toma de perspectiva.**

“Ahora, si te parece bien, te propongo hacer un ejercicio que puede ayudarte a ver la diferencia entre empujar y dejar de empujar los pensamientos, de modo que te sea más fácil hacerlo en tu vida diaria. La idea del ejercicio es que veas cómo es el proceso de enredarse con tus pensamientos y cómo podrías hacer para dejar de dar vueltas. El ejercicio puede resultar un poco curioso, quizás te parezca que estamos haciendo un poco el tonto, pero lo vamos a hacer juntos y creo que puede ser de gran ayuda. ¿Estás dispuesto?”

“Lo que vamos a hacer es una simulación de cómo te enredas y qué podrías hacer para desenredarte. ¿Has tenido alguna vez la experiencia de estar leyendo durante un buen rato y después darte cuenta de que no te has enterado de nada porque estabas pensando en otras cosas?”

“Lo que vamos a hacer es algo parecido a esto. Te invito a que empieces a leer este libro (se le pone un libro abierto en el inicio de un capítulo) y después de un poco voy a mostrarte uno de los pensamientos. Ante esto, en primer lugar, te voy a pedir que trates de enredarte diciendo los pensamientos que te vendrían a la cabeza. En ese caso, tienes que levantarte de la silla, ponerla en el centro de la habitación y darle una vuelta. Ese darle una vuelta es parecido a pensar sobre el pensamiento que acaba de venir y cuando piensas en el pensamiento, otro pensamiento relacionado viene… Y entonces, como estás eligiendo enredarte, das otra vuelta y viene otro pensamiento relacionado… ¿OK? La idea del ejercicio, como te comentaba, es que veas de qué está hecho el enredo. ¿Listo?”

El cliente se pone a leer. A los 15 segundos, se pone el papel en el que se había escrito el “papá” encima del libro y se le dice: “Aquí tienes al “papá”, ¿qué eliges hacer?”

El cliente se levanta y se le da el post-it con el “papá”, se le pide que se lo ponga delante de la cara y empiece a darle vueltas con las manos a la vez que él va dando vueltas alrededor de la silla. Cuando el cliente haya completado una vuelta, se le pregunta: ¿Y ahora qué te vendría a la cabeza o después de este pensamiento, qué otro te viene? Cuando el participante dice el siguiente pensamiento, se le da otro post-it y se le dice: “Y ahora,

Tomás puede elegir qué quiere hacer con su pensamiento. ¿Qué elige hacer?” El cliente comienza a darle vueltas y se pide que note cómo ha sido él el que ha elegido seguir dando vueltas: “Fíjate en cómo Tomás es la persona que ha elegido seguir dando vueltas a sus pensamientos”.

Se repite el proceso con 10 pensamientos.

Cuando termina la parte anterior del ejercicio, se le dice: “¿Cómo te has sentido dando vueltas a tus pensamientos? ¿Estás cansado? ¿Ha sido algo parecido a lo que ocurre en la vida real? ¿Cuánto has podido avanzar en la lectura mientras dabas vueltas a tus pensamientos?”

“Tomás, fíjate en una cosa… Cuando elegiste darle vueltas al “papá”, enseguida apareció otro pensamiento que no te gusta, ¿verdad? Y, en ese momento, elegiste darle vueltas a ese pensamiento, ¿y qué ocurrió? Vino otro… Y elegiste darle vueltas a otro y… Vino el siguiente… ¿Cuánto tiempo podría durar este proceso? ¿Cómo terminarías?”

“Lo curioso es que realmente Tomás pudo elegir a cada pensamiento qué hacer con él. En la parte anterior del ejercicio elegiste siempre darle vueltas, pero ahora vamos a hacer todo lo contrario. Vas a leer el libro y de vez en cuando voy a poner algunos papeles en medio, como antes. En este caso, lo que te propongo que hagas es que simplemente mires el papel y gentilmente lo pongas al lado, seas consciente de que has elegido ponerlo ahí y sigas leyendo… Después de otro rato, volveré a hacer lo mismo y te invito a que elijas dejarlo al lado gentilmente, darte cuenta de que Tomás puede elegir y seguir con la lectura”.

Se presentan 10 pensamientos.

“¿Cómo te has sentido durante el ejercicio? ¿Has podido seguir leyendo? ¿De qué trataba la lectura?... Fíjate en la gran diferencia entre empujar los pensamientos y darle vueltas a la silla o elegir no luchar con ellos, dejarles hueco y dedicarse a hacer lo que puede enriquecer tu vida…

Durante la próxima semana, te invito a que practiques dejando de empujar y darle vueltas a al “papá” y los “hijitos”. Es posible que haya veces en las que, sin darte cuenta, te descubras a ti mismo enredándote. No pasa nada, recuerda que a cada momento puedes elegir si les das vueltas al pensamiento que estás teniendo o lo dejas estar. Habrá días en los que incluso te enfades contigo mismo por haberte enredado. Fíjate que hasta podrías elegir enredarte en por qué te has enredado… Simplemente, te sugiero que no le des más vueltas y te pongas a hacer algo que sea importante para ti.

AUDIO 1

Este audio tiene como objetivo ayudarle a desarrollar la habilidad de darse cuenta de la diferencia entre enredarse y no enredarse en los pensamientos y aprender a dejarlos estar eligiendo hacer lo que es mejor para usted…

A continuación, le proponemos hacer algunos ejercicios para permitirse notar las sensaciones y pensamientos. Si lo desea, puede cerrar los ojos para concentrarse solamente en mi voz… Si en algún momento se distrae con algún pensamiento, no se moleste con él, simplemente dele la bienvenida y elija volver a escuchar mi voz aquí y ahora…

Comience eligiendo prestar atención a su respiración. Sencillamente, note cómo inhala aire y cómo, a continuación, lo exhala… No trate de alterar el ritmo de su respiración, simplemente note las sensaciones que le produce hacerse consciente de su respiración… Dese cuenta de que usted está eligiendo aquí y ahora centrarse en notar su respiración. Note cómo la respiración es una parte de usted aunque normalmente no le preste mucha atención. Siga notando las sensaciones que le produce respirar durante unos segundos (pausa de 15 segundos).

Ahora quisiera invitarle a pensar durante un minuto en qué cosas son importantes para usted durante el día de hoy o durante las próximas 24 horas… Si lo desea, pueda hacer una lista en papel o puede simplemente hacerla en su cabeza. Elija por un momento pensar en qué cosas puede hacer hoy que pueden enriquecer su vida (pausa de 30 segundos).

Bien, ahora trate de imaginarse haciendo una de esas acciones que ha pensado que pueden enriquecer su vida. Puede elegir la que quiera, pero le recomendamos que se imagine aquella en la que cree que tendrá más dificultades porque seguramente su mente le arroje pensamientos, recuerdos o sensaciones con los que usted puede quedarse enredado…

Permítase imaginar la situación en la que haría dicha acción que supondría enriquecer su vida… ¿Dónde es? ... ¿Hay más gente alrededor? … ¿Qué estaría pasando? … Permítase experimentar por un momento el pensamiento que puede aparecer en ese momento que podría impedir que usted hiciera aquello que es importante para usted…

A continuación, le proponemos que, ante este pensamiento, haga un esfuerzo por enredarse y darle vueltas a la cabeza. Note cómo elige enredarse aquí y ahora y vea qué otro pensamiento le viene a la mente… Note que ese pensamiento es algo suyo y que ante él puede elegir enredarse… Sea consciente de que elige enredarse y deje paso al siguiente pensamiento… Fíjese por un momento en cuál le viene… Y dese cuenta de que ante él puede elegir seguir enredándose… Enrédese y vaya a por el siguiente pensamiento que significaría seguir dándole vueltas a la cabeza… Note por un momento cómo seguramente

perdió el contacto con lo que iba a hacer… Y enrédese con el siguiente pensamiento que venga… Nótelo de nuevo como una parte de usted y elija de nuevo enredarse… Fíjese en lo que pasaría si siguiera eligiendo enredarse en los pensamientos, si siguiera empujándolos… ¿Cuánto tiempo pasaría enredado? ¿Estaría orgulloso al final del día con el tiempo gastado en empujar sus propios pensamientos?

Imagine de nuevo la situación en la que haría la acción que comentó… Note el pensamiento que podría venirle a la cabeza y elija si quiere luchar con él o, por el contrario, ponerlo en un globo y simplemente observarlo, dejarlo flotar libremente en el aire… No trate de modificarlo, sea cual sea el pensamiento, está bien… Simplemente dese cuenta de que usted es la persona que está notando ese pensamiento, y que usted puede elegir cuánto peso quiere que ese pensamiento tenga en su vida…

Nótese eligiendo realizar la acción que tenía en mente… Es probable que mientras la está realizando le venga otro pensamiento con el que podría enredarse… Permítase notarlo y, de nuevo, elija si quiere luchar con él o prefiere ponerlo en otro globo y dejarlo flotar libremente en el aire… Nótese a sí mismo eligiendo hacer esto y retomando rápidamente la actividad que realizaba…

Recuerde que a lo largo del día le vendrán montones y montones de pensamientos, unos pueden ser agradables, otros neutros y otros desagradables… Recuerde que sean como sean, usted puede elegir qué peso quieren que tengan en su día… Como siempre, la cuestión es: ¿qué elige hacer con su pensamiento: enredarse o dejarlo pasar mientras se centra en lo importante para usted?

Hemos terminado el ejercicio. Si así lo desea, puede practicar a solas con alguna otra situación en la que pueda enredarse durante el día de hoy. En cualquier caso, recuerde que lo importante es desarrollar la habilidad y que ésta, como la habilidad de montar en bicicleta, sólo se desarrolla haciendo las cosas que son importantes para usted, notando cualquier pensamiento que pueda aparecer en el camino sin empezar a luchar con él. Recuerde también que no pasa nada si en algún momento se enreda con sus pensamientos. Usted siempre puede empezar a elegir notarlos, elegir dejar de luchar y comenzar a hacer aquello que sea importante en ese momento.

SESIÓN 2

1. Ejercicio inicial de defusion

“Para comenzar esta segunda sesión te invito a que hagamos un breve ejercicio con los ojos cerrados para recordar algunas de las que vimos en la primera sesión y seguir practicando dejar estar los pensamientos y emociones sin luchar con ellos, empujarlos o darles vueltas. Para hacer el ejercicio es mejor que cierres los ojos para que puedas concentrarte mejor”.

Me gustaría que te centrases en tu respiración… Trata de notar cómo el aire entra y sale de tu nariz… Si en algún momento notas que te distraes porque vienen pensamientos o alguna preocupación a tu mente, simplemente nótalo, date cuenta de que son pensamientos que TÚ estás teniendo, dales la bienvenida y elige centrarte en el ejercicio que estamos haciendo…
Permítete hacer una respiración profunda y nota cómo se hincha tu pecho y tu vientre…
Nota el ritmo de tu respiración sin intentar alterarlo, tu único objetivo en este momento es observarlo…
Ahora, céntrate en cómo es el aire que entra por tu nariz. ¿Es frío o caliente?… Mantente ahí notando cómo ese aire frío entra y cómo luego el aire caliente sale…
Nota cómo ese aire entra como un globo hinchándose en tu pecho y tu vientre y sale desinflándose después… Nota cómo esa sensación va y viene y sencillamente estás aquí y ahora eligiendo notarla sin hacer nada con ella… Simplemente notando que es una sensación que TÚ tienes en este momento…
Responde para ti a las siguientes preguntas: ¿Quién está respirando?... ¿Quién está sintiendo esa sensación, ese globo que se infla y desinfla? ... ¿Quién está eligiendo aquí y ahora observar esa sensación? … ¿Quién está detrás de todo esto que estás sintiendo?…
OK, dejemos 10 segundo para que te veas ahí, eligiendo centrarte en cómo coges aire y lo sueltas.

Ahora te invito a que elijas centrarte en los sonidos que podemos escuchar alrededor…
Nota que puedes elegir hacer cualquier otra cosa, qué TÚ tienes capacidad para elegir qué quieres hacer… Y nótate eligiendo escuchar los sonidos de alrededor. Céntrate en los sonidos que provienen del pasillo… Si notas sonidos de otra parte, no pasa nada, dale la bienvenida a esos sonidos y nótate eligiendo centrarte en los sonidos del pasillo…
Dime, ¿quién está escuchando esos sonidos, ese ruido? …

Ahora te invito a contemplar los pensamientos que te vienen a la cabeza… Me gustaría que notaras que la mente no deja de arrojar pensamientos, uno tras u otro… A lo que me gustaría invitarte aquí y ahora es a notar esos pensamientos que llegan, a simplemente observarlos, contemplarlos, como si estuvieras viendo un paisaje que te gusta o un

cuadro… Simplemente lo observas sin tratar de modificarlo, sin hacer fuerza contra el pensamiento…

Nota qué pensamiento te visita ahora mismo. Cualquier cosa que venga a tu mente está bien… ¿Ya? Ahora, imagina que tienes un globo entre las manos… Imagina que escribes en su superficie el pensamiento que estás teniendo en este momento… ¿Ya? … Simplemente, nota que el pensamiento está ahí, en el globo, y contémplalo… Permítete elegir no luchar con él… No discutir con él… No empujarlo… Date cuenta de que estás capacitado para simplemente observar ese pensamiento escrito… Dime, ¿quién está observando ese pensamiento ahí?…
Mira cómo, si quisieras, podrías elegir discutir con ese pensamiento, pensar en por qué lo has tenido o tratar de empujarlo, pero simplemente estás aquí y ahora eligiendo voluntariamente observarlo sin hacer nada con él… Te invito a que simplemente elijas soltarlo y dejarlo flotar libremente en el aire.

Nota que ahora puedes elegir entre quedarte centrado en este pensamiento o dejar que otro aparezca en tu mente… ¿Qué te parece si eliges dejar pasar el pensamiento del globo y te permites notar qué pensamiento viene ahora a tu mente? …
Puede ser cualquiera, simplemente nota qué pensamiento viene… ¿Ya? ¿Qué pensamiento te vino?… Nota que puedes elegir poner ese pensamiento en el globo…
Nota que ahora el pensamiento está en el globo y que tú estás eligiendo simplemente observarlo, no enfrentarte a él…
Date cuenta de quién está teniendo ese pensamiento, de quién está eligiendo qué hacer con él y de quién es capaz de observar todo eso y responder esta pregunta…
Ahora, simplemente permítete soltar el globo y dejarlo flotar libremente en el aire…

Ahora, fíjate en qué otro pensamiento viene a tu mente… Simplemente nótalo y escribe una palabra que lo resuma en el globo. Nota quién ha elegido la palabra que lo resumía y nota quién está eligiendo aquí y ahora contemplar ese pensamiento sin oponerse a él…

¿Qué eliges hacer ahora? ¿Quedarte centrado en ese pensamiento o dejar paso a otro para seguir practicando? … OK, nótate tomando esa elección y mira qué otro pensamiento aparece en tu mente… Simplemente, escribe una palabra que lo resuma en el globo… Nota quién ha puesto ese pensamiento ahí y quién está eligiendo observarlo sin hacer nada con él, sin entrar a juzgar si es bueno o malo.

2. **Repaso de la experiencia tras la primera sesión (5 minutos)**

“¿Has tenido la experiencia de notar algunos “papás” e “hijos” y no enredarte con ellos al estilo de lo que acabamos de hacer? ¿Te has notado enredado en sus pensamientos? ¿Ha sido como siempre o has notado que te has desenganchado antes de lo normal? ¿Has realizado cosas distintas que pueden enriquecer su vida?

El objetivo de esta sesión es trabajar en desarrollar la habilidad de dejar estar los pensamientos y no enredarse empujándolos o dándoles vueltas. Para ello, te voy a proponer hacer varios ejercicios para practicar. ¿Te parece bien?

3. **Ejercicio de asociación libre**

"Ahora, te invito a realizar un sencillo ejercicio para dejar estar los pensamientos y no enredarnos con ellos. El objetivo del ejercicio es que contemples qué pensamientos, recuerdos o imágenes vienen a tu mente en reacción a algunas palabras que voy a ir diciéndote. Es decir, lo único que tienes que hacer en este ejercicio es notar qué es lo primero que viene a tu mente de manera espontánea y darte cuenta de cómo puedes elegir simplemente dejar eso estar o puedes darle vueltas al pensamiento intentando comprenderlo o empujarlo. Si vienen más cosas a tu mente después de la primera, simplemente date de que son otros pensamientos que te han visitado y que puedes elegir si les dejas estar o los empujas y les das más fuerza. ¿Listo?"

Mango… (7 segundos)
Libro… (7 segundos)
Vacaciones… (7 segundos)
Nombre de la persona (p.ej., Tomás)… (7 segundos)
Casa… (7 segundos)
Estudio… (7 segundos)
Miedo… (7 segundos)
Margaritas… (7 segundos)
Justicia… (7 segundos)
Preocupación… (7 segundos)
Sueño… (7 segundos)
Enredo… (7 segundos)

"¿Qué tal? ¿Cómo ha sido la experiencia? ¿Has podido notar cómo venían pensamientos e imágenes a tu cabeza? ¿Te has notado dándote cuenta de que son algo tuyo momentáneo? ¿Te has notado eligiendo dejarlos estar sin darles vueltas?"

4. **Ejercicio de fantasear y preocuparse haciéndose consciente.**

"En el siguiente ejercicio te propongo, en primer lugar, que fantasees con algo agradable (por ejemplo: estar en la playa o viendo tu paisaje favorito) durante un par de minutos. Puedes elegir hacer el ejercicio con los ojos abiertos o cerrados. Cada cierto tiempo, yo haré un sonido con mi mano en la mesa como señal para que te hagas consciente de que tú eres quien está eligiendo conscientemente fantasear, para darte cuenta de que esa

fantasía la has creado tú y para ver que sólo tú puedes elegir si sigues con ella o la dejas pasar".

SE DEJAN 2 MINUTOS Y SE HACE UN SONIDO EN LA MESA CADA 20 SEGUNDOS (6 VECES A LO LARGO DEL PERIODO).

"Ahora te propongo que hagas lo mismo con una preocupación importante que tuvieras durante la semana pasada. Simplemente, intenta preocuparte y cada cierto tiempo haré el mismo sonido para que recuerdes que tú eres quien está eligiendo preocuparse y que podrías parar si así lo eligieras".

SE DEJAN 2 MINUTOS Y SE HACE UN SONIDO EN LA MESA CADA 20 SEGUNDOS (6 VECES A LO LARGO DEL PERIODO).

"¿Cómo te ha ido? ¿Te has hecho consciente de cómo estabas fantaseando o preocupándote? ¿Es algo distinto a lo que suele ocurrir en tu vida?"

5. **Ejercicio del contacto visual.**

"Ahora te invito a hacer un ejercicio un poco más complicado, pero que puede ser muy útil para seguir practicando. Te invito a que durante unos minutos nos miremos a los ojos y simplemente notemos a cada instante qué pensamiento y sensación viene. Es probable que durante este tiempo notemos incomodidad, vergüenza, etc. Lo único que tenemos que hacer, tú y yo, es notar esos pensamientos y sensaciones dejándolos estar y elegir seguir manteniendo la mirada fija. Considera que mantener la mirada es lo más importante que puedes hacer en este momento para acercarte a lo que es más importante para ti. Siendo así, ¿estás dispuesto a hacer el ejercicio? … Nota quién acaba de tomar esa elección… Comencemos…"

SE REALIZA EL EJERCICIO DURANTE APROXIMADAMENTE 2 MINUTOS. SU EL PARTICIPANTE SE DISTRAE, EL TERAPEUTA LE PIDE GENTILMENTE QUE NOTE EL PENSAMIENTO O SENSACIÓN QUE LO DISTRAJO Y ELIJA VOLVER A CENTRARSE EN LA TAREA.

Al terminar, se revisa la experiencia del participante: ¿fue capaz de notar los pensamientos que venían y seguir con la tarea?

6. **Ejercicio de contar hacia atrás de 3 en 3**

"A continuación, vamos a hacer un sencillo ejercicio de aritmética, pero vamos a poner pesas para poder ejercitar. Te invito a que cuentes hacia atrás desde 100 de 3 en 3. Es

decir: 100, 97, 94, 91... En el proceso, yo voy a decir algunos de los pensamientos que suelen molestarte y con los que sueles recordar. Tu objetivo es simplemente notar que vinieron, dejarlos estar y elegir seguir contando…"
El objetivo de este ejercicio es poner una tarea aritmética que sea complicada para el participante de modo que surjan dudas durante la tarea sobre si se está haciendo bien la tarea o no, el participante se autocorrija, etc. El terapeuta simplemente debe señalar al participante que da igual si lo está haciendo bien o mal, que el único objetivo es seguir haciendo el ejercicio hasta llegar a cero.

7. **Escribir con la mano no dominante**

"Ahora, te invito a hacer el siguiente ejercicio. Como el otro día, imagínate que tienes que escribir un informe muy importante. Este informe es un paso de gigante para ir en dirección de lo que quieres en la vida. Sin embargo, tienes que escribirlo con tu mano no dominante… El objetivo del ejercicio es que notes la sensación de dificultad, cualquier pensamiento que te venga a la cabeza mientras escribes que no esté relacionado con el informe y lo dejes estar… Simplemente, nota que no es fácil, nota la incomodidad… Nota que pasar esa incomodidad y dificultad forma parte de hacer algo que es muy importante para ti y elige seguir intentando escribir sin darle vueltas a que es más o menos difícil".

Al finalizar el ejercicio, debe revisarse la experiencia del participante enfatizando cómo, a pesar de la incomodidad y la sensación de que no lo estaba escribiendo de manera clara, eligió seguir escribiendo.

8. **Ejercicio de suprimir versus dejar estar**

"Ahora, te invito a hacer un pequeño experimento sobre algo que suele pasarnos a veces. En ocasiones no queremos pensar en algo y, como veíamos el otro día, empezamos a empujarlo para que desaparezca. Veamos qué ocurre cuando intentamos sacar pensamientos de nuestra cabeza. Cierra los ojos e imagina un elefante rosa… ¿Lo tienes? Ahora, imagínate que para hacer las cosas más importantes en tu vida tienes que, por todos los medios, no tener ese pensamiento o imagen. Imagínate que para hacer esas cosas que son tan importantes para tienes que salir de esta sala, pero resulta que no puedes salir de la sala mientras tengas en la cabeza la imagen del elefante rosa… Por tanto, intenta borrarla de tu mente porque si la imagen está, aunque sólo sea de fondo, no podrás salir de la sala".

Se dejan 2 minutos para que pruebe a hacerlo…

"¿Qué tal? ¿Podrías alguna vez pasar por la puerta y dirigirte a lo que es importante para ti si tuvieras que eliminar por completo la imagen del elefante rosa?"

“Ahora deja que venga a tu mente uno de los pensamientos de los que hablamos el otro día… ¿Ya? Intenta hacer lo mismo… Intenta por todos los medios dejar de pensar en él, de expulsarlo de tu mente antes de poder ir hacia la puerta…”

Se deja 1 minuto…

“¿Se parece esto a lo que ocurre a veces: que no te implicas en hacer las cosas que son importantes para ti porque estás luchando por no pensar determinadas cosas?”

“Intenta ahora ir con ese pensamiento en la cabeza, deja que venga o se vaya libremente, mientras tú te diriges hacia la puerta y la abres para ir en la dirección que quieres en tu vida”.

El participante se levanta y va hacia la puerta notando el pensamiento…

“¿Pudiste hacerlo? ¿Qué tal si en lugar de tratar de expulsar al “papá” y los “hijos”, los dejas estar mientras te dedicas a abrir las puertas de tu vida?”

9. **Exploración de acciones valiosas a realizar durante las siguientes semanas y especificación de barreras (5 a 10 minutos)**

“Si te parece bien, te propongo que hablemos ahora de las puertas a las que puedes tocar e intentar abrir durante la próxima semana; es decir, esas acciones, por pequeñas que sean, que podrían suponer un cambio en tu vida. Esas acciones que realizarías y que al final del día te sentirías orgulloso de haberlas hecho porque significa algo importante para ti.

¿Qué cosas serían? (Se explora en distintas áreas o facetas de la persona)

¿Qué acciones concretas tendrías que hacer? ¿Dónde? ¿Cuándo?…

Teniendo en cuenta lo importante que sería realizar estas acciones, ¿qué podría impedírtelo? ¿Qué “hijos” podrían interponerse entre Tomás y hacer las cosas que son importantes para Tomás? ¿Qué podría hacer Tomás cuando estos pensamientos y/o emociones llegaran? ¿Podrías aplicar los ejercicios que hemos hecho: es decir, dejar estar esos pensamientos y/o emociones sin luchar con ellos y centrarte en hacer aquello que es importante?”

10. **Cierre e introducción de ejercicios en formato audio y compromiso de realizarlos**

“Durante la próxima semana te propongo que practiques con unos archivos de audio. En ellos vas a tener ejercicios parecidos a los que hemos hecho hoy. Es importante que

practiques diariamente con los audios y que vayas cambiando de unos a otros para que la práctica sea lo más variada posible. No se trata de que seas experto en un ejercicio en particular, si no en que tengas la práctica más variada posible porque, cuanto más variada, mayor efecto ésta puede tener. Recuerda que la idea es desarrollar la habilidad de dejar estar al "papá" y a los "hijos", notar la diferencia entre enredarse y no enredarse, y ver cómo a cada paso puedes elegir centrarte en algo que sea más relevante para tu vida que en dar vueltas improductivas en tu cabeza.

AUDIO 2

En este audio le invito a realizar un sencillo ejercicio con el objetivo de notar qué pensamientos vienen y aprender a dejarlos estar sin empujarlos o enredarse con ellos. Lo único que tienes que hacer en este ejercicio es contemplar qué pensamientos, recuerdos o imágenes vienen a tu mente al escuchar algunas palabras. Simplemente, nota que esos pensamientos son algo momentáneo que acaba de venir a tu mente y permítete notar que puedes elegir simplemente observarlos y seguir centrado en el ejercicio. Si en algún momento te distraes, no pasa nada, simplemente nota cómo tu mente te llevó en otro camino, sé amable con ella y vuelve a concentrar en el ejercicio.

Vamos a comenzar, simplemente concéntrate en mi voz y nota qué viene a tu mente con las siguientes palabras:

Papaya… (7 segundos de pausa)
Computador… (7 segundos de pausa)
Estudio… (7 segundos de pausa)
Tristeza… (7 segundos de pausa)
Juventud… (7 segundos de pausa)
Ansiedad… (7 segundos de pausa)
Fracaso… (7 segundos de pausa)
Calor… (7 segundos de pausa)
Preocupación… (7 segundos de pausa)
Dolor… (7 segundos de pausa)
Cine… (7 segundos de pausa)
Enredo… (7 segundos de pausa)
Ensalada… (7 segundos de pausa)

Hemos terminado el ejercicio. Por un momento, piensa en esta experiencia. Nota cómo venían los pensamientos e imágenes a tu mente. Date cuenta de cómo cada uno de los que has tenido en estos minutos es un pensamiento momentáneo y que ante cualquier de ellos, puedes elegir si le das fuerza o lo dejas estar.

<u>AUDIO 3</u>

El siguiente ejercicio tiene como objetivo hacerte consciente de tu proceso de pensamiento y de cómo a veces éste transcurre en piloto automático. Para ello, te proponemos, en primer lugar, que fantasees con algo agradable durante un par de minutos (por ejemplo: estar en la playa o viendo tu paisaje o película favorita). Cada cierto tiempo, escucharás un sonido como éste… (Se hace un ruido como tocando una puerta, por ejemplo). El sonido anterior será una clave para hacerte consciente de que tú eres quien está eligiendo conscientemente fantasear, para darte cuenta de que esa fantasía la has creado tú y para ver que sólo tú puedes elegir si sigues con ella o la dejas pasar.

SE DEJAN 2 MINUTOS EN SILENCIO Y SE HACE UN SONIDO EN LA MESA CADA 15 SEGUNDOS.

Ahora te propongo que hagas lo mismo con una preocupación que tuvieras durante este último día o durante la semana pasada. Simplemente, intenta preocuparte y cada cierto tiempo oirás el mismo sonido para que recuerdes que tú eres quien está eligiendo preocuparse y que podrías parar si así lo eligieras.

SE DEJAN 2 MINUTOS EN SILENCIO Y SE HACE UN SONIDO EN LA MESA CADA 15 SEGUNDOS.

Hemos terminado. Te proponemos que trates de aplicar este ejercicio en distintos momentos del día en los que solemos ponernos en piloto automático. Por ejemplo, puedes utilizarlo mientras te duchas, almuerzas, caminas, etc. Simplemente, permítete notar cada cierto tiempo en qué estás pensando y experimentando en ese momento y elige si quieres seguir en piloto automático o simplemente concentrarte en lo que estás haciendo.

AUDIO 4

En este ejercicio te invitamos a cerrar los ojos y concentrarte en mi voz. Si en algún momento te das cuenta de que te estás distrayendo, simplemente nota qué pensamiento hizo que te distrajeras, déjalo estar y nótate eligiendo volver a concentrarte en mi voz.

Céntrate en tu respiración por unos momentos… Trata de notar cómo el aire entra y sale de tu nariz… Permítete hacer una respiración profunda y nota cómo se hincha tu pecho y tu vientre… Nota el ritmo de tu respiración sin intentar alterarlo, tu único objetivo en este momento es observarlo…

Nota cómo el aire entra como un globo hinchándose en tu pecho y tu vientre… Y sale desinflándose después… Nota cómo esa sensación va y viene y sencillamente estás eligiendo notarla sin hacer nada con ella… Simplemente nota que es una sensación que tú tienes en este momento y que no es necesario que trates de modificarla…

Ahora, permítete sentir las sensaciones de tu cuerpo. Nota si el lugar en el que estás sentado o acostado es suave o duro. Elige fijarte en la sensación que provoca en tu cuerpo sin tratar de alterarla. Centra tu atención en que tienes capacidad para simplemente hacerlo sin juzgar si la sensación es más agradable o desagradable…

Nota también en qué posición están tus brazos y tus manos y dónde están apoyados. Elige no juzgar si están en una postura adecuada o inadecuada. Simplemente, permítete ser consciente de dónde están y de las sensaciones que provocan sin intentar modificarlas.

Ahora te invito a contemplar los pensamientos que te vienen a la cabeza… Nota qué pensamiento te visita ahora mismo… Cualquier cosa que venga a tu mente está bien… Imagina que tienes un globo entre las manos y que escribes en su superficie el pensamiento que estás teniendo en este momento… Simplemente, nota que el pensamiento está en el globo y contémplalo… Permítete elegir no luchar con él… No discutir con él… No empujarlo… Date cuenta de que estás capacitado para simplemente observar ese pensamiento escrito… Te invito a que elijas soltarlo y dejarlo flotar libremente en el aire.

Nota que has dejado que el globo con tu pensamiento flote en el aire… Y mira qué otro pensamiento aparece en tu mente… Nota que puedes elegir poner ese pensamiento en el globo…

Mira cómo ahora el pensamiento está en el globo y tú estás eligiendo observarlo y no enfrentarte a él… Date cuenta de quién está teniendo ese pensamiento, de quién está eligiendo qué hacer con él y de quién es capaz de observar todo eso y responder esta

pregunta… Ahora, simplemente permítete soltar el globo y dejarlo flotar libremente en el aire…

Ahora, fíjate en qué otro pensamiento viene a tu mente… Simplemente nótalo y escribe una palabra que lo resuma en el globo. Nota quién ha elegido la palabra que lo resumía y nota quién está eligiendo aquí y ahora contemplar ese pensamiento sin oponerse a él…

¿Qué eliges hacer ahora? ¿Quedarte centrado en ese pensamiento o dejar paso a otro para seguir practicando? Nótate tomando esa elección y mira qué otro pensamiento aparece en tu mente… Simplemente, escribe una palabra que lo resuma en el globo… Nota quién ha puesto ese pensamiento ahí y quién está eligiendo observarlo sin hacer nada con él, sin entrar a juzgar si es bueno o malo.

Sigamos con el ejercicio, pero en lugar de centrarnos en el pensamiento que viene a tu mente, date permiso para pensar en los pensamientos que suelen llevarte a que comiences a darle vueltas a la cabeza. Tómate unos pocos segundos para imaginar que estás en un momento y situación reciente, quizás hoy, ayer o durante la semana pasada, en la que tuviste uno de esos pensamientos que te llevan a enredarte y a luchar con ellos...

Permítete recordar dónde estabas… ¿Había alguien más contigo?… ¿Qué comenzó a pasar que hizo que comenzaras a enredarte en tus pensamientos?… Fíjate en cuál parece que fue el pensamiento con el que comenzaste a enredarte… Fíjate en que puedes elegir poner ese pensamiento escrito en el globo y contemplarlo. ¿Eliges hacerlo o prefieres enredarte, pelearte o discutir con él? Nótate eligiendo dejarlo en el globo y contempla el pensamiento sin hacer nada más… Imagínate a ti mismo eligiendo hacer esto… ¿A dónde te llevaría hacerlo, qué cosas importantes para ti conseguirías realizar?… ¿Y si lo hicieras un año más? ... ¿Y cinco años?

Ahora te invito a imaginar otra situación o momento reciente en el que te has quedado enredado dándole vueltas a la cabeza… ¿Dónde estás?… ¿Cómo has llegado a ese sitio? … ¿Quién está ahí?… ¿Qué comienza a pasar?… Fíjate en cuál parece que fue el pensamiento con el que comenzaste a enredarte… En este momento, te invito a que pongas ese pensamiento en el globo para ver si esto te permite verlo sin reaccionar luchando ante él… Nótate eligiendo ponerlo en el globo. Contempla el pensamiento escrito en el globo sin hacer nada más… ¿Qué alternativa eliges? ¿Luchar con el pensamiento o hacerle hueco, dejarlo estar y centrarte en lo que es importante hacer en ese momento?…

Por último, me gustaría que te permitieras recordar por un momento todos los pensamientos que has tenido desde que empezamos a hacer este ejercicio… Date cuenta del montón de pensamientos que has tenido y que cada uno de ellos es tuyo.

Permítete por un momento pensar en todos los pensamientos y emociones que has tenido a lo largo de tu vida…

Y permítete pensar en todo los que tendrás en el futuro, desde este fin de semana hasta dentro de 10 o 20 años…

Date cuenta de que todos ellos son tuyos, de que son una parte momentánea de ti. Nota cómo, a lo largo de la vida, unos pensamientos han dado paso a otros, y unas emociones a otras…

Nota cómo todos los pensamientos y emociones son transitorios, momentáneos y que TÚ puedes elegir el peso que quieres darle. Puedes elegir darle peso luchando con ellos, discutiendo, tratando de quitarlos o escapar de ellos, o puedes simplemente soltarlos y dejar que estén ahí flotando en el aire, unas veces yendo y otras viniendo, mientras tú haces las cosas que son importantes para ti.

Nota finalmente que dejar pasar los pensamientos y emociones, dejarlos libres como hacías con los globos y enfocarse en lo que es importante es una habilidad que puede aprenderse, como aprendiste a montar en bicicleta. Si en algún momento te caes y te pones a luchar con los pensamientos y emociones, no pasa nada, has caído muchas otras veces, lo único que importa es tu determinación por aprender a dejarlos estar y hacer lo que puede enriquecer tu vida.

SESIÓN 3

1. Revisión del funcionamiento desde la última sesión (10 minutos).

Revisión de la utilidad de la sesión: ¿Qué tal te ha ido desde la sesión pasada? ¿Crees que te resultó útil la sesión? ¿En qué cosas lo notaste? ¿Qué cosas has recordado de ella?

Utilización de los audios: ¿Has utilizado los audios? ¿Cómo y cuántas veces? ¿Te ha resultado útil? ¿Para qué? (Si no lo utilizó, explorar por qué no lo hizo acríticamente y si cree que haciéndolo puede desarrollar mejor la habilidad). Explorar en qué momento podría hacerlo y planificar.

Acciones valiosas: ¿Cómo te ha ido con las acciones que apuntaste en la sesión pasada? ¿De qué te sientes orgulloso de haber hecho? ¿De qué no?

Disparadores y reacción: ¿Con qué jefes o jefecillos te has enredado estos días y esto te ha impedido cumplir con los compromisos que te pusiste a ti mismo? (Incluir "nuevos jefes" dentro de los otros si están relacionados jerárquicamente con ellos; es decir, si apareció durante este tiempo un "jefe" pero resulta que está contenido en uno de los explorados, señalar la jerarquía y cómo esos jefes tienen pensamientos "alguacilillos").
¿Te has visto empujando cuando llegaban los jefes? ¿Tienes la sensación de que le diste más fuerza al jefe cuando empujabas? ¿Era como si le dieras las riendas de tu vida durante un rato? ¿Cómo has empujado? ¿Qué hacías? ¿Hay cosas que solías hacer para empujar que has dejado de hacer? ¿Te has visto haciendo cosas nuevas?

2. Ejercicio "película de la vida" (30 minutos)

Hoy te propongo hacer un ejercicio. El objetivo de este ejercicio es que veamos mucho más de cerca la dirección hacia la derecha; es decir, que veamos qué cosas enorgullecerían a PEPE y qué cosas puede hacer en esa dirección. ¿Te parece bien?

Te invito a que te pongas cómodo y cierres los ojos. Durante un minuto, imagina que lo más importante en este momento es estar sintiendo cómo tus piernas y glúteos están apoyados sobre la silla… Nota si la silla está suave o dura… Date cuenta de que puedes elegir fijarte en la sensación que provoca estar sentado en la silla y de que eres tú quien está observando eso… Centra tu atención en que PEPE tiene capacidad para simplemente hacerlo sin juzgar si la sensación es más agradable o desagradable… Nota también en qué posición están tus brazos y tus manos y dónde están apoyados… Deja que PEPE elija no juzgar si están en una postura adecuada o inadecuada… Simplemente, permítete ser consciente de dónde están y de las sensaciones que provocan sin intentar modificarlas… Date cuenta de cómo también puedes elegir notar la temperatura de la habitación y si algunas partes de tu cuerpo están más frías o calientes que otras... Finalmente, nota cómo

PEPE es quien puede elegir centrarse en su respiración, en notar su ritmo y sin intentar modificarlo, dejarlo estar y simplemente darse cuenta de que es la única persona que puede observarlo…

En este ejercicio te invito a que hagamos una pequeña película autobiográfica. Imagina que estás sentado en una butaca de una sala de cine y que ahí se va a proyectar la película de tu vida, de tus sueños y tus decepciones, tus anhelos e inquietudes... Deja que aparezca en la pantalla una imagen de PEPE cuando era más joven… Permítete que venga una imagen sobre un momento en el que PEPE no luchaba contra sus jefes; es decir, contra sus propios pensamientos y emociones… Permítete mirar cuáles eran los sueños de PEPE en aquél entonces… (PAUSA 30 segundos). Permítete también mirar de qué cosas se sentía PEPE orgulloso de estar haciendo… (PAUSA 30 segundos)

Ahora date permiso para que en la pantalla se proyecten imágenes de cómo es tu vida en la actualidad. Simplemente, proyecta en la pantalla imágenes y escenas de las cosas que sueles hacer en aquellas áreas que son importantes para ti. Comencemos, si te parece bien, con tu familia y permítete notar qué cosas haces actualmente de las que el PEPE de hace años se sentiría orgulloso. Sencillamente, proyecta algunas imágenes relacionadas con ese orgullo en la pantalla y permítete ver cómo puedes observarlas desde tu butaca. Permite que se proyecten las imágenes y que éstas cobren vida propia sin intentar modificarlas. Simplemente sé la persona que se permite ver esas imágenes... (PAUSA 30 segundos)

Dime, ¿qué ves en la pantalla que te cause orgullo sobre lo que haces a día de hoy en relación con tu familia?... No importa si son grandes o pequeñas cosas, sólo permítete compartir conmigo qué ha aparecido en la pantalla…

Permítete ahora que vengan imágenes sobre lo que te hace sentir orgulloso de tu actuación en relación a tu trabajo, formación u ocupaciones… No trates de modificar las imágenes que vienen, sencillamente sé el observador de esas imágenes y nota cómo esas imágenes son una parte más de ti (PAUSA 30 segundos)

Dime, ¿qué ves en la pantalla que te cause orgullo sobre lo que haces a día de hoy en relación a tu trabajo, formación u ocupaciones?... No importa si son grandes o pequeñas cosas, sólo permítete compartir conmigo qué ha aparecido en la pantalla…

Permite ahora que aparezcan imágenes sobre las acciones que te hacen sentir orgulloso en relación con tus amistades y/o aficiones… Si te ves forzando la imagen, simplemente nota que puedes adoptar la posición de observador de esa película mental y deja que en la pantalla se proyecte aquello que aparezca en tu mente libremente (PAUSA 30 segundos)

Dime, ¿qué ves en la pantalla que te cause orgullo sobre lo que haces a día de hoy en relación con tus amistades y/o aficiones?...

Deja ahora que vengan imágenes sobre las acciones que te hacen sentir orgulloso en relación con tu autocuidado y salud, así como de cualquier área que para ti es importante y que no hemos mencionado antes… Permítete observar las imágenes que aparecen (PAUSA 30 segundos)

Dime, ¿qué ves en la pantalla que te cause orgullo sobre lo que haces a día de hoy en relación con tu salud y otras cosas que son importantes para ti?

Ahora te invito a mirar las escenas que aparezcan en la pantalla de tu mente en relación con las cosas que haces a día de hoy de las que no te sientes orgulloso, de las que el PEPE antiguo no se sentiría tampoco orgulloso. Esto puedes ser algo difícil, pero nótate dejando que las imágenes fluyan y permítete ser su observador (PAUSA 30 segundos).

Dime, ¿qué ves en la pantalla de lo que no te sientes orgulloso?

Date permiso ahora para imaginar o soñar cómo te gustaría que fuera tu vida dentro de 1 año… Permite que se proyecte en la pantalla qué estaría haciendo PEPE, lo que realmente soñaría si se lo permitiera... Si te notas modificando esas escenas porque te viene el impulso de rebajar tus aspiraciones para no sentirte mal si no las cumples, simplemente te invito a que notes que PEPE está teniendo ese impulso y que simplemente lo dejes estar sin enredarte con él (PAUSA 30 segundos).

Asimismo, durante este ejercicio te pido que me des permiso para que cuando vea que tu mente trata de sabotearte poniéndote peros, preocupaciones o autoevaluaciones, pongamos éstos en un globo y simplemente elijas dejarlos estar en él.

¿Cómo desearías que fuera tu vida dentro de 1 año? Tómate un minuto para ver las imágenes proyectadas en la pantalla y, cuando estés listo, cuéntame qué ves

Permite ahora que vengan imágenes de cómo desearías que fuera tu vida dentro de 5 años. Tómate unos minutos para ver la pantalla y cuando estés listo, cuéntame qué ves

Finalmente, permítete mirar cómo desearías que fuera tu vida dentro de 10, 15 o 20 años. Permítete mirar la pantalla sin modificar las imágenes y cuéntame qué ves cuando estés listo.

Ahora te invito a que retomes todo lo que hemos visto hasta ahora y te permitas sentir cómo de plena estaría tu vida, cómo te sentirías después de 10, 15 o 20 años haciendo esas cosas con las que te sentirías orgulloso en las distintas áreas que son importantes

para ti. Date cuenta de quién es la persona que está imaginando esto y de que sin esa persona esas imágenes no existirían, no tendrían sentido ni razón de ser.

Ahora te invito a que vengan unas cuantas imágenes de cómo sería tu vida si tú eligieras permitir que los jefes (miedos, recuerdos, preocupaciones, etc.) fueran los que mandaran en tu vida, si te enredaras cuando ellos vienen y dejaras de hacer lo que es realmente importante para ti. ¿Cómo estaría tu vida dentro de 1 año si eligieras continuamente que los jefes mandaran?

¿Cuáles serían los jefes que habrían estado mandando, esos jefes que son los que sostienen al resto de jefecillos que pueden ir apareciendo día a día?

Te invito a que, por un momento, los mires a la cara y te permitas notarlos y sentirlos. Nótate eligiendo ponerlos en un globo… Y nota cómo observándolos sin hacer nada con ellos, PEPE tiene la capacidad de elegir qué tipo de vida quiere tener: la que sueña o la que le ordenan tener los jefes. Nota cómo, además, al ser esos pensamientos del globo tuyos puedes elegir la fuerza que quieres que tengan.

¿Qué ocurriría, qué te pondrías a hacer si eligieras dejar que ese jefe mandara? ¿Qué otros jefes vendrían? Puedes poner cada uno de ellos en un globo… ¿Puedes notar cómo incluso aunque vinieran todos esos globos podrías seguir eligiendo qué quieres hacer: si mandar tú y seguir haciendo las cosas con las que te sentirías orgulloso y enriquecerían tu vida o dejar que manden ellos y tu vida se deteriore?… Permítete por un momento sentir cómo, a veces, esos jefes intentan embaucarte y te lanzan excusas para no hacer las cosas que son importantes para ti y mantenerte tranquilo, pero en una tranquilidad tensa y poco productiva… Nota también cómo, aunque pueda ser desagradable tener y dejar estar en el globo esos pensamientos, tú puedes elegir si dejas que ellos controlen tu vida y te enreden con sus excusas o prefieres elegir hacer lo que es importante para ti y estar cada día un poquito más cerca de la satisfacción que sentiste hace un rato cuando te permitiste ver cómo habría sido tu vida.

Hemos terminado…

3. **Resumen de la experiencia con el ejercicio anterior, metáfora del jardín y compromiso con acciones valiosas (15 minutos).**

Ahora te invito a que miremos a la parte derecha del esquema, es decir, a lo que realmente es importante para ti en la vida. El terapeuta resume la experiencia del ejercicio anterior y las cosas que parecen importantes para el paciente. A continuación, introduce la metáfora del jardín.

METÁFORA DEL JARDÍN Y EVALUACIÓN ESTADO VALORES: “Imagínate que vives en un mundo en el que te ha tocado ser jardinero y lo que más te importa en la vida es tu jardín. En él están las plantas que amas con todas tus fuerzas y pasarte el día cuidándolas y regándolas haría que estuvieras tremendamente satisfecho con tu vida”.

“Según lo que vimos en el ejercicio anterior, ¿cuáles serían tus plantas? ¿Puedes pensar por un momento en las cuatro plantas que son más importantes para ti?... ¿Cuáles son?”

“Si pudieras poner una nota a cómo de grandes, frondosas o bonitas están esas plantas en tu vida, ¿qué nota le pondrías de 0 a 10? ¿Podrías estar regando estas plantas y al mismo tiempo seguir en la lucha contra el papá y los hijos? ¿Cómo se quedarían estas plantas de 0 a 10 después de un año más de lucha contra el jefe y los “alguacilillos”? ¿Y después de 5 años?”

IDENTIFICACIÓN DE ACCIONES COTIDIANAS VALIOSAS Y BARRERAS: “Ahora, permíteme que veamos varias acciones que podrías comprometerte contigo mismo a realizar en tu día a día para cuidar tus plantas, y no haces porque estás enredado en tu mente” El paciente y el terapeuta exploran colaborativamente posibles acciones valiosas que sean concretas y factibles.

“Si hicieras esto día a día, ¿cómo estarían tus plantas dentro de 1 año? ¿Y dentro de 5 años?”

4. Cierre de la intervención

Hemos terminado las 3 sesiones programadas. Ahora te proponemos que sigas practicando. Para ello te vamos a enviar otro audio. La idea es que practiques durante al menos un mes unos 15-30 minutos diarios con todos los audios que te hemos enviado: unos días con unos y otros con otros. Lo ideal es que la práctica sea variada, así que, aunque tengas un audio que sea tu favorito, ve cambiando de día a día.

AUDIO 5

En este ejercicio te invitamos a centrarnos en las cosas que son importantes para ti, esas cosas que te gustaría hacer a lo largo de la vida y que harían que te sientas orgulloso de ti, entusiasmado e ilusionado porque te permiten acercarte a la persona que quieres ser y a la vida que quieres tener.

Para esto, imagínate que tu vida es como un camino, el cual día a día vas recorriendo. Hay días que avanzas mucho, otros avanzas poco y otros que seguramente te quedas quieto. Imagínate que al final del camino están las cosas importantes para ti, y cada una de ellas es una planta. Piensa por un momento en cuáles serían esas plantas y cómo de cuidadas están… (Dejar pausa de 15 segundos).

Puede que algunas de esas plantas estén ahora marchitas, otras tendrán algunas hojas caídas sin flores, otras estarán muy verdes, con frutos, otras tendrán mucha maleza alrededor… Permítete ver de qué depende que las plantas estén de una forma u otra. ¿Quién es la única persona que puede cuidar sus plantas? ¿Es posible que todas las plantas estén muy verdes y bonitas sin que nadie las cuide?

Como esas plantas están al final del camino, para cuidarlas cada día tienes que caminar hacia el jardín y realizar diferentes acciones para cuidarlas y nutrirlas. Hay días en que ni siquiera llegamos al final del camino… Otros días las abonamos, les movemos la tierra, las sacamos al sol, y cada una de estas acciones, a pesar de ser tan pequeñas y sencillas, ayudan a que a largo plazo esa planta esté como tú quieres que esté. Te invitamos a que elijas una de esas plantas, la que quieras, y por un momento pienses qué tipo de acciones podrías hacer en el día de hoy que supondría alimentar esa planta. Recuerda que a veces basta con acciones pequeñas y sencillas, pero realizadas de manera cotidiana. (Dejar 15 segundos)

Ahora imagínate que cada día haces pequeñas acciones en el cuidado de esa planta. ¿Cómo estaría tu planta en un mes? ¿Cómo estaría en un mes tu vida? ¿Sentirías que estás más cerca a la persona que quieres ser?

Sin embargo, a veces los caminos tienen piedras, pendientes, llueve… De vez en cuando, nos podemos tropezar con algún árbol caído o un charco grande de barro. Y es en esos momentos que caminar hacia las cosas que son importantes para nosotros, acercarnos a ser esa persona que queremos ser no es tan fácil… ¿Puedes imaginar qué obstáculos se pueden presentar en tu camino? ¿Qué dificultades puedes encontrar y con qué pensamientos te puedes enredar?

La cuestión es… Cuando estés caminando y te tropieces con algún obstáculo, tú puedes darte la vuelta y regresar a tu camino y avanzar a pesar del obstáculo o puedes desviarte

de tu camino, quedarte quieto, etc. ¿Quién elige hacer una cosa u otra? Seguramente, la opción más fácil es desviarse o quedarse parado porque avanzar a veces implica que tendrías que pasar por alguna incomodidad. Sin embargo, ¿merecería la pena pasar por esa incomodidad si con ello pudieras cuidar y nutrir tus plantas? ¿Merecería pena haber avanzado a pesar de las dificultades dentro de un mes, de un año o de 5 años?

Te invitamos a que durante el día de hoy estés atento a qué cosas podrías hacer para regar tus plantas y elijas a cada momento si quieres avanzar o quedarte anclado en tu camino.
